# POLARIS is a copper-binding peptide that interacts with ETR1 to negatively regulate ethylene signaling in *Arabidopsis*

**DOI:** 10.1016/j.xplc.2025.101432

**Published:** 2025-06-25

**Authors:** Anna J. Mudge, Saher Mehdi, Will Michaels, Beatriz Orosa-Puente, Weiran Shen, Charlie Tomlinson, Wenbin Wei, Claudia Hoppen, Buket Uzun, Dipan Roy, Flora M. Hetherington, Jennifer F. Topping, Ari Sadanandom, Georg Groth, Nigel J. Robinson, Keith Lindsey

**Affiliations:** 1 Department of Biosciences, Durham University, Durham DH1 3LE, UK; 2 Department of Chemistry, Durham University, Durham DH1 3LE, UK; 3 Institute of Biochemical Plant Physiology, Heinrich Heine University Düsseldorf, 40204 Düsseldorf, Germany

**Keywords:** plant hormone signaling, *arabidopsis*, ethylene, protein metalation, hormone receptor

## Abstract

Ethylene signaling is one of the classic hormonal pathways in plants, with diverse roles in development and stress responses. The dimeric ethylene receptor localizes to the endoplasmic reticulum and contains Cu(I) ions essential for ethylene binding and signal transduction. We previously discovered that mutants of the *Arabidopsis* gene *POLARIS* (*PLS*), encoding a 36-amino-acid peptide, exhibit enhanced ethylene signaling responses suggestive of reduced receptor activity, but the role and activity of the PLS peptide in this signaling cascade have not been defined. Here, we report that *Arabidopsis* PLS binds copper as a 1:2 thiol-dependent Cu(I):PLS_2_ complex with an affinity of 3.79 (±1.5) × 10^19^ M^−2^ via two cysteine residues conserved in the related species *Camelina sativa*. These residues are also essential for biological function. This affinity precludes a role for PLS as a cytosolic Cu chaperone. We demonstrate that PLS localizes to endomembranes and interacts with the transmembrane domain of the receptor protein ETR1. PLS–ETR1 binding is increased in the presence of copper, and this interaction provides a Cu-dependent mechanism for mediating the repression of ethylene responses. Because *PLS* transcription is upregulated by auxin and downregulated by ethylene, PLS–ETR1 interactions also provide a mechanism for modulation of ethylene responses in high-auxin tissues.

## Teaser

This study demonstrates that the POLARIS peptide of *Arabidopsis* localizes to endomembranes and interacts with the transmembrane domain of the ethylene receptor ETR1. PLS–ETR1 binding is increased in the presence of copper, and this interaction provides a copper-dependent mechanism for regulating ethylene responses.

## Introduction

Ethylene is a gaseous hormone used by plants to regulate many aspects of development and responses to biotic and abiotic stresses (Johnson and Ecker, 1998). It is perceived by a family of receptors that, in *Arabidopsis*, comprises five members located on the endoplasmic reticulum (ER) (Chen et al., 2002; Grefen et al., 2008): ETR1 (ETHYLENE RESPONSE 1), ERS1 (ETHYLENE RESPONSE SENSOR 1), ERS2, ETR2, and EIN4 (ETHYLENE-INSENSITIVE 4) (Chang et al., 1993; Hua et al., 1995; 1998; Sakai et al., 1998). The receptors are related to bacterial two-component systems (Chen et al., 2002), form dimers through disulfide bonding at the N-terminal hydrophobic domains (Schaller et al., 1995; Hall et al., 2000), and contain Cu(I) ions bound to residues Cys65 and His69, which are essential for ethylene binding and signal transduction (Rodriguez et al., 1999; McDaniel and Binder, 2012). In the absence of ethylene, these receptors activate the negative regulator CTR1 (CONSTITUTIVE TRIPLE RESPONSE 1), which is a mitogen-activated protein kinase kinase kinase (MAPKKK), thus preventing ethylene responses (Chang, 2003; Gao et al., 2003). The mechanisms by which receptor activity is regulated are not fully understood.

Introduction of copper to the ER and ethylene receptor requires the RAN1 (RESPONSIVE TO ANTAGONIST1) protein. This is a predicted copper-transporting P-type ATPase homologous to yeast Ccc2p and human Menkes and Wilson disease proteins (Hirayama et al., 1999). Strong *RAN1* loss-of-function mutants in *Arabidopsis* (e.g., *ran1-3*, *ran1-4*) exhibit an enhanced ethylene signaling response (Binder et al., 2010) consistent with a loss of receptor function and similar to that of higher-order loss-of-function receptor mutants, which also show an ethylene hypersignaling phenotype (Qu et al., 2007). The mechanisms of copper homeostasis at ETR1 are unknown, as is true for other compartmentalized cuproproteins supplied with copper, for example, via Ccc2p, Menkes, or Wilson ATPases. RAN1 in *Arabidopsis* localizes to endomembrane systems, including the *trans*-Golgi and ER compartments, and is necessary for both ethylene-receptor biogenesis and copper homeostasis; loss-of-function *ran1* mutants suggest that copper is required for both ethylene binding and receptor function (Binder et al., 2010). RAN1 can interact directly with ETR1 and the copper chaperones ANTIOXIDANT1 (ATX1) and COPPER CHAPERONE (CCH), suggesting that copper is transported between proteins to deliver it to the ethylene receptor as part of the receptor biogenesis pathway at the ER (Hoppen et al., 2019).

Our understanding of receptor function is still incomplete, however. For example, how is Cu(I) delivery from RAN1 to ETR1 mediated? How does Cu(I) influence receptor conformation and function? Are there other Cu(I)-binding components involved? Is the receptor regulated in a tissue-specific manner or in response to the hormonal environment in a tissue? Is this process part of the crosstalk mechanism with other hormone signaling pathways? It is well established that ethylene signaling interacts with and is affected by other hormonal pathways, but does this influence the receptor metalation state and have developmental consequences?

We previously showed that the loss-of-function *polaris* (*pls*) mutant has some phenotypic similarities to *ran1* loss-of-function alleles and to *ctr1*, exhibiting a triple-response phenotype (short hypocotyl and root, exaggerated apical hook, radial expansion) in the dark in the absence of ethylene (Chilley et al., 2006) and a short-root phenotype in light-grown seedlings, consistent with its known expression in the root meristem of light-grown seedlings. Transgenic complementation of the mutant by the *PLS* gene (AT4G39403), which encodes a 36-amino-acid peptide, suppresses the mutant phenotype (Casson et al., 2002). The *pls* mutant phenotype is rescued by the gain-of-function ethylene-resistant mutation *etr1-1* and by pharmacological inhibition of ethylene signaling by silver ions (Chilley et al., 2006). The *pls* mutant produces ethylene gas at wild-type levels, indicating that the peptide plays a role in ethylene signaling rather than ethylene biosynthesis (Chilley et al., 2006). By contrast, *PLS* transgenic overexpressors (PLSOx seedlings) exhibit suppression of the triple-response phenotype when grown in the presence of the ethylene precursor 1-aminocyclopropane-1-carboxylic acid (ACC), similar to the gain-of-function *etr1-1* mutant, but this suppression is incomplete (PLSOx seedlings show some response to ACC; Casson et al., 2002; Chilley et al., 2006). *PLS* overexpression also partially suppresses the *ctr1* mutant phenotype, indicating that the PLS peptide acts upstream of CTR1 (Chilley et al., 2006).

In this paper, we show that the POLARIS peptide is required for correct ethylene responses, binds copper via two cysteine residues essential for biological function, but is unlikely to act as a metallochaperone intermediary; however, it co-localizes and forms a copper-adduct with ETR1. We suggest that POLARIS provides cell-type control and a crosstalk regulatory mechanism for ethylene responses through its physical interaction with the ethylene receptor.

## Results

### The POLARIS peptide is a negative regulator of ethylene responses

**Figure 1:**
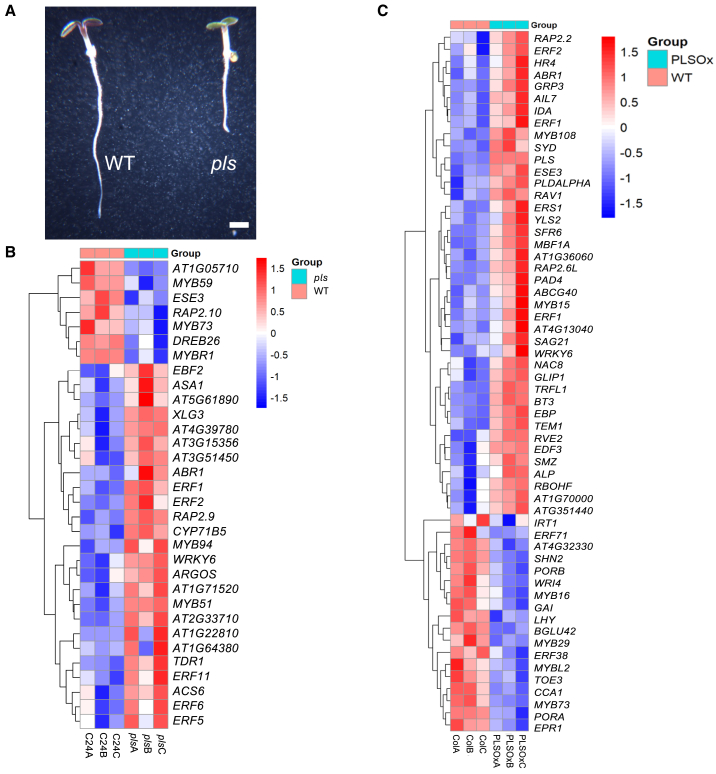
The PLS peptide is required for ethylene control of seedling growth. **(A)** Wild type (ecotype C24, left) and *pls* mutant (in C24 background, right). Scale bar corresponds to 5 mm. **(B and C)** Heat maps showing expression levels of 32 ethylene-responsive genes in *pls***(B)** and 58 genes in *PLS* overexpressing (PLSox in Col-0 background) seedlings **(C)** compared with wild-type (Col-0) levels. Data for three biological replicates (**A**, **B**, and **C**; independent seedling samples from which RNA was extracted and its expression analyzed and used for statistical analysis) are shown and are expressed as log_2_-fold changes in *pls* mutant and PLSox seedlings compared with the wild type, with corresponding significance levels (*P*-adj values) provided in supplemental Tables 1 and 2. *P* < 0.05 and log_2_(fold change) of ±0.5 were used to identify differentially expressed genes.

The PLS peptide is translated from a low-abundance transcript that, in light-grown seedlings, is most strongly expressed in the embryo and silique, seedling root tip, and leaf vascular tissues (supplemental Figure 1), a pattern reflected in promoter–reporter expression patterns (Casson et al., 2002). Seedlings of the *pls* mutant have short roots in the light in air (Figure 1A). They also have shorter hypocotyls and roots than the wild type and have an exaggerated apical hook when grown in the dark in air but are similar to the wild type in the presence of the ethylene precursor ACC (supplemental Figure 2), indicative of an ethylene hyper-responsive phenotype (Chilley et al., 2006). To investigate the *pls* molecular phenotype, we performed an RNA sequencing (RNA-seq) analysis of the loss-of-function *pls* mutant and transgenic PLSOx seedlings to identify differentially regulated genes, comparing each with wild-type seedlings of the same ecotype as controls (Figure 1B and 1C). The *pls* mutant does not express the full-length *PLS* coding sequence owing to a T-DNA insertion in the coding region and therefore shows disrupted PLS function (Casson et al., 2002). By contrast, the PLSOx seedlings express significantly higher levels of *PLS* transcript compared with the wild type (log_2_ fold 10.8, *p-*adj = 2.02 × 10^−33^; supplemental Tables 1 and 2). Eight hundred and thirty-six genes were significantly upregulated and 292 downregulated in the *pls* mutant compared with wild-type control seedlings (*p-*adj < 0.05) (supplemental Table 1). A total of 1487 genes were significantly upregulated and 1281 downregulated in PLSOx seedlings compared with wild-type controls (*p-*adj < 0.05; supplemental Table 2). Gene Ontology (GO) analysis of genes upregulated in *pls* mutant seedlings compared with wild-type seedlings showed significant enrichment of genes associated with responses to hormone signaling, biotic and abiotic defense responses, and cell death (supplemental Table 3).

Out of 307 genes annotated with the GO term GO:0009723 (response to ethylene), 25 were significantly upregulated and 7 downregulated in the *pls* mutant compared with the wild type, and 40 were upregulated and 18 downregulated in the PLSOx seedlings (Figure 1C; supplemental Tables 3, 4, 5, and 6; Mudge et al., 2024), indicating that control over *PLS* expression levels is required for correct ethylene responses. While GO:0009723 (response to ethylene) was significantly enriched in genes upregulated in the *pls* mutant compared with the wild type (FDR *=* 0.00062; supplemental Table 3), a large number of upregulated genes in *pls* were significantly associated with immunity, response to pathogens, and the hypersensitive response (supplemental Table 3). Downregulated genes in *pls* were significantly enriched in GO terms such as hormone biosynthetic process (GO:0042446, FDR = 0.038), hormone metabolic process (GO:0042445, FDR = 0.0096), and regulation of hormone levels (GO:0010817, FDR = 0.0013) (supplemental Table 4; Mudge et al., 2024), consistent with our previous studies that described PLS-dependent crosstalk among ethylene, auxin, and cytokinin signaling (Liu et al., 2010; 2013; Moore et al., 2015). In PLSOx overexpressors, both up- and downregulated genes included those associated with GO terms such as “hormone response,” “stress response,” and “immune response.” Genes associated with “photosynthesis” were downregulated in PLSOx seedlings, consistent with repressed photosynthetic activity in the root tips (supplemental Tables 5 and 6; Casson et al., 2002). Interestingly, when comparing the 120 most significantly differentially expressed GO terms upregulated in the *pls* mutant vs. wild type with the same GO terms in PLSOx vs. wild type, we found that most GO terms were restored to wild-type levels (i.e., nonsignificant *P* values between PLSOx and the wild type), although some terms were—notably, “response to stimulus” and “response to abiotic stimulus,” among others (summarized in supplemental Table 7). Four candidate PLS-regulated ethylene-responsive genes identified in the RNA-seq data—*ERF11* (AT1G28370), *ERF19* (AT1G22810), *ERF61* (AT1G64380), and *TDR1* (AT3G23230)—were validated by RT–qPCR and showed consistent upregulation in the *pls* mutant with either nonsignificant or slight downregulation in PLSOx seedlings (supplemental Figure 3). *ERF19*, *ERF61*, and *TDR1* encode integrase-type transcription factors, members of the AINTEGUMENTA-like (AIL) subfamily of the AP2/EREBP family essential for meristem function and stress tolerance (Ma et al., 2024). These data are consistent with a role for PLS in a number of ethylene responses and other related signaling, stress, and developmental processes.

Peptides with high sequence similarity to PLS are found in the Brassicaceae. BLAST searches using the 36-amino-acid PLS peptide sequence as a query identified peptides with significant alignments in *Arabidopsis lyrata* (35 amino acids), *Camelina sativa* (22 amino acids), *Eutrema salsugineum* (28 amino acids), and *Raphanus sativa* (34 amino acids) (supplemental Figure 4) and 36-amino-acid peptides with <50% alignment in *Cardaminopsis suecica* and the hybrid *A. thaliana* × *A. arenosa*. Structural predictions using AlphaFold2 indicated limited structural conservation, but it has been demonstrated that AlphaFold2 is poor at predicting peptide structures (McDonald et al., 2023), and these models should therefore be interpreted with caution.

**Figure 2:**
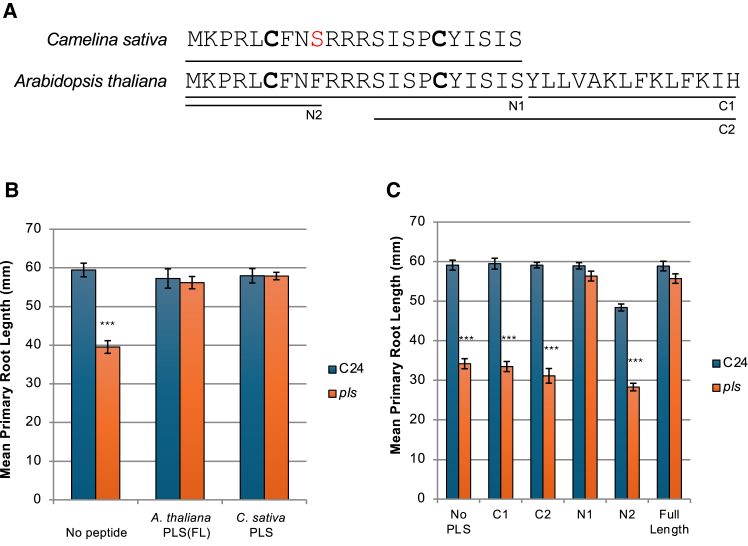
The PLS peptide exhibits structural and functional conservation in Brassicaceae. **(A)** Amino acid sequence of the PLS peptide from *Arabidopsis thaliana* with the *Camelina sativa* PLS sequence and the synthetic truncations N1, N2, C1, and C2 indicated by horizontal lines. Two cysteine residues are highlighted in bold. **(B)** Effect of the synthetic *Arabidopsis* PLS full-length peptide, *A. thaliana* PLS(FL), and the *Camelina* PLS peptide (*C. sativa* PLS) on *Arabidopsis* primary root length. Wild-type (C24; blue bars) and *pls* mutant seedlings (orange bars) were grown hydroponically in the presence (100 nM) or absence of peptide for 10 days. ∗∗∗*P* < 0.05, *t*-test between C24 and *pls*. Error bars show ±1 standard error of the mean, *n* = 20. **(C)** Effect of synthetic full-length and truncated peptides on primary root length of wild-type (blue bars) and *pls* mutant (orange bars) *Arabidopsis*. Seedlings were grown hydroponically in the presence of 50 nM peptide for 10 days. C1, C-terminal 14 amino acids; C2, C-terminal 24 amino acids; N1, N-terminal 22 amino acids; N2, N-terminal 9 amino acids; full-length PLS, 36 amino acids. Error bars show ±1 standard error of the mean, *n* = 25. ∗∗∗*P* < 0.05, *t*-test between C24 and *pls*.

To better understand the relationship between PLS peptide structure and function between species, we performed hydroponic feeding experiments using synthetic versions of the PLS peptide from *Arabidopsis* and its close relative *C. sativa*. The *C. sativa* gene shows partial sequence identity to the *Arabidopsis PLS* gene, and its predicted 22-amino-acid peptide sequence is identical to the N-terminal 22 amino acids of the *Arabidopsis* PLS, except for a phenylalanine-to-serine substitution at position nine (Figure 2A). We synthesized the full-length PLS peptide, PLS(FL), as well as truncated versions from both *Arabidopsis* and *C. sativa* (Figure 2A), and supplied the peptides hydroponically to *Arabidopsis pls* mutant seedlings. The full-length peptides from both *Arabidopsis* and *C. sativa* and the N-terminal 22-amino-acid sequence of the *Arabidopsis* peptide (N1) were each able to rescue the short primary root length of the *Arabidopsis pls* mutant (Figure 2B); these results were similar to those obtained by transgenic overexpression and genetic complementation using the wild-type *PLS* coding sequence (Casson et al., 2002; Chilley et al., 2006). It is interesting to note that although the predicted structure of the *C. sativa* peptide is quite dissimilar from that of the *Arabidopsis* peptide (supplemental Figure 4), it still retains function in *Arabidopsis.* The mean primary root length of wild-type (C24) seedlings did not differ across all peptide treatments, whereas treatment of *pls* seedlings with 50 nM PLS(FL) and N1 peptides rescued root growth (Figure 2C), and *t*-tests showed that the rescue effects of PLS(FL) and PLS(N1) did not differ significantly (*P* > 0.1, *n* = 25). However, neither a 9-amino-acid sequence (N2, Figure 2C) from the N terminus nor C-terminal sequences of 14 (C1) or 24 (C2) amino acids from *Arabidopsis* PLS were able to rescue the mutant (Figure 2C); each of these shorter peptides lacked one of the two Cys residues found in the functional longer peptides PLS(FL) and PLS(N1). A fluorescently tagged (5-carboxyfluorescein [5-FAM]) version of the *Arabidopsis* N-terminal 22-amino-acid sequence (N1) was taken up by the roots and also rescued the mutant root phenotype (supplemental Figure 5A and 5B).

### PLS localizes to endomembranes, including the endoplasmic reticulum

**Figure 3:**
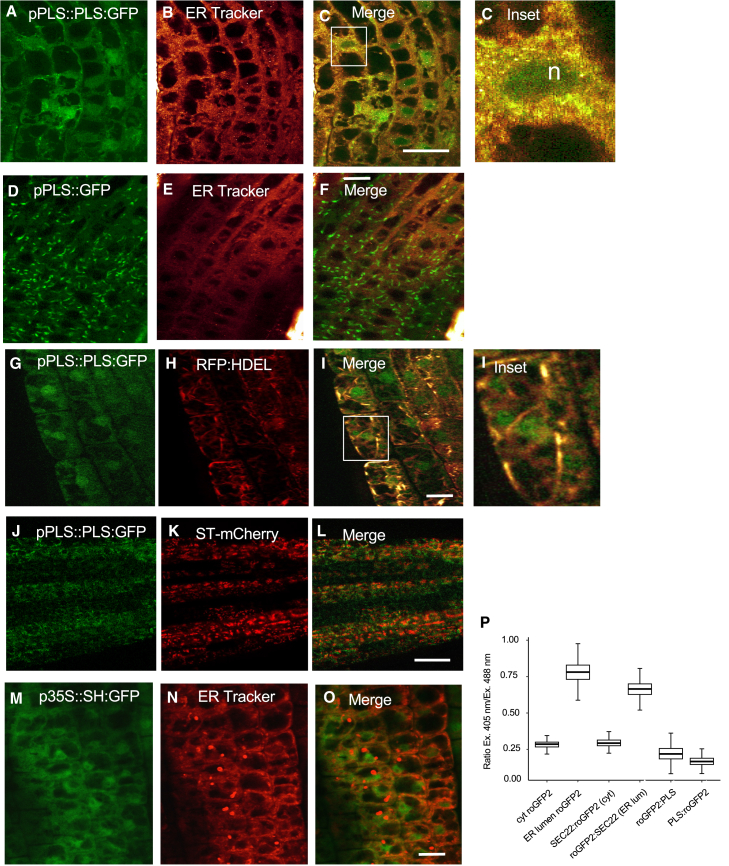
PLS localizes to the endoplasmic reticulum. **(A–O)** PLS::PLS:GFP fusion protein localization in stably transformed *Arabidopsis* plants. **(A, G, and J)** PLS::PLS:GFP co-localizes with the endoplasmic reticulum markers ER Tracker **(B, C, and C** inset**)** and RFP:HDEL **(H, I, and I** inset**)**, but SH:GFP (which is cytoplasmically localized) does not **(D–F and M–O)**. PLS:GFP fluorescence is seen also in nuclei (n). PLS::PLS:GFP **(J)** does not co-localize with the *trans*-Golgi marker ST-mCherry **(K and L)**. Scale bars correspond to 25 μm **(C and L) and** 10 μm **(F, I, and O)**. Root epidermal cells in the transition zone were imaged. **(P)** Ratiometric analysis of roGFP2 fusion constructs transiently expressed in *N. benthamiana*. Comparison of the excitation ratios of PLS-roGFP2 and roGFP2-PLS with control constructs (free roGFP2, SEC22 fusions) reveals that PLS localizes at least predominantly to the cytosolic side of the ER.

Because genetic studies suggest that PLS acts close to the ethylene receptor (Chilley et al., 2006), we hypothesized that it would localize to the same subcellular compartment as ETR1. The ethylene receptor in *Arabidopsis* is localized predominantly at the ER (Chang, 2003), and a *proPLS::PLS:GFP*-generated fusion protein (PLS:GFP) was used to investigate sub-cellular localization. Of five independent transformants with a single-copy insertion of the *proPLS::PLS:GFP* gene in the *pls* mutant background, four fully complemented the *pls* mutant (supplemental Figure 6), similar to the wild-type cDNA (Casson et al., 2002), demonstrating the functionality of the gene fusion. PLS:GFP in transgenic plants co-localized with the ER marker dye ER-Tracker Red, which binds sulfonylurea receptors of ATP-sensitive channels on the ER (ThermoFisher; Figure 3A–3C). It also co-localized with the ER lumen–targeted red fluorescent protein RFP:HDEL (Lee et al., 2013) (Figure 3G–3I). These observations suggest that PLS:GFP is located both on the ER membrane and in the lumen. PLS:GFP also appeared to localize to the nucleus and cytoplasm (Figure 3C). As a control, free GFP expressed under the control of the *PLS* promoter did not co-localize to the ER (Figure 3D–3F), and, as expected, the Golgi marker SH:GFP did not co-localize with ER Tracker (Figure 3M–3O). Visualization of *trans*-Golgi-localized SULFOTRANSFERASE1 (ST1) mCherry (Bauer and Papenbrock, 2002) showed that PLS:GFP did not localize to the Golgi (Figure 2J–2L). To identify the side of the ER membrane to which PLS localizes, we performed transient expression of redox-sensitive GFP (roGFP2) fusions of PLS (driven by the CaMV35S promoter, Hoppen et al., 2019). The different excitation properties of roGFP2 in an oxidizing (ER lumen) or reducing environment (cytosol) enabled us to determine the precise location of PLS fused to roGFP2. Ratiometric analysis and comparison with proteins of known localization (i.e., cytosolic roGFP2, ER luminal roGFP2, cytosolic v-SNARE SEC22:GFP, and ER luminal roGFP2:SEC22; Brach et al., 2009) revealed that PLS, as either an N- or C-terminal roGFP2 fusion, resided at the cytosolic side of the ER (as well as other vesicular compartments; Figure 3P). However, there may have been some translocation of PLS:GFP into the ER lumen, as indicated by co-localization with RFP:HDEL. Although the PLS:GFP fusion protein is relatively large compared with the PLS peptide, and this may affect its localization, the fusion was able to complement the *pls* mutation, showing that it is biologically functional. Although it is also formally possible that the GFP moiety is cleaved from the fusion and that the native PLS peptide acts to complement the mutant, the PLS:GFP localization pattern was more specific than that of free GFP or SH-GFP, suggesting that the majority of the fusion peptide localized to the indicated membrane compartments (Figure 3M–3O).

### PLS interacts with the ethylene receptor protein ETR1

**Figure 4:**
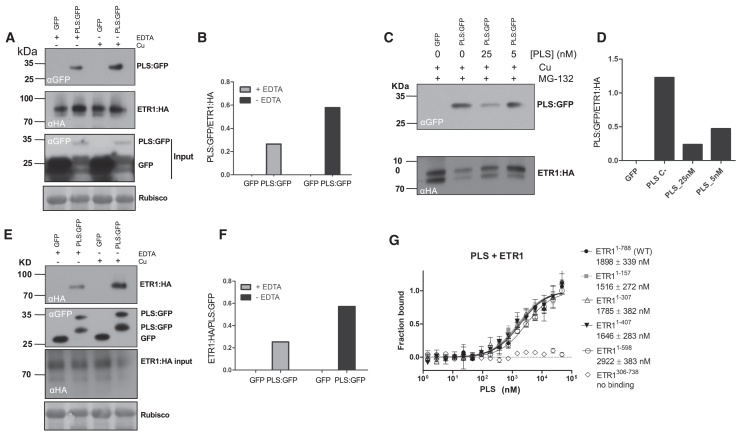
PLS interacts with the ethylene receptor ETR1. **(A)** Co-immunoprecipitation of PLS:GFP by ETR1:HA (anti-HA beads, upper panel) in leaves of *N. benthamiana* in the presence and absence of 0.5 μM CuSO_4_ and EDTA (to remove Cu). Lower panels show the presence of ETR1:HA in extracts using anti-HA antibody (αHA) and protein input blots using anti-GFP (αGFP) and anti-Rubisco (Rubisco) antibodies. **(B)** Densitometry scan of immunoblot. **(C)** Competition assay showing a reduced binding between PLS:GFP and ETR1:HA in the presence of 0, 5, or 25 nM PLS peptide, in the presence of 0.5 μM CuSO_4_ and 50 μM MG-132, a proteasome inhibitor (upper panel). Lower panel shows ETR1:HA in extracts using anti-HA (αHA) antibody. **(D)** Densitometry scan of immunoblot. **(E)** Co-immunoprecipitation of ETR1:HA by PLS:GFP (anti-GFP beads, upper panel) or PLS:GFP and GFP using anti-GFP beads (second panel) in leaves of *N. benthamiana*, showing the effect of EDTA (to remove Cu) on the interaction between ETR1:HA and PLS:GFP. Lower two panels show the presence of ETR1:HA in protein input blots using anti-ETR1:HA (αHA) and anti-Rubisco (Rubisco) antibodies. αHA, anti-HA antibody beads; αGFP, anti-GFP antibody beads. Appropriate anti-HA, anti-GFP, and anti-Rubisco antibodies were used for protein visualization. **(F)** Densitometry scan of immunoblot in **(E)**. **(G)***In vitro* microscale thermophoresis binding curves of different recombinant ETR1 truncations with PLS. Binding of PLS was observed with full-length ETR1 and all C-terminal truncations but not with ETR1^306−738^, which lacked the N-terminal transmembrane part of the receptor. The values shown in the figure represent the binding constants of PLS with the indicated ETR1 constructs. Superscript numbers in the construct names denote amino acid positions within the protein. The values are shown with their respective standard deviations.

We hypothesized that PLS plays a role in receptor function and investigated whether this involved direct interaction with the receptor complex. Preliminary yeast 2-hybrid analyses suggested that PLS interacts with ETR1 (supplemental Figure 7). Because there may be some PLS:GFP localization to the nucleus (Figure 3), it is possible that some ETR1 and native PLS also localize to the nucleus to account for the interaction in a yeast 2-hybrid assay. Confirmation of the physical interaction between PLS and ETR1 in plants came from co-immunoprecipitation (CoIP) analysis. *Agrobacterium* containing plasmids encoding PLS linked to a C-terminal GFP, GFP without PLS (control), and ETR1 with a C-terminal HA tag were infiltrated into *Nicotiana benthamiana* leaves for transient expression, each gene under the control of the CaMV35S promoter. After 3 days, the interaction was confirmed by western blotting after CoIP, showing that ETR1:HA was expressed in all samples and that ETR1:HA bound to PLS:GFP. The GFP-only controls did not show binding to ETR1 (Figure 4A). This demonstrates that the interaction is dependent on the presence of the PLS peptide. The addition of 0.5 μM copper sulfate to the protein extract used for CoIP experiments stabilized the PLS–ETR1 interaction. The presence of copper ions resulted in almost three-fold more PLS:GFP detected upon pulldowns with ETR1:HA, or conversely of ETR1:HA pulled down with PLS:GFP, compared with the same assay in the presence of the metal chelator 2 mM EDTA (Figure 4A, 4B, 4E, and 4F).

To investigate the specificity of PLS binding, synthetic PLS(FL) was introduced into infiltrated *N. benthamiana* leaves 30 min before tissue harvest in the presence of both copper to maximize PLS–ETR1 interaction and the proteasome inhibitor MG-132 to prevent protein degradation. The addition of 25 nM synthetic PLS caused an ∼80% reduction in PLS:GFP binding to ETR1:HA (Figure 4C and 4D), suggesting that the synthetic PLS peptide competed for ETR1 binding, showing the specificity of PLS for ETR1. The anti-GFP beads bound two sizes of PLS-GFP protein (Figure 4E), both of which were larger than a GFP-only control, suggesting that the PLS peptide undergoes cleavage, a change in conformation, post-translational modification, or incomplete reduction of Cys residues on some PLS; similarly, free GFP was also seen in the input samples from *N. benthamiana* leaves (Figure 4A, inset panel). When ETR1:HA was used to pull down PLS-GFP, only the larger peptide was present (Figure 4A), suggesting that ETR1 binds a longer version of the PLS peptide.

To pinpoint the interaction site at the receptor in more detail, *in vitro* binding studies were performed with purified receptor variants and PLS by microscale thermophoresis (Figure 4G). Binding of PLS was observed only with receptor variants containing the N-terminal transmembrane domain. By contrast, no binding was detected with ETR1 that lacked this domain (ETR1^306−738^). The terminal transmembrane domain harbors the ethylene and copper binding region (Schott-Verdugo et al., 2019).

### PLS binds Cu(I)

**Figure 5:**
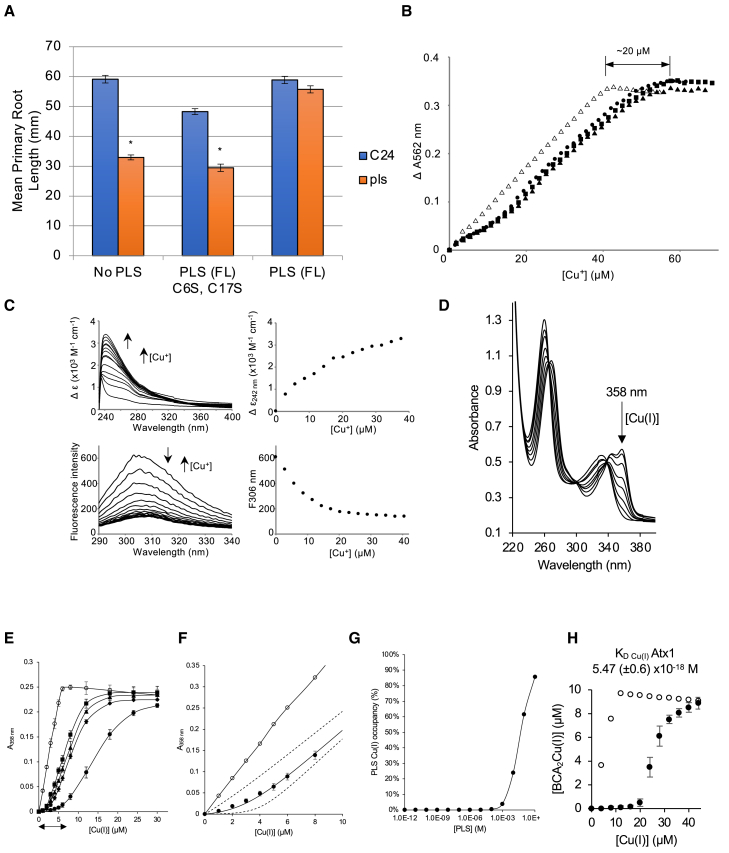
PLS binds copper. **(A)** C6S and C17S are required for PLS function. Seedlings were grown hydroponically for 10 days in the presence (100 nM) or absence of peptide (FL or C6S, C17S). Blue bars are the wild type (C24), and orange bars are *pls*. ∗*P* < 0.05, *t*-test between C24 and *pls*, *n* = 25. Error bars show ±1 standard error, *n* = 14. **(B)** Bicinchoninic acid (BCA) absorbance in the presence of synthetic PLS (44.8 μM, filled symbols) or an equivalent volume (to PLS) of DMSO (open symbols). Experiments with PLS used 88.3 μM (circles), 84 μM (triangles), and 88.4 μM (squares) BCA. The control experiment with DMSO used 85.3 μM BCA. **(C)** UV–vis apo subtracted difference spectra of synthetic PLS (19.8 μM) upon titration with CuCl (top left), and binding isotherm of the feature at 242 nm (top right). Fluorescence spectra of PLS (19.8 μM) upon titration with CuCl (bottom left), and binding isotherm of the feature at 306 nm (bottom right). The first arrow indicates an increase or decrease in intensity (upward or downward, respectively) with increasing (second upward arrow) addition of Cu(I). **(D)** Absorbance of BCA (17.3 μM) titrated with Cu(I) (representative spectrum, *n* = 2, full data in supplemental Figure 8). **(E)** Binding isotherms (A_358 nm_) of BCA (10 μM) in the presence/absence (filled/empty symbols) of 14 μM MBP:PLS (circles) or MBP:PLS mutants (C6S, C17S, and C6S/C17S: triangles, diamonds, and squares, respectively) titrated with Cu(I) (*n* = 3, ±SD). Arrow indicates ∼7 μM withholding of Cu(I) from BCA. **(F)** Binding isotherms (A_358 nm_) of BCA (50 μM) in the presence/absence of 10 μM MBP:PLS (filled/empty symbols) titrated with Cu(I). Model (solid line) describes Cu(I) binding as a 2:1 complex, with a *β*_*2*_ affinity of 3.79 (±1.5) × 10^19^ M^−2^. Dotted lines simulate 10× weaker or tighter affinity (*n* = 3, ±SD). **(G)** Simulated Cu(I) occupancy (calculated as described in the supplemental text) as a function of [PLS] using a *β*_*2*_ Cu(I) affinity of 3.79 × 10^19^ M^−2^ and conditions matching Cu(I) availability at 50% ATX1 saturation from a determined KD Cu(I) ATX1 (supplemental Figure 12). **(H)***Arabidopsis* ATX1 (20 μM, filled circles) withholds one Cu(I) equivalent from 20 μM BCA (open circles, BCA alone) (*n* = 3, ±SD), with *K*_*D*_ Cu(I) 5.47 × 10^−18^ M (supplemental Figure 12).

Cysteine residues are common metal-ligand binding residues in low-molecular-weight copper-handling peptides, and predictions of PLS structure (supplemental Figure 4) suggest a single α-helix plus an unstructured region with two cysteines (CX_10_C arrangement, where X is any amino acid) with some analogy to copper-metallochaperones such as Cox17 or other CX_9_C twin proteins (Robinson and Winge, 2010; Yamasaki et al., 2009; Zhang and Li, 2013; Zhang et al., 2014). In view of both structural considerations and the copper dependency of ETR1, we examined whether the two cysteine residues (C6 and C17) in PLS play a functional role by analyzing both *pls* mutant complementation and copper binding by PLS peptide variants. A mutated *Arabidopsis* full-length peptide in which both cysteines were replaced with serines, PLS(FL C6S, C17S), was non-functional in hydroponic root-feeding assays, failing to rescue the short primary root phenotype of the *pls* mutant (Figure 5A). Furthermore, as indicated above, the 9-amino-acid sequence N2 and the C-terminal sequences C1 and C2 from *Arabidopsis* PLS, which each contain only one cysteine residue, were unable to rescue the mutant (Figure 2C). These results indicate that the two cysteine residues are required for biological activity, measured as primary root growth. Interestingly, RNA-seq data showed that 19 out of 287 genes associated with the GO term response to metal ion (GO:0010038) were also significantly downregulated in the *pls* mutant (supplemental Table 4, enrichment FDR = 0.000057). All 19 genes are associated with stress responses, including oxidative, salt, metal, and osmotic stress. For example, AT5G14545 (MIR598b) and AT4G25100 (encoding a Fe-superoxide dismutase) are both major targets of the Cu deficiency response regulator SQUAMOSA-PROMOTER BINDING PROTEIN-LIKE 7 (SP7) (Yamasaki et al., 2009; Zhang and Li., 2013; Zhang et al., 2014), which also interacts with and regulates RAN1 in ethylene signaling and is feedback-regulated by ethylene signaling (Yang et al., 2022). AT3G03780 encodes a methionine synthase (linked to ethylene biosynthesis), and AT3G56240 encodes the CCH copper chaperone (supplemental Table 8).

To determine a possible role for PLS and the cysteine residues in binding Cu(I), synthesized PLS peptide was titrated with Cu(I) ions under strictly anaerobic conditions and monitored for copper-dependent spectral features (Figure 5B–5D). Both the UV–vis (putative metal-to-ligand charge-transfer) and fluorescence (putative tyrosine solvent access) spectra of PLS changed as a function of Cu(I) ion concentration, consistent with complex formation (Figure 5C). We titrated PLS peptide against bicinchoninic acid (BCA) (Figure 5B), which is a chromophore that binds Cu(I) (Xiao et al., 2011; Figure 5D and supplemental Figures 8 and 9). The rationale was to determine whether PLS could compete for Cu(I) with BCA, which would be indicative of binding by PLS. PLS peptide was titrated with Cu(I) in the presence of 87 μM BCA. The presence of PLS peptide (44.8 μM) increased the concentration of Cu(I) required to saturate BCA by ∼20 μM (Figure 5B), and the initial gradient was shallower than in the control reaction, which contained BCA and Cu(I) but lacked PLS. Thus, PLS withholds Cu(I) from BCA, implying that its affinity for Cu(I) (*K*_Cu_ PLS) is tighter than that of BCA (*K*_Cu_ BCA); these data also support a 2:1 stoichiometry of binding to the tightest site. Titration experiments also showed that although synthetic PLS(FL) withholds Cu(I) from BCA, mutant PLS peptide (FL C6S, C17S) does not (supplemental Figure 10), confirming a role for the cysteine residues in Cu(I) binding. Because problems with PLS solubility could potentially reduce the accuracy with which we determined the stoichiometry and affinity for Cu(I) (complete solubility is required for precise affinity quantification), we also tested a version of PLS fused to maltose binding protein (MBP:PLS), which retained solubility when titrated with Cu(I) ions under strictly anaerobic conditions. MBP:PLS (14 μM) was found to withhold ∼7 μM Cu(I) from BCA, again indicative of a 2:1 PLS:Cu(I) stoichiometry, and tight binding was cysteine dependent (Figure 5E). A *β*_*2*_ affinity of 3.79 (±1.5) × 10^19^ M^−2^ was determined by competition against an excess of BCA, and the fit significantly departed from simulations 10× tighter or weaker (Figure 5F and supplemental Figure 11).

Metal binding in biology is challenging to predict because the formation of metal-protein complexes is a combined function of metal affinity for a given protein and metal availability, which would need to be known for Cu(I) in the *Arabidopsis* cytosol in the case of PLS (assuming the same orientation of binding residues as with the GFP tag; Figure 3P). Cu(I) occupancy of the cytosolic copper chaperone ANTIOXIDANT PROTEIN 1 (ATX1) tracks fluctuations in available cytosolic Cu(I) such that its affinity approximates to the mid-point of the range of Cu(I) availabilities within this eukaryotic compartment (Yu et al., 2017; Morgan et al., 2019). *Arabidopsis* ATX1 was therefore expressed and purified to determine a 1:1 ATX1:Cu(I) stoichiometry and an affinity *K*_D Cu(I)_ of 5.47 (±0.6) × 10^−18^ M (Figure 5H and supplemental Figure 12). The mid-point availability was thus estimated to be 5.47 (±0.6) × 10^−18^ M, noting that this number informs how tightly (as an activity) a labile, ligand-exchangeable pool is bound rather than some negligible concentration of hydrated Cu(I) (for simplicity, we have not calculated free energies for complex formation here). Figure 5F and 5G reveal that the cytosolic concentration of PLS would need to exceed (improbable) millimolar concentrations for Cu(I)-dependent homodimers to form at this cytosolic Cu(I) availability (mathematical models and equations are shown in supplemental text). It is thus unlikely that the Cu(I):PLS_2_ complex alone delivers Cu(I) to, or retrieves it from, the interacting cuproprotein ETR1. Cu(I)-dependent PLS–ETR1 heterodimeric complexes are the more likely functional species, and we conclude that PLS binding alters either ETR1 conformation or ethylene availability to regulate receptor activity.

## Discussion

We present evidence for a role of the PLS peptide as a new Cu(I)-binding peptide that, on the basis of its physical interactions with the receptor and its ethylene-signaling function as revealed by genetic and physiological experiments, acts as a regulatory component of the ethylene signaling pathway. Peptides can act as ligands for receptor kinases to regulate signaling pathways, such as in plant immunity (Campos et al., 2018), development (Willoughby and Nimchuk, 2021), or responses to abiotic stresses (Kim et al., 2021). We propose that the PLS peptide is a new component of the regulatory mechanism for ethylene receptor function linked to its copper-binding activity (Rodriguez et al., 1999). Loss of copper binding through mutation of the two cysteines in PLS ablates its biological activity (Figures 2C, 5A, and 5E; supplemental Figure 10). The *pls* mutant phenotype has some similarities (enhanced ethylene responses) to that of strong *ran1* mutant alleles in which copper delivery to the receptor is compromised (Binder et al., 2010). It also shares (statistically significant) overlap in differentially expressed genes (DEGs) with the *ctr1* mutant: 11.9% of the *ctr1* upregulated DEGs are shared with *pls* (each compared with its respective wild type; supplemental Figure 13).

Compared with the wild type, the *pls* mutant shows significant upregulation of genes associated with diverse GO terms that include responses to biotic and abiotic stimuli and plant immunity, which in turn are regulated by hormonal systems that include ethylene signaling. However, an intriguing feature of *PLS* overexpression is that there was no significant downregulation of these gene categories to levels below those seen in the wild type. Rather, their expression returned to wild-type levels but was not repressed further. For example, although *ESE3* was upregulated in the *pls* mutant and downregulated in PLSOx seedlings, other ethylene-responsive genes were either upregulated in both genotypes or restored to wild-type levels in PLSOx (supplemental Tables 1, 2, 3, and 4). This suggests that *PLS* overexpression does not have a significant additional biological effect beyond restoration to wild-type levels, consistent with the lack of a strong seedling phenotype in light-grown PLSOx seedlings (although they do show reduced sensitivity to ACC inhibition of root growth; Casson et al., 2002; Chilley et al., 2006). One possible interpretation is that saturation of the PLS–receptor interaction occurs over a small concentration range of the PLS peptide. It is also likely that crosstalk between ethylene and other signaling pathways influences the output of these interactions when measured as gene expression levels or patterns (Moore et al., 2024), and so some pleiotropic effects of the *pls* mutation may be seen.

Many plant peptides involved in signaling are processed from longer pre-proteins (Olsson et al., 2019). By contrast, PLS is translated from an open reading frame to produce a functional 36-amino-acid peptide; the peptide appears to be cleaved or otherwise modified in the cell (Figure 4E). It is possible that the three arginines (amino acids 10–12) may represent a cleavage site (Duckert et al., 2004; Capraro et al., 2015), which would produce the observed shorter GFP fusion protein (∼30 kDa) detected in anti-GFP CoIP blots (Figure 4E). The size of this PLS:GFP cleavage product suggests that this peptide fragment represents GFP (∼27 kDa) plus a 3 kDa fragment of the C-terminal region of PLS. Such cleavage would likely inactivate PLS function, as we found that shorter N- and C-terminal fragments (each with only one cysteine residue) were not biologically active (Figure 2C). PLS:GFP localizes to several subcellular compartments (although not to the *trans*-Golgi), including the ER, where the ethylene receptor is found (Figure 3). Furthermore, PLS can interact directly with the receptor protein ETR1 (and specifically with the N-terminal copper-binding transmembrane domain of ETR1).

Previously, a model was proposed for copper delivery into the cell via the COPT family of transporters, which are located at the root plasma membrane (Chen et al., 2022), to RAN1 via ATX1 and/or CCH (Li et al., 2017; Hoppen et al., 2019). *Arabidopsis* has three known copper chaperones, ATX1, CCH, and a copper chaperone for superoxide dismutase, CCS (Casareno et al., 1998; Chu et al., 2005; Puig et al., 2007). These are required for copper homeostasis, and both ATX1 and CCH have been shown to interact with RAN1 (Li et al., 2017), providing a link with ethylene signaling (Andrés-Colás et al., 2006; Puig et al., 2007). Chaperones are required for the transport of reactive copper to the correct compartment via these chaperones, avoiding cytotoxicity, after import into the cell; and into the xylem from the cytosol via HMA5, which is structurally similar to RAN1 (Williams and Mills, 2005; Burkhead et al., 2009). The loss-of-function mutants *cch* and *atx1* have no abnormal phenotype when grown under standard conditions, but *atx1* and the *atx1 cch* double mutant, although not the *cch* single mutant, are hypersensitive to exogenous copper (Shin et al., 2012). The functions of ATX1 and CCH have likely diverged, a view supported by their differential transcriptional regulation by copper and different cell-type specificities (Shin et al., 2012). The *ran1* mutant, however, is not copper hypersensitive, whereas *hma5* is; and the *pls* mutant, like *ran1*, is not copper hypersensitive (supplemental Figure 14), suggesting that its role is distinct from that of ATX1 or CCH. Interestingly, however, CCH is downregulated in the *pls* mutant (supplemental Table 8), suggesting some functional interaction between PLS and CCH. However, given its predicted low concentration in the cell and its 2:1 stoichiometry with Cu(I) (Figure 5F and 5G and supplemental Figures 11 and 12), we conclude that PLS is unlikely to act as a cytosolic copper chaperone.

A lack of copper delivery in the strong *ran1-3* and *ran1-4* null alleles causes constitutive ethylene responses (Woeste and Kieber, 2000), producing a triple-response phenotype similar to that of the *pls* mutant (Casson et al., 2002). These *ran1* mutants fail to bind ethylene owing to a lack of copper at the ethylene binding site (Binder et al., 2010). The decrease in ethylene binding in the *ran1-3* and *ran1-4* mutants is not due to reduced levels of ETR1 protein (Binder et al., 2010), and regulation of the ethylene receptor ETR1 is generally regarded to be independent from *ETR1* transcription or degradation (Hua et al., 1998). Weaker *ran1-1* and *ran1-2* alleles show an ethylene response phenotype only if they are grown in the presence of copper chelators (Binder et al., 2010). Therefore, defective RAN1 results in defective copper delivery to wild-type receptors, generating non-functional receptors that cannot interact with CTR1, resulting in downstream ethylene responses.

Intriguingly, the *ran1-3* and *ran1-4* mutants with constitutive ethylene responses are distinct from the ethylene-insensitive *etr1-1* mutant, which also cannot bind copper. Therefore, both the *etr1-1* mutant receptor and the strong *ran1* mutant alleles have reduced ethylene binding ability, but the *etr1-1* mutation, which is a gain-of-function mutation, produces seedling phenotypes very different from those of seedlings carrying the ethylene-hypersensitive wild-type receptors, which simply lack copper because of defective delivery (in *ran1-3* and *ran1-4).* A receptor carrying the *etr1-1* mutation is maintained in its “active” state and constitutively inhibits downstream ethylene responses, whereas in the *ran1* mutants, ETR1 remains in the “inactive” state, promoting ethylene responses (Binder et al., 2010). Those authors therefore proposed that copper has more than one role in the regulation of ETR1 and ethylene signaling: it is crucial for binding ethylene molecules but is also needed for the process of signal transduction by the receptor protein, through an unknown mechanism, to regulate downstream ethylene responses.

We propose that PLS represents a new component of this pathway, providing some tissue specificity to ethylene receptor activity. Given the low probability that PLS acts as a cytosolic copper chaperone, it is more likely to regulate ethylene responses via its demonstrated Cu-dependent ETR1 binding to induce the active state of the receptor, with consequent inhibition of ethylene responses. The RFP:HDEL and roGFP2 co-localization experiments indicated the presence of PLS at the cytosolic side of the ER, in the ER lumen, and binding with the ETR1 transmembrane domain (as well as other compartments; Figure 3). Given that the PLS fusion proteins may not have mimicked native PLS localization perfectly, we cannot exclude the possibility that PLS is present at both the cytosolic and luminal domains of the ER, or indeed in the membrane.

**Figure 6:**
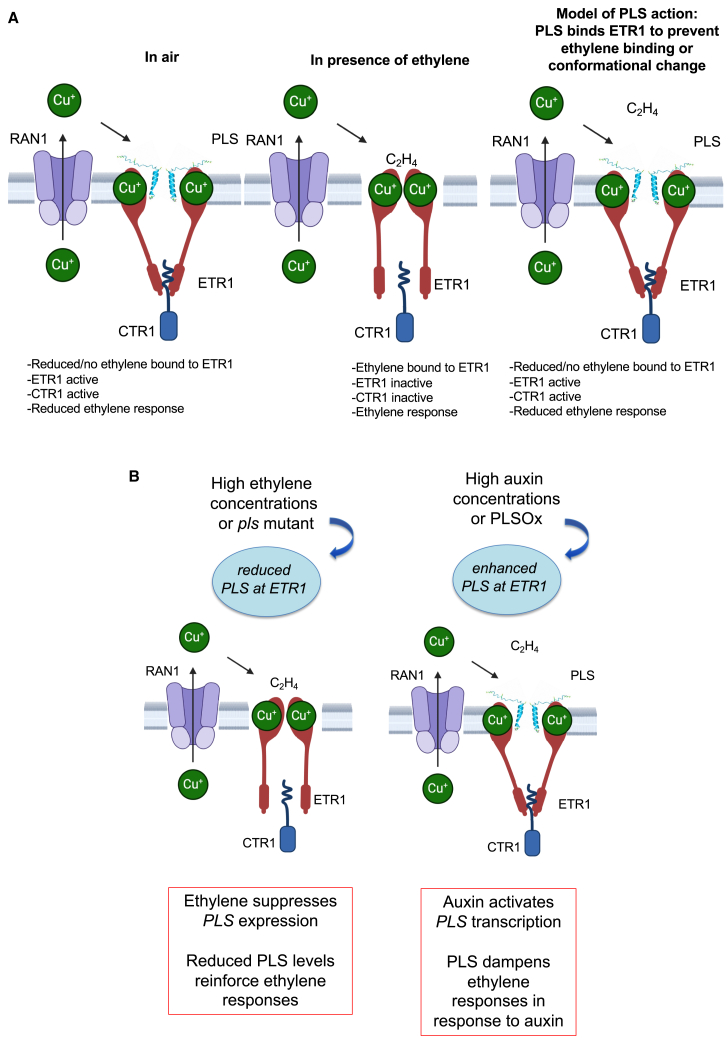
Model of the role of PLS in ethylene signaling. In this model, we assume that RAN1 and ETR1 are in the same membranous compartment (likely the ER), at least transiently. **(A)** Model of PLS interaction with ETR1. RAN1 is believed to pump Cu(I) into the ER lumen and then to ETR1. In air (absence of ethylene; left panel), the receptor complex is active and activates CTR1, a negative regulator of ethylene responses, and PLS likely binds the receptor. In the presence of ethylene (center panel), the receptor is in an inactive state and cannot activate CTR1, resulting in ethylene responses. In the model for PLS function, PLS binds to ETR1 (with binding potentially enhanced by Cu(I) in the ETR1 transmembrane domain) and (as in the left panel) affects the receptor conformation, potentially blocking ethylene binding to ETR1 or inducing some other conformational change, leading to active ETR1, active CTR1, and reduced ethylene signaling. This model is consistent with the enhanced ethylene responses observed in the *pls* loss-of-function mutant. **(B)** Model for the regulation of ethylene responses by auxin and ethylene via effects on *PLS* expression. Left: under high-ethylene conditions or in the *pls* mutant, ETR1 and CTR1 are inactive, leading to enhanced ethylene responses. Right: under high-auxin conditions or in PLS-overexpressing plants, *PLS* transcription is relatively high, and PLS binds to the receptor to disrupt ethylene binding in an otherwise inactive ETR1 receptor (with ethylene responses activated), thus reducing ethylene responses. In, for example, a root tip with relatively high auxin concentrations, *PLS* expression would suppress ethylene responses to allow root growth; but under high-ethylene conditions, root growth would be inhibited (as seen in the *pls* mutant in the absence of high ethylene levels).

The *pls* mutant is rescued by the supply of silver ions (Casson et al., 2002), and silver is likely delivered to the ethylene receptor via RAN1 (Binder et al., 2010); these findings argue against a mechanism in which PLS acts downstream of RAN1 to metalate ETR1 in the ER lumen. It is also unlikely that PLS removes Cu(I) from the receptor, as this would inactivate the receptor and lead to enhanced ethylene signaling in the presence of PLS, which is the opposite of what would be expected from the *pls* mutant phenotype. We therefore favor a model (Figure 6) in which PLS binds ETR1 as part of the receptor biogenesis mechanism, with the PLS–ETR1 physical interaction enhanced in the presence of copper (Figure 4). We propose that this interaction regulates receptor conformation to generate the active form in which ethylene responses are repressed through promotion of receptor interaction with CTR1. This may occur through regulation of Cu(I) homeostasis at the receptor or via other structural interactions; for example, PLS could physically block ethylene binding at the Cu(I) pocket. This question can form the basis of future investigations.

Given that *PLS* is predominantly expressed in specific tissues, notably root tip and leaf vascular tissues (Casson et al., 2002), it seems likely that PLS adds a level of regulation of receptor function in specific tissues, with a focus in this paper on the root (although developmental defects in vascular patterning of the leaf are also observed in the *pls* mutant; Casson et al., 2002). The relatively spatially restricted pattern of *PLS* expression might account for the less strong ethylene hypersignaling phenotype of the *pls* mutant compared with, for example, the strong *ran1* or *ctr1* mutants, although there are similarities, as described above. We propose that PLS acts to dampen ethylene responses at sites of high auxin concentration. *PLS* transcription is strongly upregulated by auxin, and its expression profile is similar to that of the auxin reporter DR5 (Sabatini et al., 1999; Casson et al., 2002). The root tip, where *PLS* is most strongly expressed, is a site of high auxin concentration, whereby an auxin maximum is formed around the stem cell niche to regulate both stem cell identity and function (Sabatini et al., 1999). The vascular tissues are also a site of high auxin concentration (Bishopp et al., 2011). Auxin can induce ethylene biosynthesis by upregulation of the ethylene biosynthetic enzyme ACC synthase (Abel et al., 1995), and high ethylene concentrations can lead to both reduced meristem function and aberrant division of the quiescent center and cells within the stem cell niche and columella (Ortega-Martinez et al., 2007). Therefore, ethylene biosynthesis and signaling in the root tip must be tightly regulated to allow growth control. *PLS* expression is transcriptionally activated by auxin and suppressed by ethylene. We have shown in previous work that there is crosstalk among PLS, ethylene, and other signaling pathways, notably those of auxin, cytokinin, and ABA, in the root (e.g., Liu et al., 2010; 2013; Moore et al., 2015; 2024), and the distinctiveness of *pls* compared with other ethylene signaling mutants may be linked to its restricted expression pattern. This network provides a mechanism to suppress growth-inhibitory ethylene responses in the high-auxin environment of the root tip, which is dependent on the tissue-specific expression of *PLS* (Liu et al., 2010; Figure 6B and supplemental Figure 15) and is mediated through its interaction with the ethylene receptor.

Future structural studies should reveal more about the PLS–ETR1 interaction and the diverse roles of copper ions in ethylene signal transduction, which represents a new paradigm for the regulation of signaling-protein function by metals.

## Methods

### Plant material

*proPLS::PLS:GFP* comprises a 1.5-kb *PLS* promoter sequence (Casson et al., 2002) fused to the *PLS* open reading frame in pMDC107 (obtained from Prof. P.J. Hussey, Durham University) as a C-terminal GFP translational fusion. The *PLS* ORF with its 1.5-kb promoter was amplified from the TOPO 2.1 vector, which contained the *PLS* gene sequence with promoter (Casson et al., 2002); the primers used for cloning are listed in supplemental Table 9. *pls* mutant plants were transformed using the method of Clough and Bent (1998). *proPLS::GFP* plants were generated using the 1.5-kb *PLS* promoter (Casson et al., 2002) fused to *GFP* in a wild-type background. *p35S::GFP* seedlings were obtained from Dr. Piers Hemsley, Durham University. Seedlings of *Arabidopsis thaliana* ecotype C24 or Col-0, *pls* mutants, and transgenic *PLS* overexpressers, all from lab stocks, were grown on solid sterile half-strength (2.2 g/l) Murashige and Skoog medium (Sigma-Aldrich) containing 10 g/l sucrose (half-strength [½] MS10 medium) solidified with 2.5 g/l Phytagel (Sigma-Aldrich) in 90-mm Petri dishes (Sarstedt, Leicester, UK) at 21°C under a 16-h photoperiod as described previously (Casson et al., 2002; Chilley et al., 2006). *N. benthamiana* plants (lab stocks) were grown in a controlled environment (21°C, 16-h photoperiod) for transient expression studies in leaf tissue. For hydroponic feeding studies, *Arabidopsis* seedlings were cultured in liquid ½ MS10 medium (1 ml/well) in sterile 24-well plates (Sarstedt), essentially as described in Matsuzaki et al. (2010). For hormone and peptide assays, one seedling was grown in each well at 21°C under a 16-h photoperiod. For peptide feeding experiments, purified freeze-dried peptide was dissolved in DMSO to create a 500-μM stock solution. The peptide stock solution was added to liquid ½ MS10 medium containing 0.1% DMSO to obtain a final peptide concentration of 50 or 100 nM (or 10, 25, 50, and 100 nM for dose-dependent assays). For copper treatments, 1 mM CuSO_4_ solution was filter sterilized and added to autoclaved liquid ½ MS10 medium to obtain final CuSO_4_ concentrations of 0, 5, 10, 15, 20, 25, 30, 35, 40, 45, and 50 μM. Seedlings were scanned to create a digital image, and root lengths of the seedlings were measured using ImageJ. Statistical analysis was performed using Real Statistics Resource Pack software (release 3.8, www.real-statistics.com) in Excel (Microsoft) using data from at least 10 independently grown seedlings for root growth analysis; specific numbers are defined for each experiment.

### Peptide synthesis

Peptides were either obtained from Cambridge Research Biochemicals (Billingham, UK) or synthesized in the laboratory by Fmoc solid-phase peptide synthesis on a CEM Liberty1 single-channel peptide synthesizer equipped with a Discover microwave unit. Reactions were performed in a 30-ml PTFE reaction vessel with microwave heating FW:EpiLife Media and agitation by bubbling nitrogen. Peptide synthesis was performed using 2-chlorotrityl chloride resin (0.122 mmol g^−1^, Novabiochem) using Fmoc-protected amino acids. The first amino acid residue at the C terminus (histidine) was coupled manually by mixing 76 mg (1 eq.) Fmoc-His(Trt)-OH, 0.09 ml (4 eq.) *N,N*-diisopropylethylamine (DIPEA), 1 ml dichloromethane (DCM), and 1 ml dimethylformamide (DMF) until the amino acid powder had dissolved. The mixture was added to 0.1 mmol resin and stirred gently for 120 min at room temperature. Resin was washed with 3× DCM/MeOH/DIPEA (17:2:1), 3× DCM, 2× DMF, and 2× DCM. Amino acid coupling reactions were performed using Fmoc-protected amino acids present in five-fold excess (2 M concentration), HOBt (0.5 M HOBt in DMF, used at the activator position), and DIC (0.8 M in DMSO, used at the activator base position). For double and triple couplings, the reaction vessel was drained after each coupling cycle, and fresh reagents were added. Before each coupling, a room temperature pre-activation period of 1–2 h was used. Microwave-assisted couplings were performed for 10 min at 75°C at 25 W power. Cys and His residues were coupled at low temperature (10 min at room temperature followed by 10 min at 50°C, 25 W). Arg residues were double coupled, first for 45 min at room temperature plus 5 min at 75°C (25 W) and then with the standard microwave conditions above. Fmoc groups were removed by two piperidine solution treatments (20% piperidine in DMF) in succession: 5 min, then 10 min. The peptide was cleaved from the resin using 3 ml 95% TFA in dH_2_O/TIPS (2.85 ml TFA, 0.15 ml dH_2_O, 0.15 ml triisopropylsilane), then dissolved in water with a small volume of MeCN and lyophilized to produce a powder using a Christ ALPHA 1-2 LDplus freeze dryer. The PLS truncation N1 (Figure 2A) was tagged at the N terminus with a 5-FAM fluorescent tag to enable detection of peptide uptake. The 5-FAM molecule contains a carboxylic acid moiety that can be attached to the N-terminal primary amine and is excited at 488 nm. The remainder of the molecule is planar, with four 6-carbon rings and a chemical formula of C_21_H_12_O_7_. Because of the large size of 5-FAM, it was attached to the shorter PLS(N1) peptide rather than the full-length PLS to avoid the possibility that the latter construct was too large for root uptake.

### Preparative HPLC

Peptide products were analyzed and purified by high-performance liquid chromatography (HPLC) at 280 nm. Freeze-dried peptide sample (25–50 mg) was dissolved in 1 ml 1:1 H_2_O:MeCN and injected onto a Speck and Burke Analytical C18 column (5.0 μm, 10.0 × 250 mm) attached to a PerkinElmer (MA) Series 200 LC Pump and a 785A UV/Vis Detector. Separation was achieved by a gradient elution of 10%–80% solvent B (solvent A = 0.08% TFA in water; solvent B = 0.08% TFA in acetonitrile) over 60 min, followed by 80%–100% B over 10 min, with a flow rate of 2 ml/min. Selected peptide fractions were lyophilized and a mass assigned using MALDI-TOF MS. Peptide sequences were identified by MALDI-TOF MS using an Autoflex II ToF/ToF mass spectrometer (Bruker Daltonik, Germany) equipped with a 337-nm nitrogen laser. MS data were processed using FlexAnalysis 2.0 (Bruker Daltonik).

### Imaging

CSLM images for propidium iodide and GFP were obtained using a Leica SP5 TCS confocal microscope as described previously (Rowe et al., 2016). *proPLS::PLS:GFP*, *proPLS::GFP*, and *pro35S::GFP* seedlings were grown for 7 days on Phytagel ½ MS10 medium before ∼25 mm of the root tip was removed and mounted in dH_2_O prior to imaging. The ER marker *pro35S::RFP:HDEL* (Lee et al., 2013) (provided by Dr. Pengwei Wang, Durham University), and the *trans*-Golgi apparatus marker *proFGC-ST:mCherry* (from Nottingham Arabidopsis Stock Centre, www.arabidopsis.info) were introduced into *proPLS::PLS:GFP* plants by the floral dip method of transformation (Clough and Bent, 1998) using *A. tumefaciens* GV3101. The ER was also visualized using ER Tracker Red (ThermoFisher). Seven-day-old seedlings were stained for 30 min in the dark in liquid ½ MS10 medium containing 1 μM ER Tracker Red. Fluorophores were excited by the following lasers: 405 nm UV (for ethidium bromide), 488 nm 20 mW argon (for all GFP, 5-FAM, and acridine orange experiments), 543 nm 1.2 mW HeNe (for propidium iodide, RFP, and mCherry fluorophores), and 594 nm 2 mW HeNe (for ER Tracker). For standard photomultiplier tubes, a laser power of 21% and a smart gain of 800–1000 mV were used, depending on the intensity of the fluorescence. The HyD detector was used at 70%–120%.

### Ratiometric analysis of roGFP2 fusion proteins

N- and C-terminal roGFP2 fusion proteins of PLS under the control of the CaMV35S promoter were generated by Gateway cloning using the pENTR/D-TOPO Cloning Kit (Invitrogen) as described previously (Hoppen et al., 2019). Infiltration and transient expression of roGFP2 fusions and control proteins were carried out as described in Brach et al. (2009). Image acquisition and data analysis were carried out as described by Hoppen et al. (2019). A minimum of 10 leaf optical sections were imaged and used for ratiometric analysis of the redox sensitive excitation properties of roGFP2.

### RNA isolation, RNA-seq, and RT–qPCR

RNA was extracted from 7-day-old seedlings grown on ½ MS10 medium essentially as described previously (Thompson et al., 2023). The Illumina HiSeq 2500 system was used for RNA-seq of three biological replicate samples; libraries were prepared using the Illumina TruSeq Stranded Total RNA with Ribo-Zero Plant Sample Preparation kit (RS-122-2401) essentially as described in Thompson et al. (2023). Library quality control was carried out again using a TapeStation with D1000 ScreenTape (cat. no. 50675582). RNA-seq data were aligned to the TAIR10 genome sequence (EnsemblPlants, release 58) with the corresponding gtf file using STAR (v 2.7.11a; Dobin et al., 2013) to obtain a read count per gene. Read count data were analyzed using DESeq2 v 1.40.2 (Love et al., 2014) to obtain *P* values, adjusted *P* values, and log_2_ fold changes. DEGs were identified using the criterion of adjusted *P* < 0.05. GO analysis was performed using AgriGO (Tian et al., 2017) singular enrichment analysis. Gene expression heatmaps were generated from log_2_ normalized counts using pheatmap (v 1.0.12, https://cran.r-project.org/web/packages/pheatmap/index.html) with row scaling. RNA-seq data were deposited in GEO (https://www.ncbi.nlm.nih.gov/geo/) with accession number GSE256166 (Mudge et al., 2024).

For RT–qPCR, RNA was extracted from 7-day-old seedlings (3 biological replicates, 20 mg of tissue per replicate) as described previously (Thompson et al., 2023). Samples were checked for the presence of genomic DNA by PCR with the *ACTIN2* primers ACT2 forward and reverse. Primer sequences were determined using Primer-BLAST (https://www.ncbi.nlm.nih.gov/tools/primer-blast/). For each cDNA sample, transcript abundance of a gene of interest was quantified in relation to a stably expressed internal reference “housekeeping” gene, with three technical replicates. This allows the determination of fold-differences in the expression of the gene of interest that can be attributed to the treatment of the sample and corrects for variation between samples, such as differences in the quality or quantity of the extracted RNA. The data were analyzed by comparative quantitation using Rotor-Gene software. Mean transcript abundance was calculated from relative transcript abundance for each biological sample and represented graphically. Error bars show the upper and lower limits of the standard error of the mean. Primers are listed in supplemental Table 9.

### Protein–protein interaction studies

#### Yeast 2-hybrid

The GAL4 2-hybrid phagemid vector system was used to detect protein–protein interactions *in vivo* in yeast, using the reporter genes β-galactosidase *(lacZ)* and histidine (*HIS3*) in the YRG-2 yeast strain, essentially as described previously (Zhong et al., 2008). DNA sequences encoding the target (ETR1) and bait (PLS) were inserted into the pAD-GAL4-2.1 A and pBD-GAL4 Cam phagemid vectors, respectively, and expressed as hybrid proteins. The hybrid proteins were then assayed for protein–protein interaction.

DNA sequences encoding the target and bait proteins were prepared by PCR amplification using primers designed specifically for the target (ETR1) and bait (PLS). Each set of primers contained specific endonuclease recognition sites on the ends corresponding to the restriction sites in the MCS of the pAD-GAL4-2.1 A and pBD-GAL4 Cam phagemid vectors. The DNA constructs for the target (ETR1) and bait (PLS) with specific restriction sites on the ends were then transformed into the TOPO 2.1 vector, and the sequence of the amplified DNA was confirmed by sequencing with M13 forward (CTG GCC GTC GTT TTA C) and M13 reverse (CAG GAA ACA GCT ATG AC) primers. The two vectors, pAD-GAL4-2.1 and pBD-GAL4 Cam, were digested using specific restriction endonucleases and dephosphorylated prior to ligating the insert DNA. The DNA sequences encoding the target (ETR1) and bait (PLS) were then ligated into the same reading frame as the GAL4 AD of the pAD-GAL4-2.1 phagemid vector and the GAL4 BD of the pBD-GAL4 Cam phagemid vector. The primers used to clone ETR1 and PLS are found in supplemental Table 9.

The pGAL4 control plasmid was used alone to verify that induction of the *lacZ* and *HIS3* genes occurred and that the gene products were detectable. The pLamin C control plasmid was used in pairwise combination with the pAD-wild-type control plasmid or the pAD-MUT control plasmid to verify that the *lacZ* and *HIS3* genes were not induced, as the proteins expressed by each of these pairs do not interact *in vivo*.

Control plasmids were transformed into the YRG-2 strain prior to the initial transformation of the bait and target plasmids and used separately or in pairwise combinations for transformation of the YRG-2 yeast strain. Yeast competent cells were co-transformed with the bait and target plasmids by sequential transformation.

#### Gene cloning for Co-IP

To investigate the interaction between the PLS peptide and the ethylene receptor ETR1, two DNA constructs were created by Gateway cloning. The 105-bp *PLS* gene (without the stop codon) was inserted into the pEarlyGate103 (pEG103) destination vector, which contained the *pro35S* promoter and a C-terminal GFP tag, to produce a vector containing *pro35S::PLS:GFP* DNA. The ETR1 cDNA was inserted into the pEarlyGate301 (pEG301) vector to create a *pro35S::ETR1:HA* construct to express an ETR1 protein with a C-terminal HA tag. *pro35S::GFP* was used as a control. The primers used to clone ETR1 and PLS are found in supplemental Table 9.

#### Infiltration into *N. benthamiana*

Constructs were transiently expressed in *N. benthamiana* (tobacco) leaves as described previously (Voinnet et al., 2003). Experiments were replicated up to five times. Competent *Agrobacterium tumefaciens* GV3101 cells were transformed with the desired plasmid containing the gene of interest and injected with a syringe. The plants were approximately 7–10 weeks old; the chosen leaves were healthy and 3–6 cm in length, and 3 to 4 leaves were infiltrated with each construct.

#### Protein extraction and PLS/ETR1 Co-IP

Total protein was extracted from the infiltrated *N. benthamiana* plants 3 days after infiltration for Co-IP experiments to investigate the interaction between PLS and ETR1, essentially as described previously (Srivastava et al., 2020). For competition assays, 5 or 25 nM full-length PLS peptide was also infiltrated in the presence of 50 μM MG-132 (a proteasome inhibitor) 30 min prior to tissue freezing. ChromoTek (Planegg, Germany) anti-GFP beads were used to immunoprecipitate the PLS:GFP protein, and Sigma-Aldrich (St. Louis, MO, USA) anti-HA beads were used for the HA-tagged ETR1.

SDS–PAGE was used to separate protein fragments. The complexed proteins from the pulldown assay were analyzed on 10%–12% acrylamide gels. Membranes were incubated with primary antibody for 2.5 h (GFP, Abcam, Cambridge, UK: rabbit, 1:10 000; HA, Roche, rat, 1:3000; Rubisco large subunit, Agrisera, rabbit, 1:10 000). Excess primary antibody was then removed by washing three times in 2× TBST (150 mM NaCl, 10 mM Tris, 0.1% v/v Tween 20 [pH 7.4]) for 2, 5, and 10 min, and then incubated for 1 h with the ECL peroxidase-labeled anti-rabbit or anti-rat IgG secondary antibody diluted 1:20 000 in TBST. Excess secondary antibody was again removed by washing three times in 1× TBST. To visualize the probed blot, the membrane was incubated with ECL Western Blotting Detection Reagent immediately prior to imaging. The horseradish peroxidase conjugated to the secondary antibody was detected using X-ray film. The experiment was carried out four times with consistent results.

#### Estimation of synthetic PLS concentration

Freeze-dried synthetic PLS peptide (Cambridge Research Biochemicals) was dissolved in DMSO. An aliquot was added to aqueous buffer (10 mM HEPES [pH 7], 20 mM NaCl, 80 mM KCl), and absorbance was recorded at 280 nm. PLS concentration was estimated from the absorbance and the ProtParam estimated extinction coefficient of 2980 M^−1^ cm^−1^. Concurrently, a sample was submitted for quantitative amino acid analysis (Abingdon Health Laboratory Services). From this analysis, a conversion factor of 2.27 was generated, which was applied to the concentrations determined by A_280 nm_.

### ATX1 purification

*E. coli* BL21(DE3) containing pETatx1 was used to overexpress the wild-type *ATX1* gene from *Arabidopsis thaliana* (optimized for expression in *E. coli*, NovoPro Bioscience). Harvested cells were collected and frozen at −20°C overnight, then defrosted and resuspended in 20 mM HEPES (pH 7.0), 10 mM EDTA, 100 mM NaCl, and 10 mM DTT. Cells were sonicated (Bandelin Sonoplus), and the supernatant was separated by size-exclusion chromatography (GE Healthcare, HiLoad 26.600 Superdex 75 pg) using metal-free buffer lacking EDTA. Fractions containing ATX1 were incubated overnight and pooled before transfer to an anaerobic chamber (Belle Technology) via desalting column where the reductant was removed. ATX1 was quantified by a combination of Bradford assay and Ellman’s reagent to ensure the fully reduced state of the protein. Samples were also analyzed for metal content by inductively coupled plasma mass spectrometry (ICP–MS) to ensure >95% apo-ATX1.

### MBP:PLS/mutant peptide purification

A fusion of PLS to MBP was created using the NEBExpress MBP Fusion and Purification System. Two complementary oligonucleotide primers encoding PLS (optimized for expression in *E. coli*) were annealed and inserted into the pMal-c5x plasmid at the *Xmn*I and *Sal*I insertion sites. The three mutants MBP:PLS(C6S), MBP:PLS(C17S), and MBP:PLS(C6S/C17S) were created by site-directed mutagenesis (QuikChange II, Agilent). *E. coli* NEB Express containing the pMal plasmid with the correct MBP:PLS mutant was used to overexpress each protein. Harvested cells were resuspended in 20 mM Tris–HCl (pH 7.4), 200 mM NaCl, and 1 mM EDTA, then frozen at −20°C overnight. Cells were defrosted in cold H_2_O, sonicated, purified by ammonium sulfate precipitation (where MBP-PLS precipitates >60% saturation), separated on an MBP-Trap column (GE Healthcare), and eluted using buffer containing 10 mM maltose. MBP:PLS-containing fractions were pooled and concentrated using a centrifugal concentrator (Corning, Spin-X UF 30 kDa) and buffer exchanged by desalting column into a metal-free buffer of 20 mM HEPES (pH 7.0) and 50 mM NaCl in an anaerobic chamber (Belle Technology). Mutants containing thiols were quantified using Ellman’s assay, and MBP:PLS(C6S/C17S), which lacks all thiols, was quantified using the Bradford assay alone. Samples were also analyzed for metal content by ICP–MS to ensure >95% apo-protein.

### Anaerobic spectroscopic analysis of Cu(I) complexes

All Cu(I) titration experiments were carried out in an anaerobic chamber (Belle Technology) using metal-free CHELEX-treated, degassed buffers. For titration experiments with Cu(I), aqueous CuSO_4_ stock was quantified in advance by ICP–MS and diluted to working concentrations. The reductant NH_2_OH was included at a final concentration of 1 mM to maintain Cu(I) in its reduced state. Proteins were diluted in buffer to the final concentration specified in each titration in air-tight quartz cuvettes (Helma), and after addition of probe to the concentration specified, titrated with CuSO_4_. After each addition, solutions were thoroughly mixed and absorbance spectra recorded using a Lambda 35 UV/Vis spectrophotometer (PerkinElmer). Titration isotherm data were fitted using simulated affinity curves with DynaFit (Kuzmič, 2009).

### Interaction studies of PLS with copper transporter ETR1 by microscale thermophoresis

Fluorescently labeled ETR1 truncation mutants (Milić et al., 2018) were added to a dilution series of synthetic PLS in 50 mM HEPES, 150 mM NaCl, and 0.015% (w/v) FosCholine 16 (pH 7.6) or 50 mM Tris, 300 mM NaCl, and 0.015% (w/v) FosCholine 16 (pH 7.6). Dissociation constants were calculated using GraphPad Prism 5.

### Determination of dissociation constants for the PLS–ETR1 interaction

Full-length ETR1 and its truncation mutants were purified and labeled as described previously (Milić et al., 2018). Synthetic PLS (94 μM) was diluted serially in 50 mM Tris and 300 mM NaCl (pH 7.6). Fluorescently labeled receptor was added at a final concentration of 50 nM. Thermophoretic behavior was measured in premium capillaries at 50% LED and 50% microscale thermophoresis power. In case of a binding event, data were fitted using GraphPad Prism 5.

## Funding

The authors acknowledge financial support from the UK Biotechnology and Biological Sciences Research Council (BB/E006531/1, BBS/B/0773X, and BB/J014516/1 to K.L.; BB/V006002/1 and BB/M011186/1 to N.J.R.) and from the 10.13039/501100001659Deutsche Forschungsgemeinschaft (German Research Foundation) (267205415 – SFB 1208 project B06 to G.G.).

## Acknowledgments

The authors declare no competing interests. We thank Prof. Steven Cobb (Department of Chemistry, Durham University) for advice on peptide synthesis and Dr. Andrew Foster (Departments of Biosciences and Chemistry, Durham University) for preliminary peptide–Cu interaction analysis. K.L. acknowledges the invaluable early work linking POLARIS and ethylene signaling carried out by Dr. Paul Chilley, who sadly passed away far too soon, and dedicates this paper to his memory.

## Author contributions

K.L. and N.J.R. initiated the project. K.L., J.F.T., N.J.R., A.S., and G.G. designed and supervised various aspects of the work. A.J.M., S.M., W.M., B.O.-P., W.S., W.W., C.T., D.R., F.M.H., C.H., and B.U. performed the experiments and prepared the figures. K.L., N.J.R., and G.G. drafted the initial manuscript, and all authors contributed to reviewing and editing the final version.

## Supplemental information

Document S1. Supplemental Figures 1–15 and supplemental Tables 7–9

Supplemental Table 1. Differentially expressed genes between the *pls* mutant and the wild type

Supplemental Table 2. Differentially expressed genes between the *PLS* transgenic overexpressor (PLSOx) and the wild type

Supplemental Table 3. GO terms upregulated in *pls* mutant vs. wild-type plants

Supplemental Table 4. GO terms downregulated in *pls* mutant vs. wild-type plants

Supplemental Table 5. GO terms upregulated in *PLS* transgenic overexpressor (PLSOx) vs. wild-type plants

Supplemental Table 6. GO terms downregulated in *PLS* transgenic overexpressor (PLSOx) vs. wild-type plants

Document S2. Article plus supplemental information

## References

[bib1] Abel S., Nguyen M.D., Chow W., Theologis A. (1995). *ASC4*, a primary indoleacetic acid-responsive gene encoding 1-amino-cyclopropane-1-carboxylate synthase in *Arabidopsis thaliana*. J. Biol. Chem..

[bib2] Andrés-Colás N., Sancenón V., Rodríguez-Navarro S., Mayo S., Thiele D.J., Ecker J.R., Puig S., Peñarrubia L. (2006). The Arabidopsis heavy metal P-type ATPase HMA5 interacts with metallochaperones and functions in copper detoxification of roots. Plant J..

[bib3] Bauer M., Papenbrock J. (2002). Identification and characterization of single-domain thiosulfate sulfurtransferases from *Arabidopsis thaliana*. FEBS Lett..

[bib4] Binder B.M., Rodríguez F.I., Bleecker A.B. (2010). The copper transporter RAN1 is essential for biogenesis of ethylene receptors in Arabidopsis. J. Biol. Chem..

[bib5] Bishopp A., Help H., El-Showk S., Weijers D., Scheres B., Friml J., Benková E., Mähönen A.P., Helariutta Y. (2011). A mutually inhibitory interaction between auxin and cytokinin specifies vascular pattern in roots. Curr. Biol..

[bib6] Brach T., Soyk S., Müller C., Hinz G., Hell R., Brandizzi F., Meyer A.J. (2009). Non-invasive topology of membrane proteins in the secretory pathway. Plant J..

[bib7] Burkhead J.L., Gogolin Reynolds K.A., Abdel-Ghany S.E., Cohu C.M., Pilon M. (2009). Copper homeostasis. New Phytol..

[bib8] Campos M.L., de Souza C.M., de Oliveira K.B.S., Dias S.C., Franco O.L. (2018). The role of antimicrobial peptides in plant immunity. J. Exp. Bot..

[bib9] Capraro J., Sessa F., Magni C., Scarafoni A., Maffioli E., Tedeschi G., Croy R.R.D., Duranti M. (2015). Proteolytic cleavage at twin arginine residues affects structural and functional transitions of Lupin seed 11S storage globulin. PLoS One.

[bib10] Casareno R.L., Waggoner D., Gitlin J.D. (1998). The copper chaperone CCS directly interacts with copper/zinc superoxide dismutase. J. Biol. Chem..

[bib11] Casson S.A., Chilley P.M., Topping J.F., Evans I.M., Souter M.A., Lindsey K. (2002). The *POLARIS* gene of Arabidopsis encodes a predicted peptide required for correct root growth and leaf vascular patterning. Plant Cell.

[bib12] Chang C. (2003). Ethylene signaling: the MAPK module has finally landed. Trends Plant Sci..

[bib13] Chang C., Kwok S.F., Bleecker A.B., Meyerowitz E.M. (1993). Arabidopsis ethylene-response gene *ETR1* - similarity of product to 2-component regulators. Science.

[bib14] Chen G., Li J., Han H., Du R., Wang X. (2022). Physiological and molecular mechanisms of plant responses to copper stress. Int. J. Mol. Sci..

[bib15] Chen Y.F., Randlett M.D., Findell J.L., Schaller G.E. (2002). Localization of the ethylene receptor ETR1 to the endoplasmic reticulum of Arabidopsis. J. Biol. Chem..

[bib16] Chilley P.M., Casson S.A., Tarkowski P., Hawkins N., Wang K.L.C., Hussey P.J., Beale M., Ecker J.R., Sandberg G.K., Lindsey K. (2006). The POLARIS peptide of Arabidopsis regulates auxin transport and root growth via effects on ethylene signaling. Plant Cell.

[bib17] Chu C.C., Lee W.C., Guo W.Y., Pan S.M., Chen L.J., Li H.M., Jinn T.L. (2005). A copper chaperone for superoxide dismutase that confers three types of copper/zinc superoxide dismutase activity in Arabidopsis. Plant Physiol..

[bib18] Clough S.J., Bent A.F. (1998). Floral dip: a simplified method for *Agrobacterium*-mediated transformation of *Arabidopsis thaliana*. Plant J..

[bib19] Dobin A., Davis C.A., Schlesinger F., Drenkow J., Zaleski C., Jha S., Batut P., Chaisson M., Gingeras T.R. (2013). STAR: ultrafast universal RNA-seq aligner. Bioinformatics.

[bib20] Duckert P., Brunak S., Blom N. (2004). Prediction of preprotein convertase cleavage sites. Protein Eng. Des. Sel..

[bib21] Gao Z., Chen Y.-F., Randlett M.D., Zhao X.-C., Findell J.L., Kieber J.J., Schaller G.E. (2003). Localization of the Raf-like kinase CTR1 to the endoplasmic reticulum of Arabidopsis through participation in ethylene receptor signaling complexes. J. Biol. Chem..

[bib22] Grefen C., Staedele K., Ruzicka K., Obrdlik P., Harter K., Horak J. (2008). Subcellular localization and *in vivo* interactions of the *Arabidopsis thaliana* ethylene receptor family members. Mol. Plant.

[bib23] Hall A.E., Findell J.L., Schaller G.E., Sisler E.C., Bleecker A.B. (2000). Ethylene perception by the ERS1 protein in Arabidopsis. Plant Physiol..

[bib24] Hirayama T., Kieber J.J., Hirayama N., Kogan M., Guzman P., Nourizadeh S., Alonso J.M., Dailey W.P., Dancis A., Ecker J.R. (1999). RESPONSIVE-TO-ANTAGONIST1, a Menkes/Wilson disease-related copper transporter, is required for ethylene signaling in Arabidopsis. Cell.

[bib25] Hoppen C., Müller L., Hänsch S., Uzun B., Milić D., Meyer A.J., Weidtkamp-Peters S., Groth G. (2019). Soluble and membrane-bound protein carrier mediate direct copper transport to the ethylene receptor family. Sci. Rep..

[bib26] Hua J., Chang C., Sun Q., Meyerowitz E.M. (1995). Ethylene insensitivity conferred by Arabidopsis *ERS* gene. Science.

[bib27] Hua J., Sakai H., Nourizadeh S., Chen Q.G., Bleecker A.B., Ecker J.R., Meyerowitz E.M. (1998). EIN4 and ERS2 are members of the putative ethylene receptor gene family in Arabidopsis. Plant Cell.

[bib28] Johnson P.R., Ecker J.R. (1998). The ethylene gas signal transduction pathway: A molecular perspective. Annu. Rev. Genet..

[bib29] Kim J.S., Jeon B.W., Kim J. (2021). Signaling peptides regulating abiotic stresses in plants. Front. Plant Sci..

[bib30] Kuzmič P. (2009). DynaFit—a software package for enzymology. Methods Enzymol..

[bib31] Lee H., Sparkes I., Gattolin S., Dzimitrowicz N., Roberts L.M., Hawes C., Frigerio L. (2013). An Arabidopsis reticulon and the atlastin homologue *RHD3-like2* act together in shaping the tubular endoplasmic reticulum. New Phytol..

[bib32] Li W., Lacey R.F., Ye Y., Lu J., Yeh K.-C., Xiao Y., Li L., Wen C.-K., Binder B.M., Zhao Y. (2017). Triplin, a small molecule, reveals copper ion transport in ethylene signaling from ATX1 to RAN1. PLoS Genet..

[bib33] Liu J., Mehdi S., Topping J., Tarkowski P., Lindsey K. (2010). Modelling and experimental analysis of hormonal crosstalk in Arabidopsis. Mol. Syst. Biol..

[bib34] Liu J., Mehdi S., Topping J., Friml J., Lindsey K. (2013). Interaction of PLS and PIN and hormonal crosstalk in Arabidopsis root development. Front. Plant Sci..

[bib35] Love M.I., Huber W., Anders S. (2014). Moderated estimation of fold change and dispersion for RNA-seq data with DESeq2. Genome Biol..

[bib36] Ma Z., Hu L., Jiang W. (2024). Understanding AP2/ERF transcription factor responses and tolerance to various abiotic stresses in plants: A comprehensive review. Int. J. Mol. Sci..

[bib37] Matsuzaki Y., Ogawa-Ohnishi M., Mori A., Matsubayashi Y. (2010). Secreted peptide signals required for maintenance of root stem cell niche in Arabidopsis. Science.

[bib38] McDaniel B.K., Binder B.M. (2012). ETHYLENE RECEPTOR 1 (ETR1) is sufficient and has the predominant role in mediating inhibition of ethylene responses by silver in *Arabidopsis thaliana*. J. Biol. Chem..

[bib39] McDonald E.F., Jones T., Plate L., Meiler J., Gulsevin A. (2023). Benchmarking AlphaFold2 on peptide structure prediction. Structure.

[bib40] Milić D., Dick M., Mulnaes D., Pfleger C., Kinnen A., Gohlke H., Groth G. (2018). Recognition motif and mechanism of ripening inhibitory peptides in plant hormone receptor ETR1. Sci. Rep..

[bib41] Moore S., Jervis G., Topping J.F., Chen C., Liu J., Lindsey K. (2024). A *predictive model for ethylene-mediated auxin and cytokinin patterning* in the *Arabidopsis* root. Plant Comms.

[bib42] Moore S., Zhang X., Mudge A., Rowe J.H., Topping J.F., Liu J., Lindsey K. (2015). Spatiotemporal modelling of hormonal crosstalk explains the level and patterning of hormones and gene expression in *Arabidopsis thaliana* wildtype and mutant roots. New Phytol..

[bib43] Morgan M.T., Bourassa D., Harankhedkar S., McCallum A.M., Zlatic S.A., Calvo J.S.,, Meloni G., Faundez V., Fahrni C.J. (2019). Ratiometric two-photon microscopy reveals attomolar copper buffering in normal and Menkes mutant cells. Proc. Natl. Acad. Sci. USA.

[bib44] **Mudge, A.J., Mehdi, S., Michaels, W., Orosa-Puente, B., Shen, W., Tomlinson, C., Wei, W., Hoppen, C., Uzun, B., Roy, D., et al.** (2024). Data from: POLARIS is a copper-binding peptide required for ethylene signalling control in *Arabidopsis*. GEO accession GSE256166: https://www.ncbi.nlm.nih.gov/geo/.10.1016/j.xplc.2025.10143240574333

[bib45] Olsson V., Joos L., Zhu S., Gevaert K., Butenko M.A., De Smet I. (2019). Look closely, the beautiful may be small: Precursor-derived peptides in plants. Annu. Rev. Plant Biol..

[bib46] Ortega-Martinez O., Pernas M., Carol R.J., Dolan L. (2007). Ethylene modulates stem cell division in the *Arabidopsis thaliana* root. Science.

[bib47] Puig S., Mira H., Dorcey E., Sancenón V., Andrés-Colás N., Garcia-Molina A., Burkhead J.L., Gogolin K.A., Abdel-Ghany S.E., Thiele D.J. (2007). Higher plants possess two different types of ATX1-like copper chaperones. Biochem. Biophys. Res. Commun..

[bib48] Qu X., Hall B.P., Gao Z., Schaller G.E. (2007). A strong constitutive ethylene-response phenotype conferred on Arabidopsis plants containing null mutations in the ethylene receptors *ETR1* and *ERS1*. BMC Plant Biol..

[bib49] Robinson N.J., Winge D.R. (2010). Copper metallochaperones. Annu. Rev. Biochem..

[bib50] Rodriguez F.I., Esch J.J., Hall A.E., Binder B.M., Schaller G.E., Bleecker A.B. (1999). A copper cofactor for the ethylene receptor ETR1 from Arabidopsis. Science.

[bib51] Rowe J.H., Topping J.F., Liu J., Lindsey K. (2016). Abscisic acid regulates root growth under osmotic stress conditions via an interacting hormonal network with cytokinin, ethylene and auxin. New Phytol..

[bib52] Sabatini S., Beis D., Wolkenfelt H., Murfett J., Guilfoyle T., Malamy J., Benfey P., Leyser O., Bechtold N., Weisbeek P. (1999). An auxin-dependent distal organiser of pattern and polarity in the Arabidopsis root. Cell.

[bib53] Sakai H., Hua J., Chen Q.G., Chang C., Medrano L.J., Bleecker A.B., Meyerowitz E.M. (1998). *ETR2* is an *ETR1*-like gene involved in ethylene signaling in *Arabidopsis*. Proc. Natl. Acad. Sci. USA.

[bib54] Schaller G.E., Ladd A.N., Lanahan M.B., Spanbauer J.M., Bleecker A.B. (1995). The ethylene response mediator ETR1 from Arabidopsis forms a disulfide-linked dimer. J. Biol. Chem..

[bib55] Schott-Verdugo S., Müller L., Classen E., Gohlke H., Groth G. (2019). Structural model of the ETR1 ethylene receptor transmembrane sensor domain. Sci. Rep..

[bib56] Shin L.-J., Lo J.-C., Yeh K.-C. (2012). Copper chaperone antioxidant protein1 is essential for copper homeostasis. Plant Physiol..

[bib57] Srivastava M., Srivastava A.K., Orosa-Puente B., Campanaro A., Zhang C., Sadanandom A. (2020). SUMO conjugation to BZR1 enables brassinosteroid signaling to integrate environmental cues to shape plant growth. Curr. Biol..

[bib58] Thompson H.L., Shen W., Matus R., Kakkar M., Jones C., Dolan D., Grellscheid S., Yang X., Zhang N., Mozaffari-Jovin S. (2023). MERISTEM-DEFECTIVE regulates the balance between stemness and differentiation in the root meristem through RNA splicing control. Development.

[bib59] Tian T., Liu Y., Yan H., You Q., Yi X., Du Z., Xu W., Su Z. (2017). agriGO v2.0: a GO analysis toolkit from the agricultural community, 2017 update. Nucleic Acids Res..

[bib60] Yu C.H., Yang N., Bothe J., Tonelli M., Nokhrin S., Dolgova N.V., Braiterman L., Lutsenko S., Dmitriev O.Y. (2017). The metal chaperone Atox1 regulates the activity of the human copper transporter ATP7B by modulating domain dynamics. J. Biol. Chem..

[bib61] Voinnet O., Rivas S., Mestre P., Baulcombe D. (2003). An enhanced transient expression system in plants based on suppression of gene silencing by the p19 protein of tomato bushy stunt virus. Plant J..

[bib62] Williams L.E., Mills R.F. (2005). P(1B)-ATPases: an ancient family of transition metal pumps with diverse functions in plants. Trends Plant Sci..

[bib63] Willoughby A.C., Nimchuk Z.L. (2021). WOX going on: CLE peptides in plant development. Curr. Opin. Plant Biol..

[bib64] Woeste K.E., Kieber J.J. (2000). A strong loss-of-function mutation in RAN1 results in constitutive activation of the ethylene response pathway as well as a rosette-lethal phenotype. Plant Cell.

[bib65] Xiao Z., Brose J., Schimo S., Ackland S.M., La Fontaine S., Wedd A.G. (2011). Unification of the copper(I) binding affinities of the metallo-chaperones Atx1, Atox1, and related proteins: detection probes and affinity standards. J. Biol. Chem..

[bib66] Yamasaki H., Hayashi M., Fukazawa M., Kobayashi Y., Shikanai T. (2009). SQUAMOSA promoter binding protein-like7 is a central regulator for copper homeostasis in *Arabidopsis*. Plant Cell.

[bib67] Yang Y., Hao C., Du J., Xu L., Guo Z., Li D., Cai H., Guo H., Li L. (2022). The carboxy terminal transmembrane domain of SPL7 mediates interaction with RAN1 at the endoplasmic reticulum to regulate ethylene signalling in Arabidopsis. New Phytol..

[bib68] Zhang H., Li L. (2013). SQUAMOSA promoter binding protein-like7 regulated microRNA408 is required for vegetative development in *Arabidopsis*. Plant J..

[bib69] Zhang H., Zhao X., Li J., Cai H.,, Deng X.W., Li L. (2014). MicroRNA408 is critical for the HY5-SPL7 gene network that mediates the coordinated response to light and copper. Plant Cell.

[bib70] Zhong S., Lin Z., Grierson D. (2008). Tomato ethylene receptor-CTR interactions: visualization of NEVER-RIPE interactions with multiple CTRs at the endoplasmic reticulum. J. Exp. Bot..

