# Supplementary material for: POLARIS is a copper-binding peptide that interacts with ETR1 to negatively regulate ethylene signaling in Arabidopsis
Source: Plant Commun. 2025 Jun 25;6(12):101432. doi: 10.1016/j.xplc.2025.101432 (PMC12744750; doi:10.1016/j.xplc.2025.101432)
Supplement: Document S1. Supplemental Figures 1–15 and supplemental Tables 7–9 [file mmc1.pdf]

Plant Communications, Volume *6*

Supplemental information

POLARIS is a copper-binding peptide that interacts with ETR1 to negatively regulate ethylene signaling in *Arabidopsis*

Anna J. Mudge, Saher Mehdi, Will Michaels, Beatriz Orosa-Puente, Weiran Shen, Charlie Tomlinson, Wenbin Wei, Claudia Hoppen, Buket Uzun, Dipan Roy, Flora M. Hetherington, Jennifer F. Topping, Ari Sadanandom, Georg Groth, Nigel J. Robinson, and Keith Lindsey

Supplementary Text, Figures and Tables for:

POLARIS is a copper-binding peptide that interacts with ETR1 to negatively regulate ethylene signaling in *Arabidopsis*

Anna J. Mudge[1], Saher Mehdi[1], Will Michaels[1,2], Beatriz Orosa-Puente[1], Weiran Shen[1], Charlie Tomlinson[2], Wenbin Wei[1], Claudia Hoppen[3], Buket Uzun[3], Dipan Roy[1], Flora M. Hetherington[1], Jennifer F. Topping[1], Ari Sadanandom[1], Georg Groth[3], Nigel J. Robinson[1,2], Keith Lindsey[1]

Affiliations

[1]*Department of Biosciences, Durham University, Durham DH1 3LE, UK*

[2]*Department of Chemistry, Durham University, Durham DH1 3LE, UK*

[3]*Institute of Biochemical Plant Physiology, Heinrich Heine University Düsseldorf, D-40204 Düsseldorf, Germany*

Supplementary text

Derivation of equations used to describe the metalation of a protein (P) that forms a 2:1 complex (MP_2) with a metal (M).

Cu(I) occupancy of the cytosolic copper chaperone ATX1 is tuned to track with fluctuations in available cytosolic Cu(I) such that its affinity provides a first approximation of Cu(I) availability within this compartment. Based on the determined Cu(I) stoichiometry of *Arabidopsis* ATX1 (Fig. 5H) and subsequent affinity (Fig. S12), Cu(I) occupancy of a 1:2 Cu(I):PLS_2 complex has been calculated as a function of total cytosolic [PLS].

The following equilibrium describes the formation of a 1:2 complex of metal (M) and protein (P)

$$M + 2P \leftrightharpoons MP_2$$

The equilibrium constant for the association of the reaction is

$$\beta_2 = \frac{[MP_2]}{[M][P]^2} \qquad (1)$$

The fractional occupancy of the protein with the metal M can be defined as:

$$Fractional\ occupancy\ (\%) = 100 \text{ x } \frac{2[MP_2]}{[P]_{tot}} \qquad (2)$$

The following is the mass balance equation for the protein

$$[P]_{tot} = [P] + 2[MP_2] \qquad (3)$$

Equations *(2)* and *(3)* can be combined to give

$$\frac{2[MP_2]}{[P]_{tot}} = \frac{2[MP_2]}{[P]+2[MP_2]} \qquad (4)$$

Substituting *(1)* into *(4)* gives

$$\frac{2[MP_2]}{[P]_{tot}} = \frac{2\beta_2[M][P]^2}{[P]+2\beta_2[M][P]^2} \qquad (5)$$

Simplifying leaves the fractional occupancy in terms of metal affinities (β_2), available metal concentration ([M]), and concentration of *apo*- (uncomplexed) protein ([P]).

$$\frac{2[MP_2]}{[P]_{tot}} = \frac{2\beta_2[M]}{[P]^{-1}+2\beta_2[M]} \qquad (6)$$

The *apo*-protein concentration is itself a function of metal affinities, total protein concentration and available metal concentration. Substituting *(1)* into *(3)* and rearranging gives

$$[P]_{tot} = [P] + 2\beta_2[M][P]^2$$

$$So: 2\beta_2[M][P]^2 + [P] - [P]_{tot} = 0 \qquad (7)$$

Can solve this equation for [P] using the quadratic formula:

$$[P] = \frac{-b \pm \sqrt{b^2 - 4ac}}{2a} \tag{8}$$

Where: $a = 2\beta_2[M],\ b = 1\ and\ c = -[P]_{tot}$

The fractional occupancy of the protein with metal can therefore be expressed as a function of metal affinity, metal availability and total protein concentration (*ie* measurable parameters) by combining equations (*6*) and (*8*)

$$\frac{2[MP_2]}{[P]_{tot}} = \frac{2\beta_2[M]}{\frac{-b \pm \sqrt{b^2 - 4ac}}{2a}^{-1} + 2\beta_2[M]} \tag{9}$$

Where: $a = 2\beta_2[M],\ b = 1\ and\ c = -[P]_{tot}$

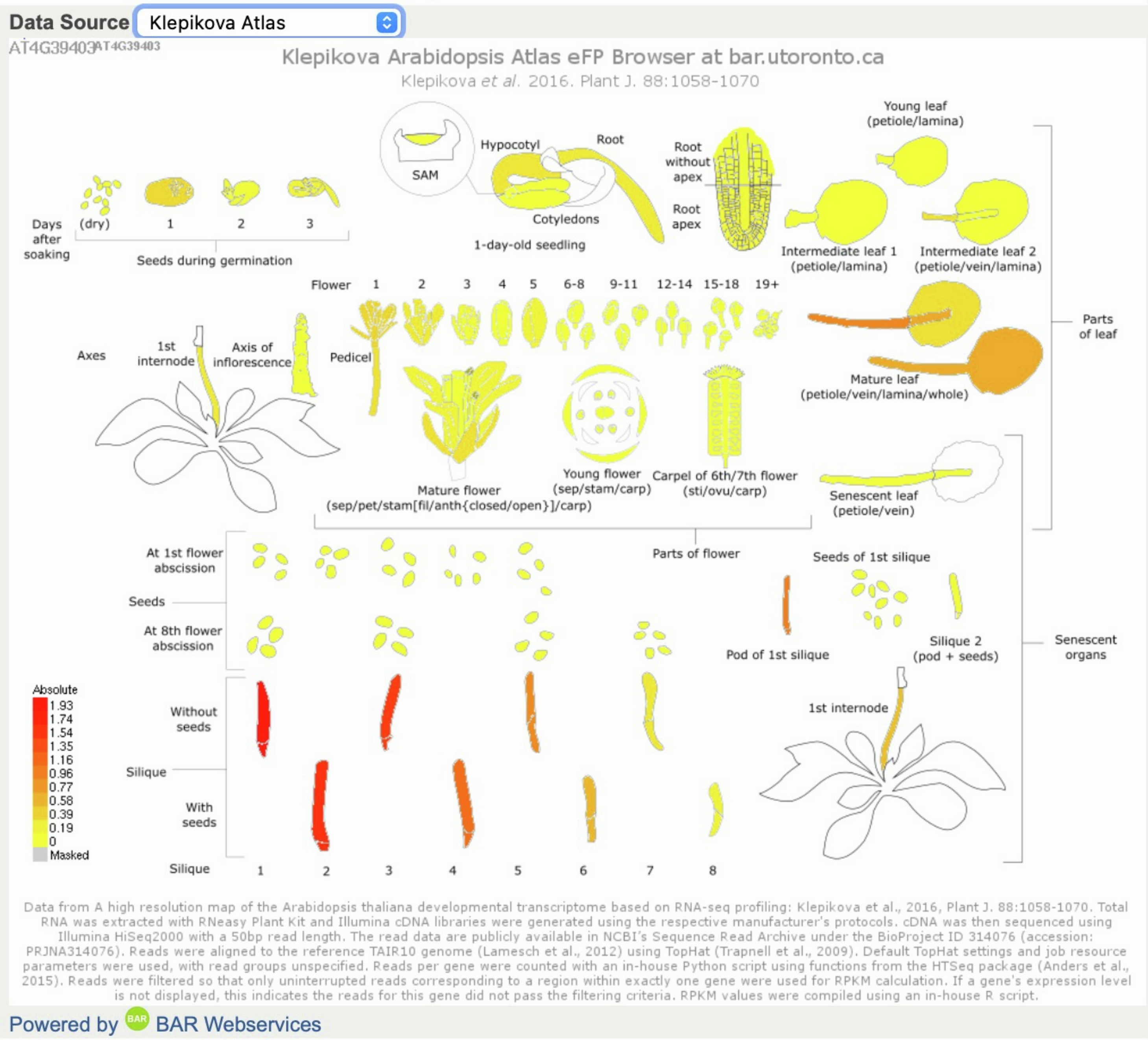

Figure S1. *POLARIS* (AT4G39403) expression.
A high resolution map of the *Arabidopsis thaliana* developmental transcriptome based on RNA-seq profiling: Klepikova et al., 2016, Plant J. 88:1058-1070.

From https://www.arabidopsis.org/servlets/TairObject?id=1001029664&type=locus

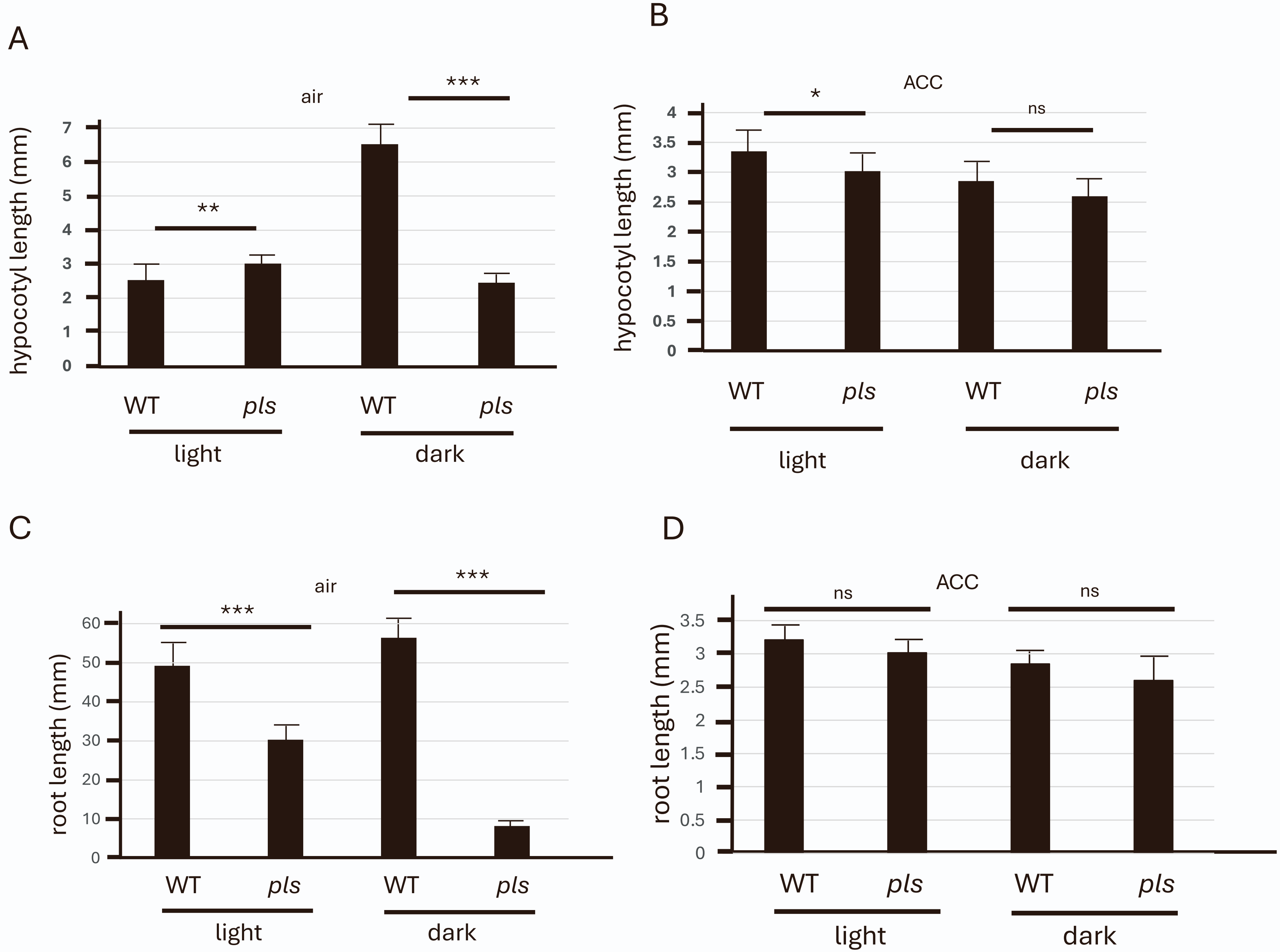
A
air
hypocotyl length (mm)
0
1
2
3
4
5
6
7
**
***
WT
pls
WT
pls
light
dark
B
ACC
hypocotyl length (mm)
0
0.5
1
1.5
2
2.5
3
3.5
4
*
ns
WT
pls
WT
pls
light
dark
C
air
root length (mm)
0
10
20
30
40
50
60
***
***
WT
pls
WT
pls
light
dark
D
ACC
root length (mm)
0
0.5
1
1.5
2
2.5
3
3.5
ns
ns
WT
pls
WT
pls
light
dark

Figure S2. Hypocotyl and root length measurements of 10 d old wild type and *pls* mutant seedlings grown in light or dark, in air or in the presence of 10 μ M ACC. Results represent means ± SD, n = 12. Significant differences determined by Student's t test: ns - no significant difference, * = P value <0.05, ** - P value < 0.01, *** = P value < 0.005.

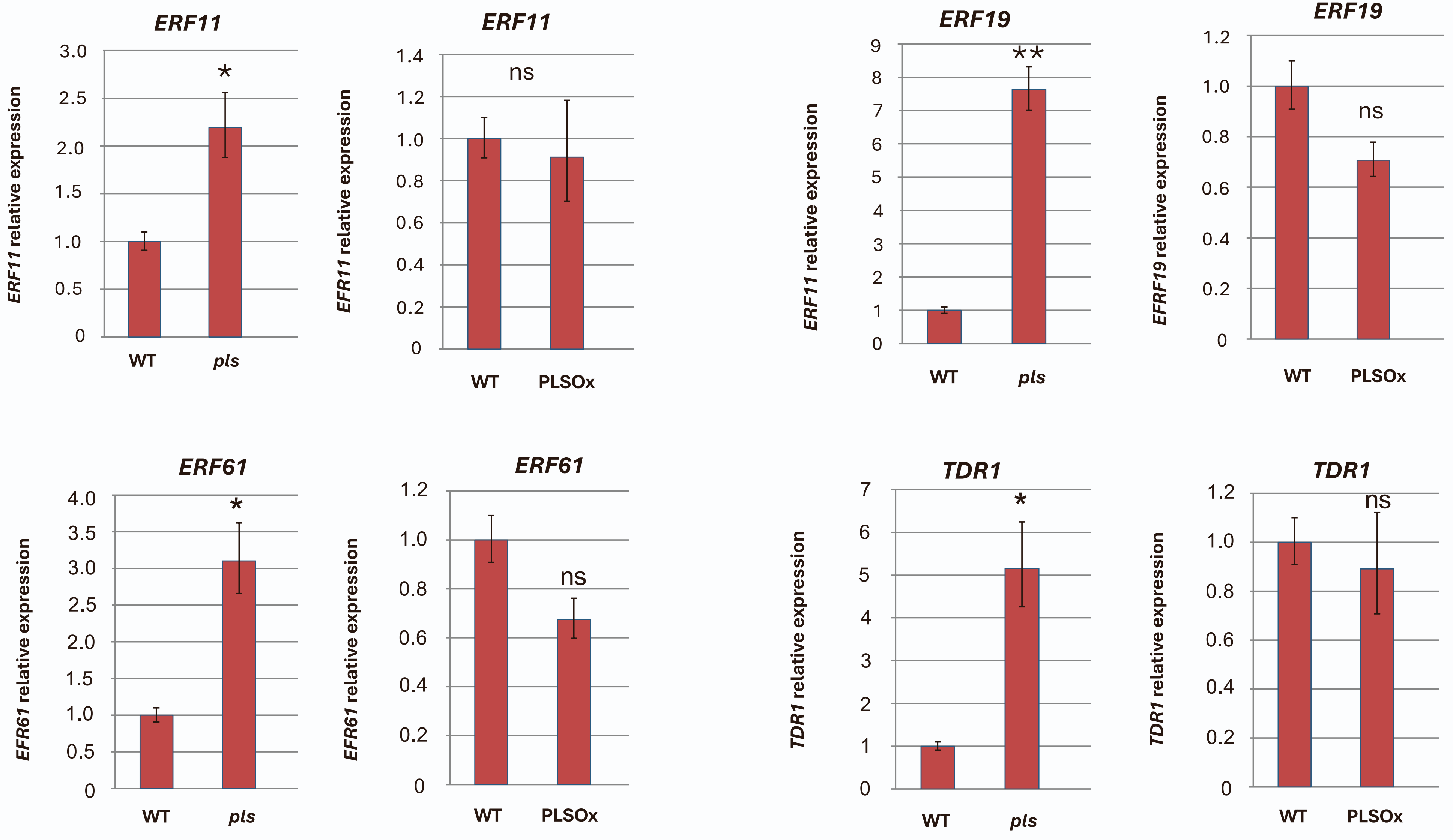

Figure S3. Validation RT-qPCR analysis of genes found to be up-regulated in the *pls* mutant by RNA-seq (Table S1). Values represents means and error bars are SEM (n = 3 biological repeats with three technical repeats) for both 7 day-old total seedlings of *pls* mutants and transgenic PLS overexpressers (PLSOx). *ACTIN1* was used as the reference gene and mutant data are compared to wild type which is normalised as 1. Statistical significance was determined using Student's t-test for independent samples compared to wild type values, with P-values <0.05 (*), P <0.01 (**).

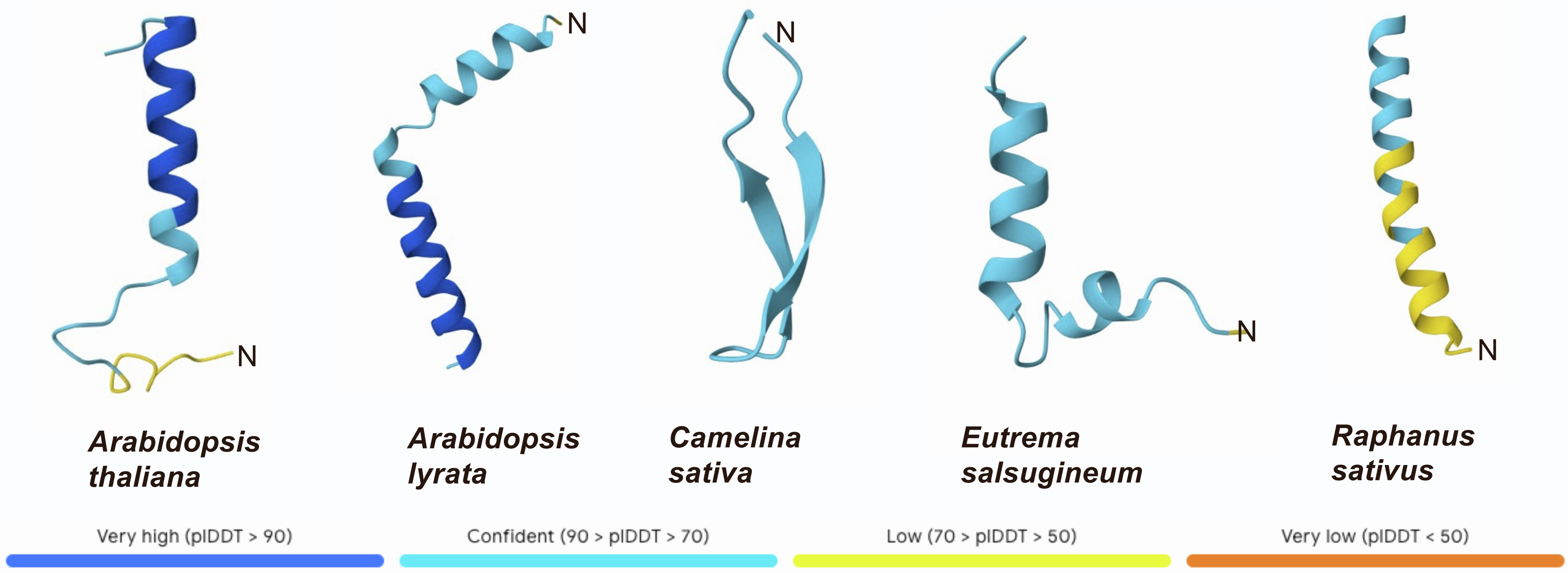

Figure S4. Predicted structures of POLARIS-like peptides from Arabidopsis and four relatives, generated using AlphaFold2. The 36 amino acid POLARIS sequence was used as a BLAST search term. The results show significant alignments in these species but limited structural conservation.

A

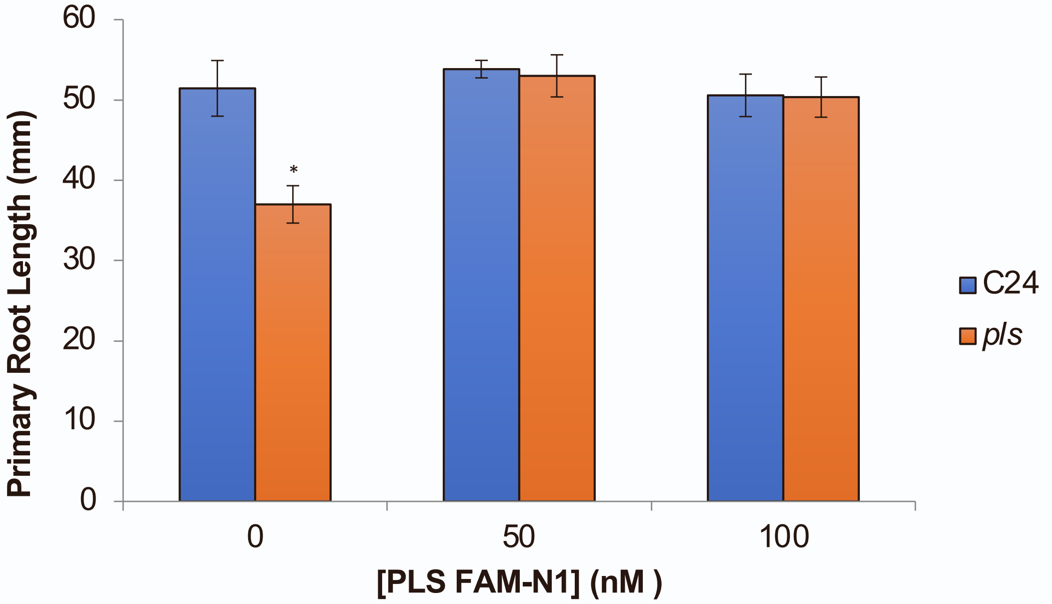

B

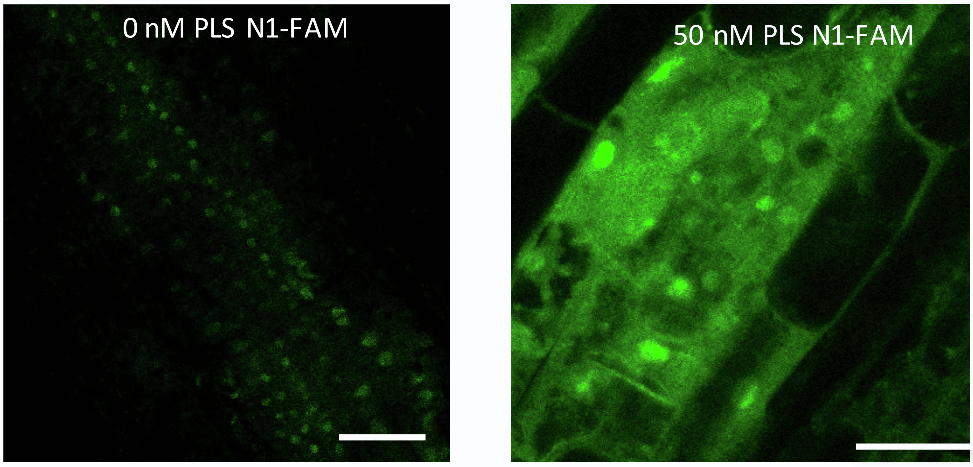

Figure S5. Synthetic PLS peptide is taken up and rescues root development in *pls* seedlings.

(**A**) Effect of fluorescently tagged (5-FAM) synthetic PLS (N1) peptide on primary root length of *Arabidopsis*. Wildtype (C24, blue bars) and *pls* mutant (red bars) seedlings were grown hydroponically in the presence of 0, 50 or 100 nM peptide for 10 days. *: $P < 0.05$, *t*-test between C24 and *pls*. Bars show ± standard errors of the mean, n = 15.

(**B**) 5-FAM-PLS(N1) is taken up by *Arabidopsis* roots. Confocal images of wildtype *Arabidopsis* roots (elongation zone) grown hydroponically for 10 days in the absence of peptide (left panel) and in the presence of 50 nM peptide (right panel). Images are representative of 8 independent root tips imaged. Scale bars = 10 µm.

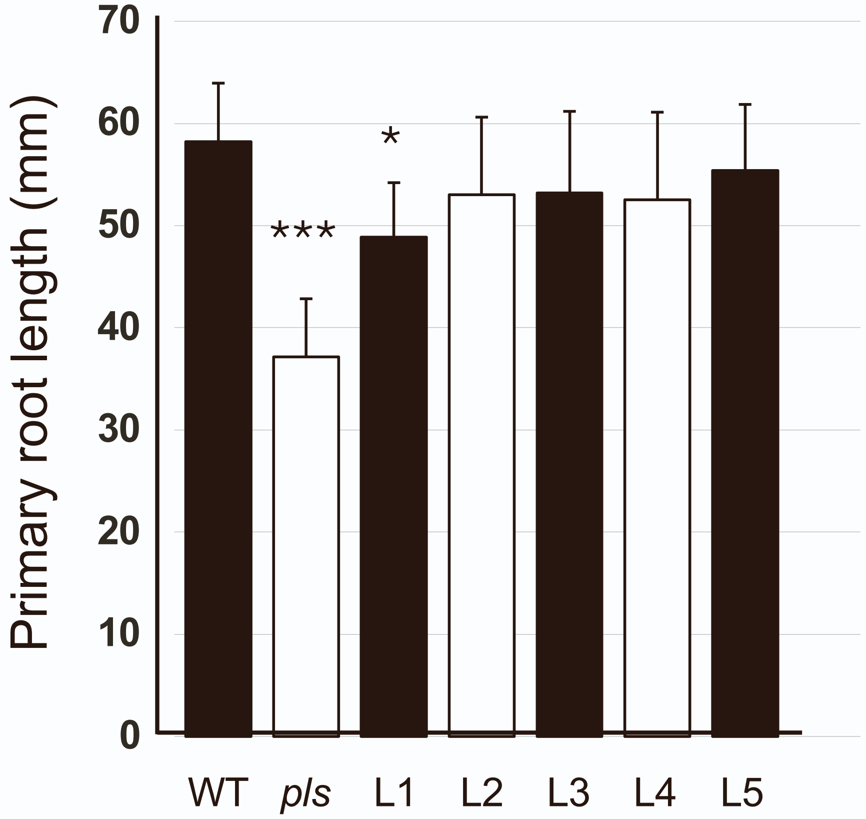

Figure S6. The *proPLS::PLS:GFP* gene fusion complements the *pls* mutant.

Primary root length of seedlings of wild type (WT), *pls* mutant (*pls*), and five independent transgenic lines (L1-L5) expressing *pro::PLS:PLS:GFP* in the *pls* mutant background. Seedlings were grown for 10 days on solid half MS10 agar medium. ***: *P* <0.0001, * : *P* <0.01 *t*-test between WT (C24) and *pls* or transgenic line. Bars represent +1 SD, n = 13. No significant difference detected between WT and Lines 2-5.

A

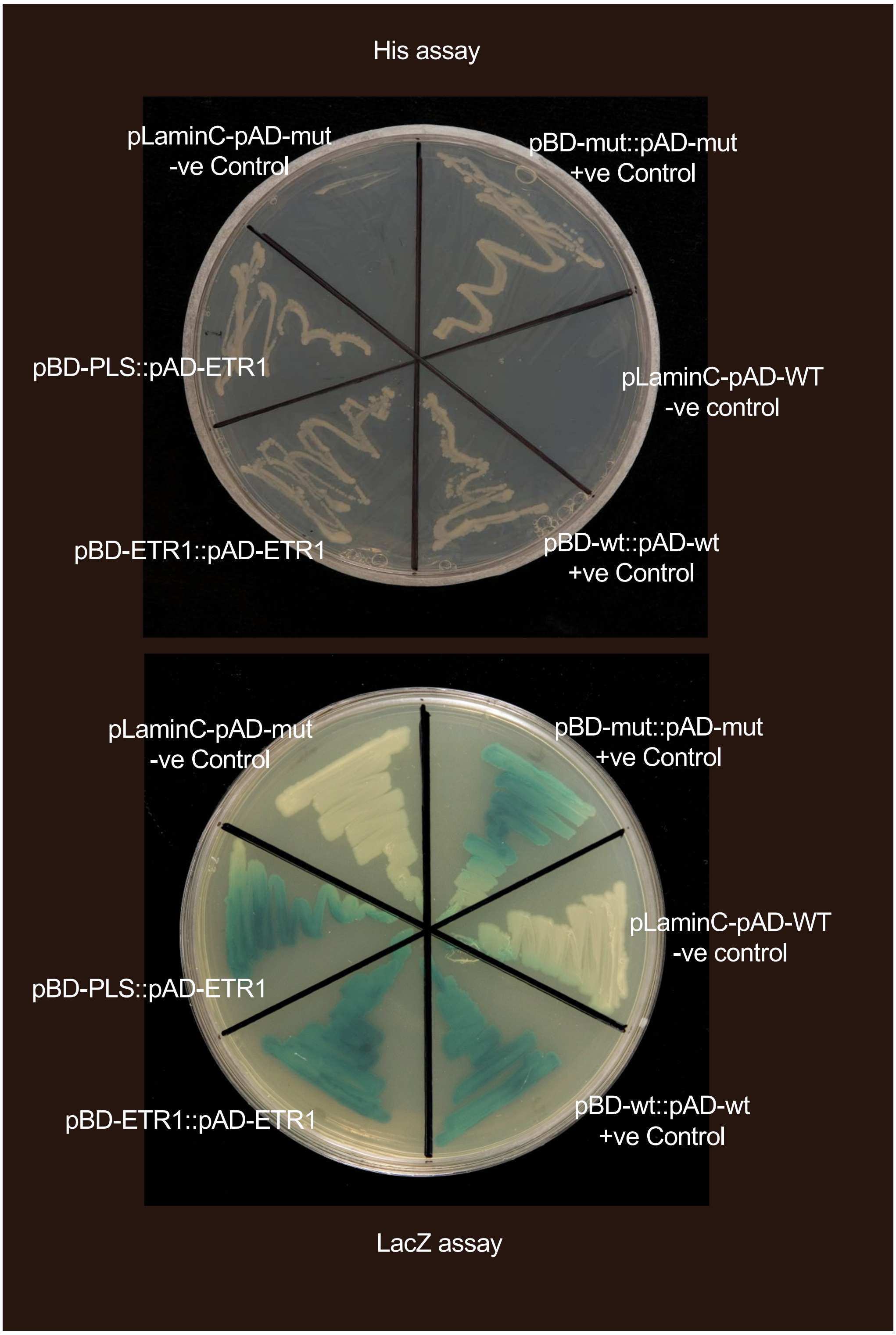
His assay
pLaminC-pAD-mut
-ve Control
pBD-mut::pAD-mut
+ve Control
pBD-PLS::pAD-ETR1
pLaminC-pAD-WT
-ve control
pBD-ETR1::pAD-ETR1
pBD-wt::pAD-wt
+ve Control
pLaminC-pAD-mut
-ve Control
pBD-mut::pAD-mut
+ve Control
pLaminC-pAD-WT
-ve control
pBD-PLS::pAD-ETR1
pBD-ETR1::pAD-ETR1
pBD-wt::pAD-wt
+ve Control
LacZ assay

B

The GAL4 two-hybrid vector system contains six control plasmids listed below:

Control plasmid	Insert description	Vector	Genotype	Function
pGAL4	Wild-type, full length GAL4	pRS415	LEU2, Amp^r	Positive control
pBD-WT	Wild-type fragment C of lamda cl repressor (aa 132-236)	pAD-GAL4 Cam	TRP1, Cam^r	Interaction control
pAD-WT	Wild-type fragment C of lamda cl repressor (aa 132-236)	pBD-GAL4 2.1	LEU2, Amp^r	Interaction control
pBD-MUT	E233K mutant fragment of lamda cl repressor (aa 132-236)	pBD-GAL4 Cam	TRP1, Cam^r	Interaction control
pAD-MUT	E233k mutant fragment of lamda cl repressor (aa 132-236)	pAD-GAL4 2.1	LEU2, Amp^r	Interaction control
pLaminC	Human Lamin C (aa 67-230)	pBD-GAL4	TRP1, Amp^r	Negative control

Figure S7. PLS interacts with ETR1 in yeast.
(A) Yeast 2-hybrid assays showing and ETR1 interacts with PLS and with ETR1, in both His3 (upper plate) and LacZ (lower plate) reporter assays. Positive controls (pBD-wt::pAD-wt and pBD-mut::pAD-mut) and negative controls (pLaminC::pAD-mut and pLaminC::PAD-wt; pBD-PLS::pAD-ETR1 and pBD-ETR1::pAD-ETR1) are shown for each. The control plasmids are described in the Table (B).

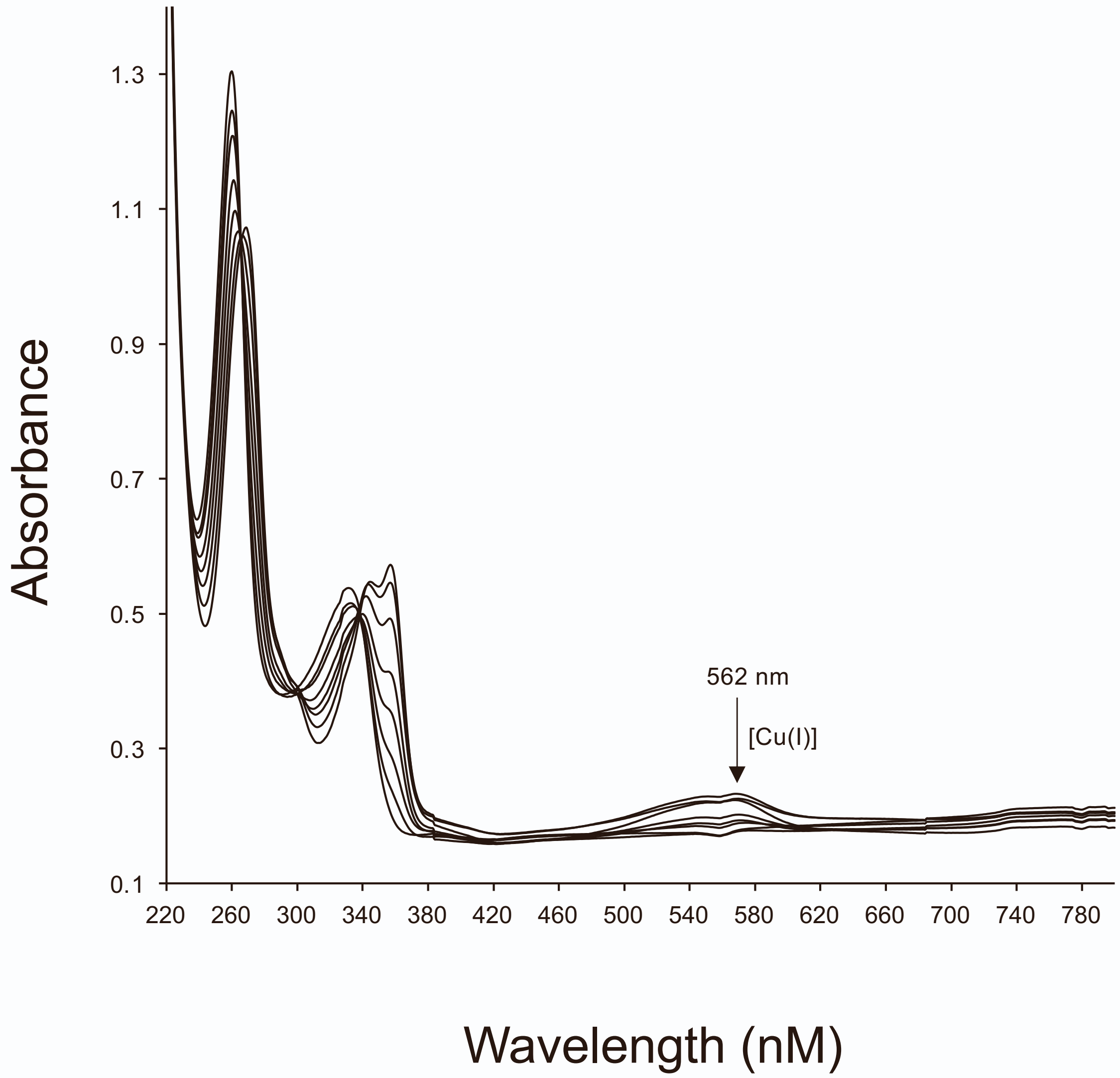

Figure S8. Full data representation from UV-vis spectra shown in Fig. 5D.
Titration of BCA with Cu(I) as in Figure 5D, showing additional copper-dependent features at 562 nm.

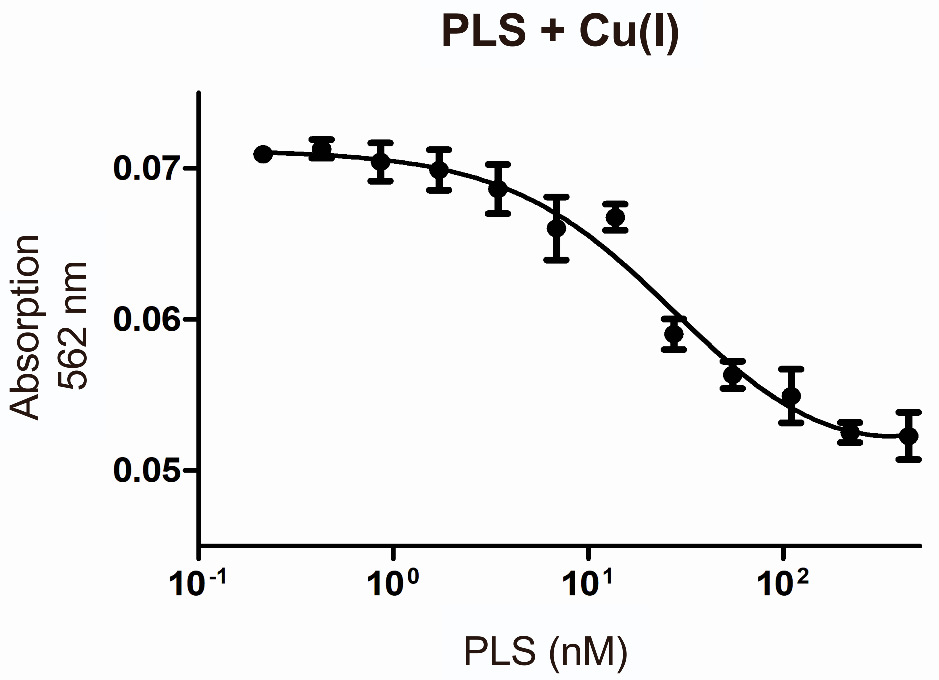

Figure S9. Titration of PLS in the presence of BCA_2-Cu(I)-Complex.
A solution of synthetic PLS in 50 µl buffer (50 mM HEPES, 150 mM NaCl, pH 7.6) was serially diluted from 496 µM to 214 nM. After the addition of 50 µl BCA_2-Cu(I) buffer (50 mM HEPES, 150 mM NaCl, 20 mM ascorbate, 125 µM BCA, 50 µM CuCl, pH 7.6) the absorbance at 562 nm was measured. Discoloration of the pink BCA_2-Cu(I) complex visible in the decrease of absorption at higher peptide concentration indicates that PLS is able to effectively compete and remove copper from the high affinity copper chelator BCA. Bars represent means ± SD, n = 3.

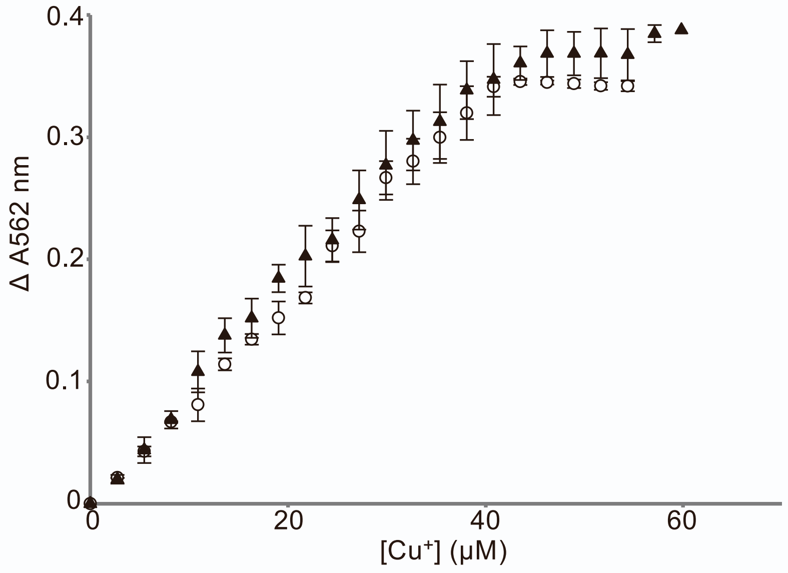

Figure S10. PLS requires C6S and C17S cysteine residues for copper binding.

BCA absorbance in the presence of synthetic PLS FL C6S, C17S ([BCA] = 93.3 μM, [PLS FL C6S, C17S] = 40 μM, closed triangles) or an equivalent volume (to PLS FL C6S, C17S) of DMSO ([BCA] = 87.4, open circles). Values represent means ± SD, n = 3.

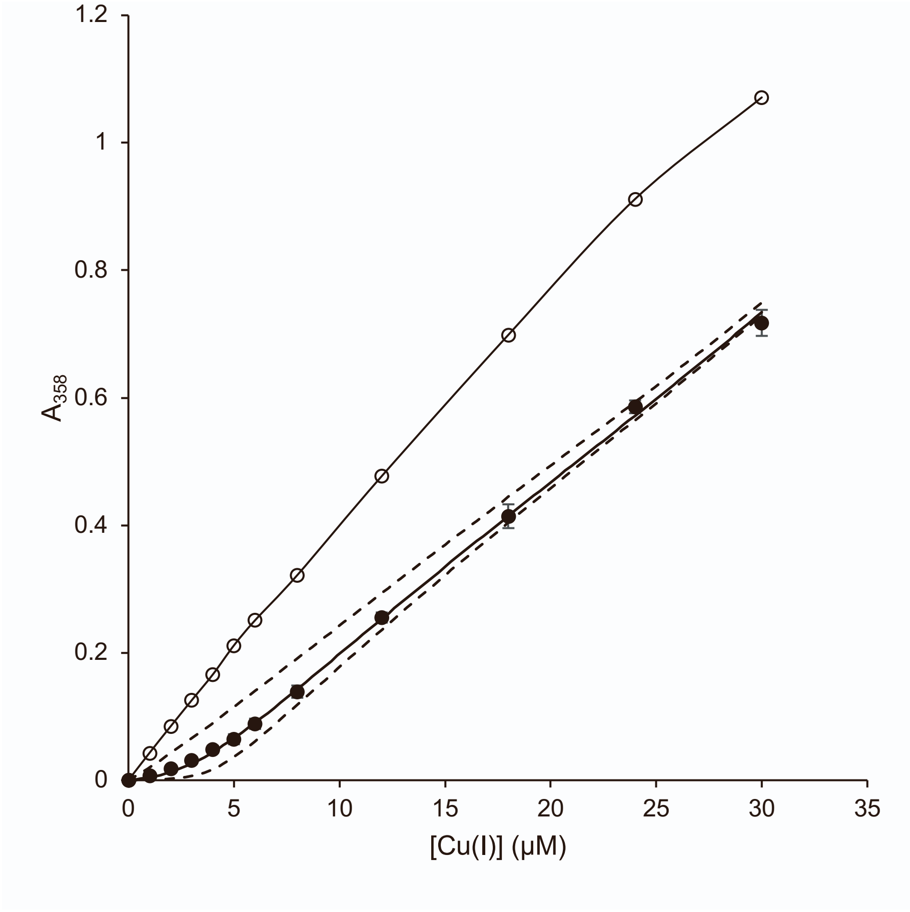

Figure S11. Complete data set for Figure 5F.

$A_{358\ nm}$ binding isotherm of BCA (50 μM) in the presence and absence of 10 μM MBP-PLS (filled and empty circles respectively) upon titration with $CuSO_4$ (recorded anaerobically at pH 7.0) in the presence of 1 mM NH_2OH. Dynafit model (solid line) describes Cu(I)-binding of MBP-PLS when modelled as forming a 2:1 complex with a β_2 value of 3.79 (±1.5) x10^{19} M^{-2} and one additional weak binding event. Dotted lines above and below represent the same model when the affinity is simulated 10x weaker or tighter, respectively (n = 3, ± SD).

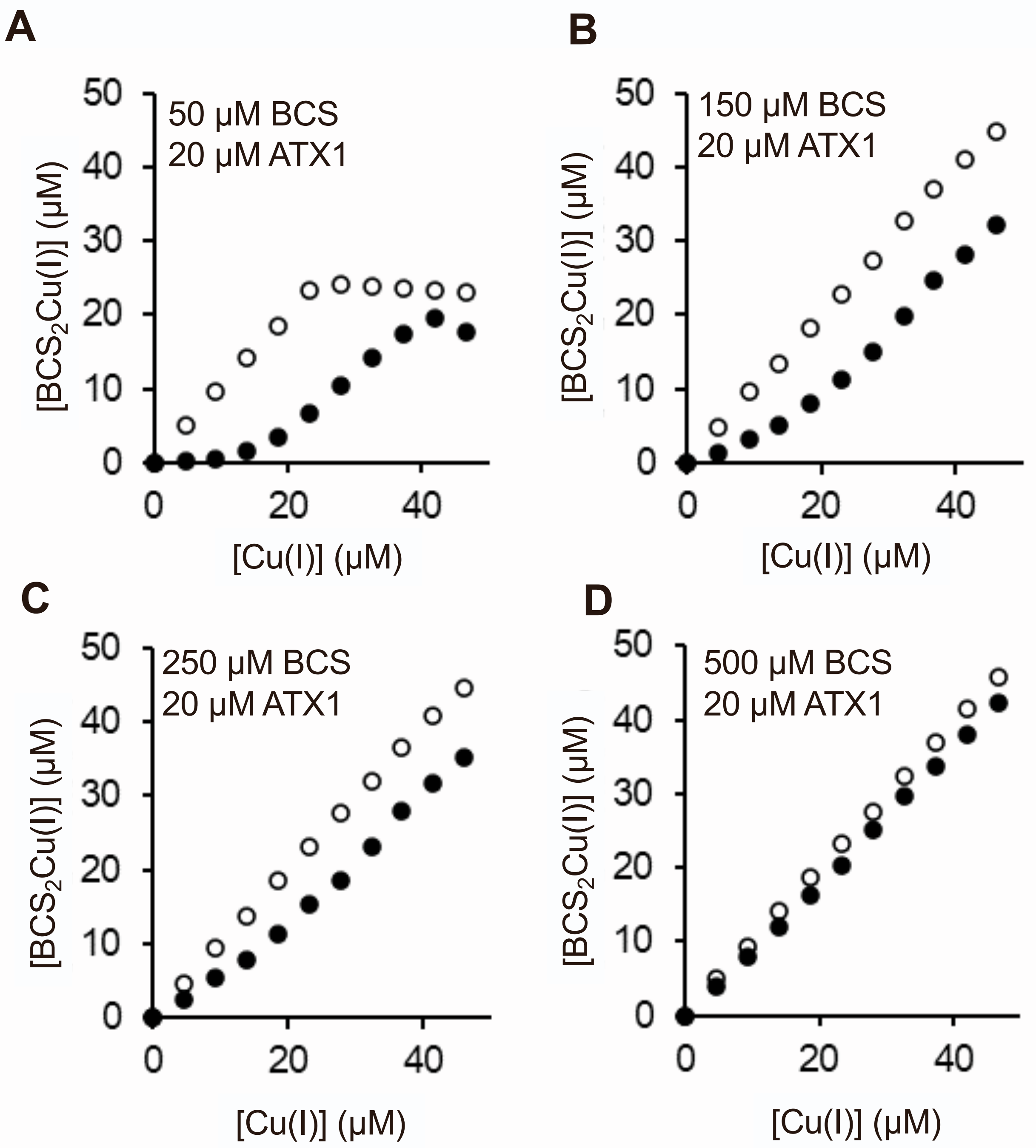

Figure S12. Determination of the Cu(I) binding affinity of the *Arabidopsis* ATX1.

Binding isotherms at A_{483nm} when BCS (50 μM **(A)**, 15 μM **(B)**, 250 μM **(C)** or 500 μM **(D)**) in the presence and absence of 20 μM ATX1 (filled and empty circles, respectively), is titrated with Cu(I) under anaerobic conditions. Using the observed Cu(I):ATX1 stoichiometry of 1:1 (Fig. 5H), affinities of 5.79 $x10^{-18}$, 4.83 $x10^{-18}$, 5.10 $x10^{-18}$, 6.14 $x10^{-18}$ respectively, with a mean of 5.47 (±0.6) $x10^{-18}$ M, were calculated.

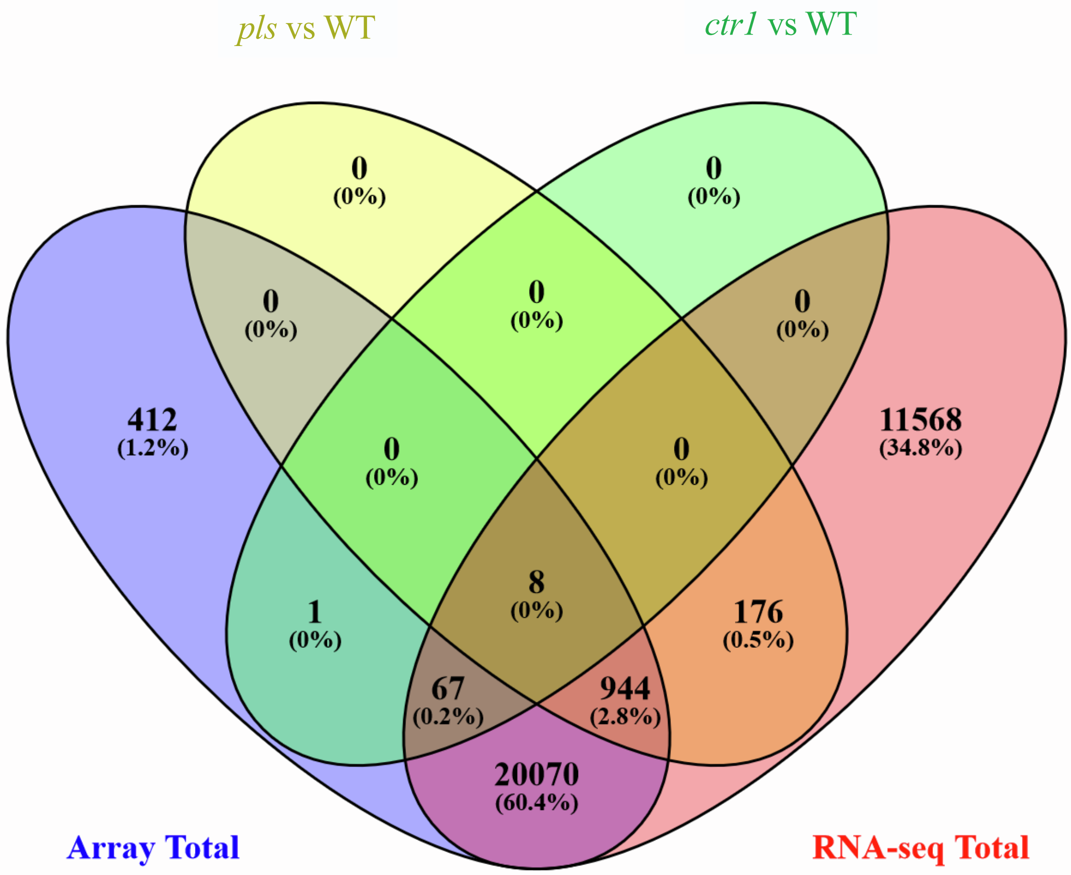

Figure S13. Overlap of DEGs (compared to respective wild types) between the *ctr1* and *pls* mutants.

Venn diagram generated from DEG lists generated by Brodersen *et al*. (2006) *Plant J*. **47**, 532-546, Table S2 for *ctr1 vs.* wild type, and data from Table S1 (this paper) for *pls vs.* wild type. Overlap between the CTR1- and PLS-regulated genes (i.e upregulated in both *ctr1* and *pls* mutants compared to wild type) is significant. P value of Fisher's exact test (alternative='greater') = 0.01959. 21089 genes are analysed in both experiments, of which the proportion of CTR1-regulated genes is 8+67/21089=0.36%. The proportion of CTR1-regulated genes in PLS-regulated genes is increased to 8/(8+944)=0.84%.

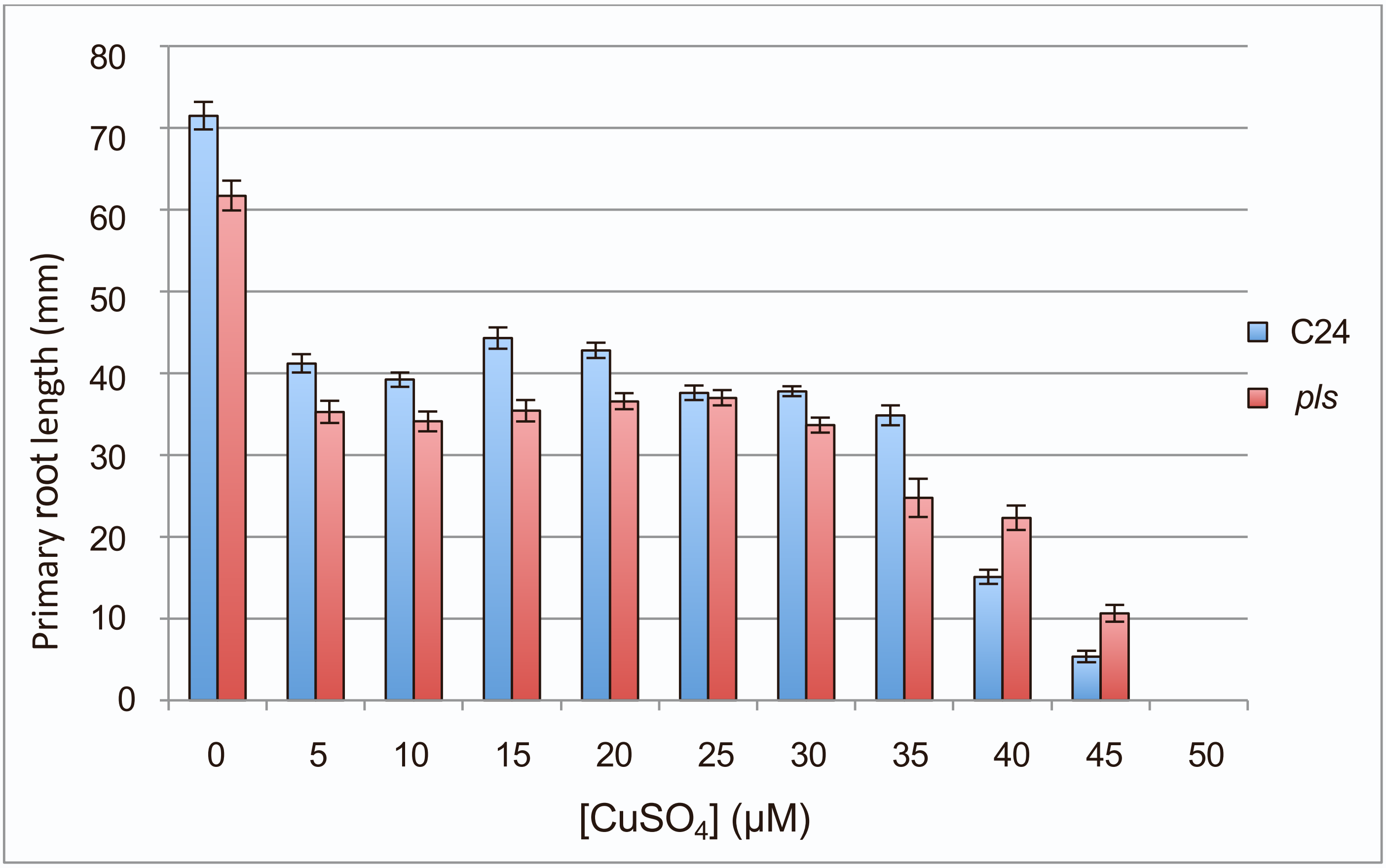

Figure S14. The *pls* mutant primary root is not hypersensitive to exogenous copper.

Seedlings were grown for 12 days in liquid ½ MS10 medium supplemented with different concentrations of $CuSO_4$. Root length (n = 18 from samples grown on 3 independent plates) was measured after 12 days using ImageJ. Error bars show means ± 1 standard error. Red bars represent *pls* mutant, blue bars represent wild type.

A

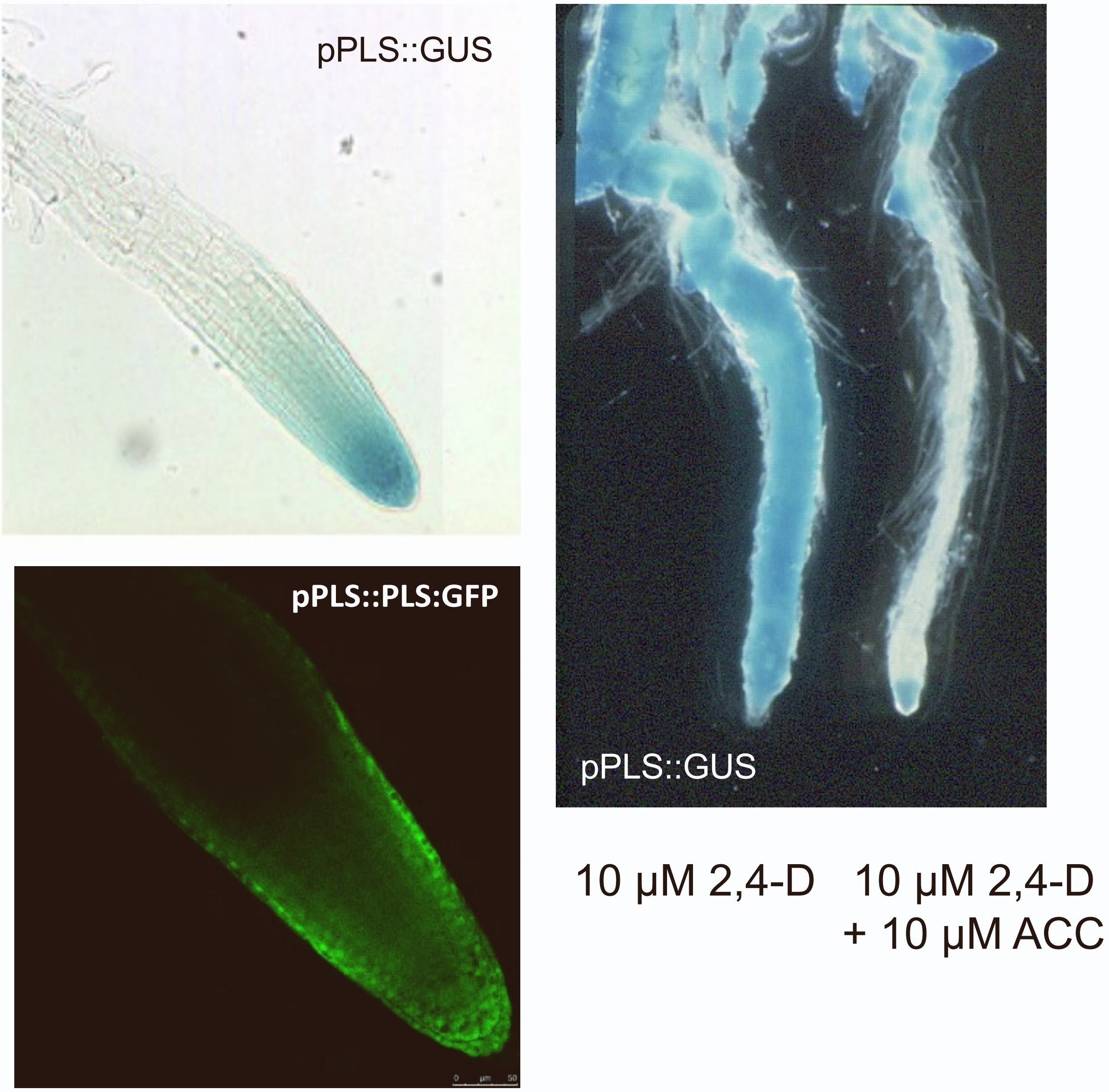

Figure S15. The link between auxin and ethylene signalling effects on *PLS* transcription, and downstream effects on ethylene responses.

(A) Antagonism between auxin and ethylene on PLS expression.
Auxin positively regulates *PLS* gene transcription, while ACC (ethylene precursor) treatment antagonizes the inductive effects of auxin. Left upper panel: pPLS::GUS expression in the Arabidopsis root tip under standard growth conditions. Left lower panel: pPLS::PLS:GFP expression in the Arabidopsis root tip under standard growth conditions. Right panel: pPLS::GUS expression in root tips treated with 10 µM 2,4-D, showing ectopic expression throughout the root (left); and pPLS::GUS expression in root tips treated with 10 µM 2,4-D plus 10 µm ACC, showing the antagonism of the inductive effect of 2,4-D on PLS::GUS expression. Images are representative of 10 independently grown roots.

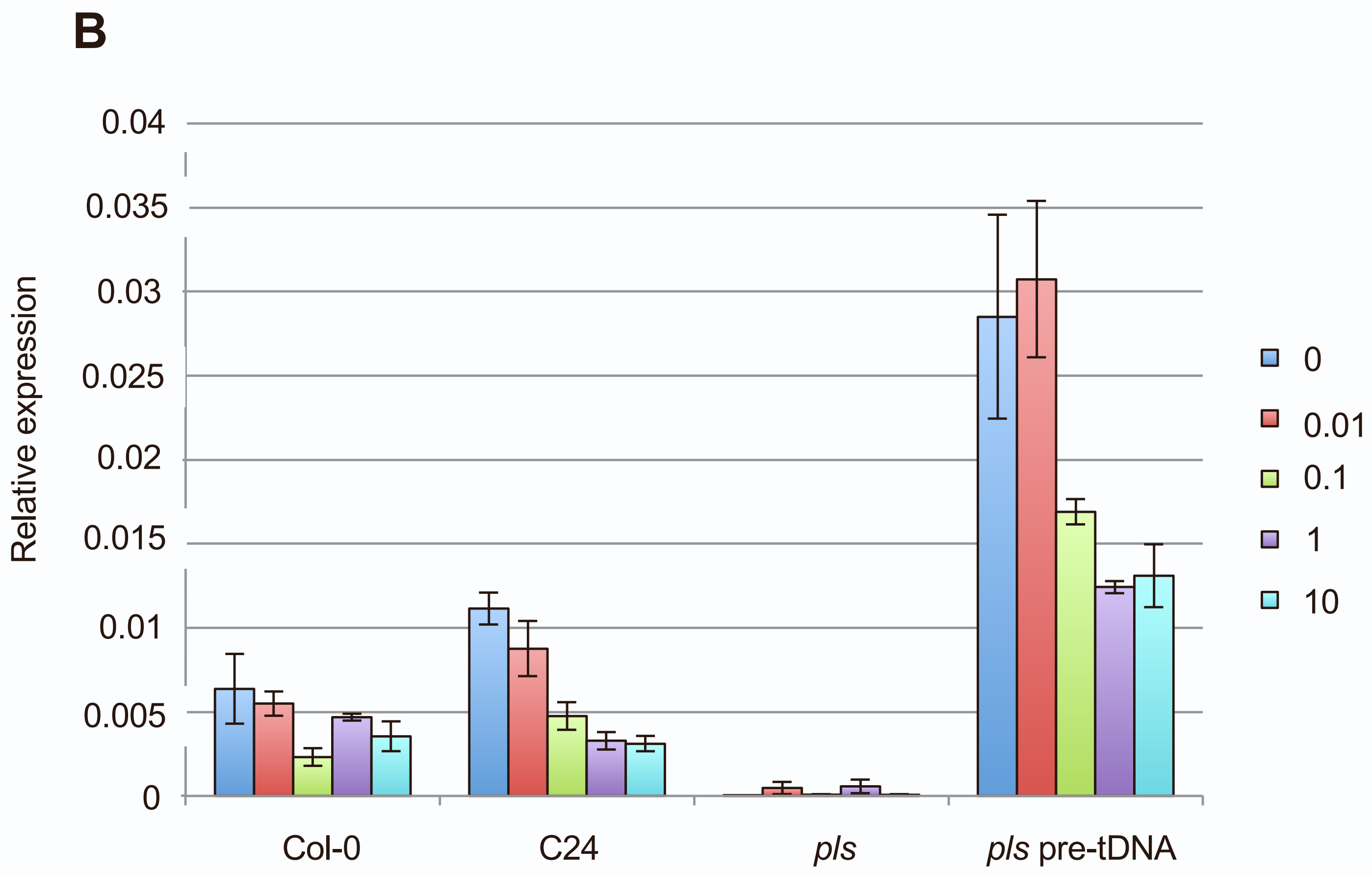

Figure S15. The link between auxin and ethylene signalling effects on *PLS* transcription, and downstream effects on ethylene responses.

(B) *PLS* gene expression under ACC treatment.
Col-0, C24 and *pls* seedlings were treated with 0, 0.01, 0.1, 1 or 10 µM ACC for seven d.a.g and quantitative PCR was carried out on the resulting cDNA. *PLS* transcript levels were measured alongside a reference gene transcript *PP2C.* Comparison is made between the *PLS* expression in Col-0 and C24 wild types and the *pls* mutant, in which the reverse qPCR primer is located beyond the tDNA insertion in the *pls* mutant. In the fourth set of bars, a second set of *PLS* primers were used, located before the tDNA insertion, to detect a truncated and inactive *PLS* transcript. Error bars show means ±1 standard error, n = 3 biological replicates with three technical replicates.

C

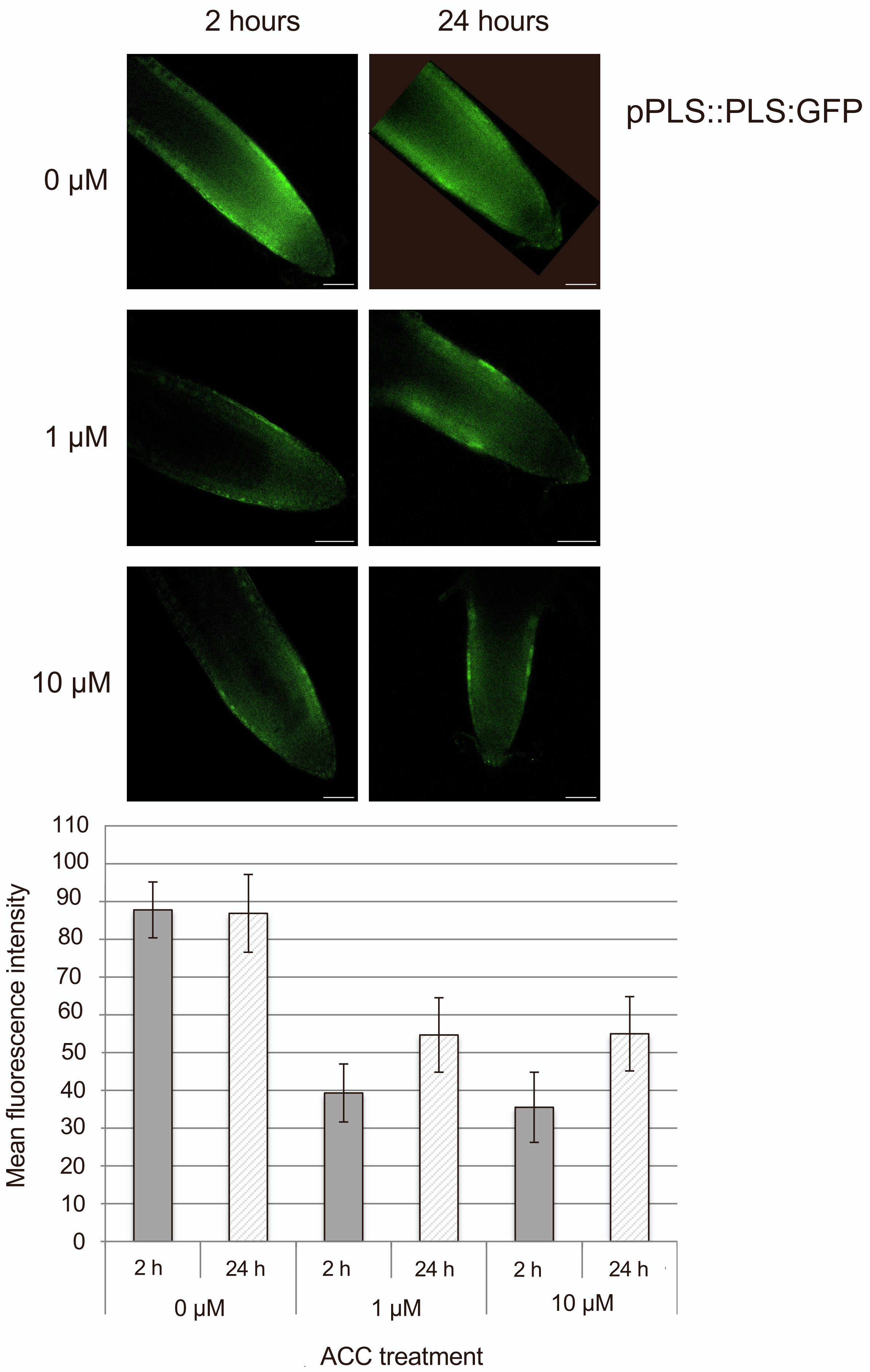

2 hours
24 hours
pPLS::PLS:GFP
0 µM
1 µM
10 µM
Mean fluorescence intensity
110
100
90
80
70
60
50
40
30
20
10
0
2 h
24 h
2 h
24 h
2 h
24 h
0 µM
1 µM
10 µM
ACC treatment

Figure S15. The link between auxin and ethylene signalling effects on *PLS* transcription, and downstream effects on ethylene responses.

(**C**) **Expression of the PLS:GFP fusion protein is downregulated in the root tip after ACC treatment.**

Upper panel: Transgenic seedlings were treated with 0, 1 or 10 μM ACC for either 2 or 24 hours and imaged by CLSM at seven d.a.g. Laser settings were maintained at 21% 488 nm 20 mW, 970 V gain. Images are representative of 10 independetly grown roots. Scale bars = 50 μm.

Lower panel: Quantification of PLS-GFP fluorescence after ACC treatment, detected in seedlings expressing the *pPLS::PLS:GFP* construct. Seedling root tips at seven d.a.g were imaged by CLSM after treatment with 0, 1 or 10 μM ACC for either 2 or 24 hours. Fluorescence intensity in each root was measured using ImageJ and the mean intensity for each treatment was calculated. Error bars show ± 1 standard error, n = 17.

Supplementary Tables

Table S1. Differentially expressed genes between *pls* mutant and wild type (see video file).

Table S2. Differentially expressed genes between *PLS* transgenic overexpresser (PLSOx) and wild type (see video file).

Table S3. GO terms upregulated in *pls* mutant vs. wild type (see video file).

Table S4. GO terms downregulated in *pls* mutant vs. wild type (see video file).

Table S5. GO terms upregulated in *PLS* transgenic overexpresser (PLSOx) vs. wild type (see video file).

Table S6. GO terms downregulated in *PLS* transgenic overexpresser (PLSOx) vs. wild type (see video file).

Table S7. 120 GO terms unregulated in pls compared to wild type, and compared to PLSOx.

GO term	*pls* mutant upregulated cf WT GO term	FDR		GO term PLS OX downregulated cf WT	FDR
GO:0050896	response to stimulus	1.60E-37		response to stimulus	0.00013
GO:0006950	response to stress	3.90E-33		response to stress	0.34
GO:0006952	defense response	1.20E-28		defense response	1.0
GO:0044699	single-organism process	1.00E-25		single-organism process	5.5E-14
GO:1901700	response to oxygen-containing compound	1.30E-20		response to oxygen-containing compound	0.19
GO:0010243	response to organonitrogen compound	5.40E-20		response to organonitrogen compound	Not detected
GO:0042221	response to chemical	2.30E-19		response to chemical	0.012
GO:0010200	response to chitin	2.30E-19		response to chitin	Not detected
GO:0009987	cellular process	5.50E-17		cellular process	2.20E-20
GO:1901698	response to nitrogen compound	1.10E-16		response to nitrogen compound	1
GO:0044763	single-organism cellular process	4.00E-16		single-organism cellular process	1.80E-11
GO:0009605	response to external stimulus	5.20E-16		response to external stimulus	1
GO:0051707	response to other organism	2.10E-15		response to other organism	1
GO:0043207	response to external biotic stimulus	2.10E-15		response to external biotic stimulus	1
GO:0009617	response to bacterium	2.50E-15		response to bacterium	1
GO:0009607	response to biotic stimulus	5.10E-15		response to biotic stimulus	1
GO:0006468	protein phosphorylation	1.80E-13		protein phosphorylation	1
GO:0008152	metabolic process	4.00E-13		metabolic process	7.90E-11
GO:0002376	immune system process	1.00E-12		immune system process	1
GO:0044710	single-organism metabolic process	1.20E-12		single-organism metabolic process	1.60E-06
GO:0051704	multi-organism process	1.50E-12		multi-organism process	1
GO:0016310	phosphorylation	1.50E-12		phosphorylation	1
GO:0051716	cellular response to stimulus	1.90E-12		cellular response to stimulus	1
GO:0045087	innate immune response	3.50E-12		innate immune response	1
GO:0010033	response to organic substance	5.30E-12		response to organic substance	0.064
GO:0006955	immune response	8.30E-12		immune response	1
GO:0009628	response to abiotic stimulus	1.00E-11		response to abiotic stimulus	5.00E-14
GO:0009657	plastid organization	6.60E-11		plastid organization	6.00E-07
GO:0098542	defense response to other organism	1.50E-10		defense response to other organism	1
GO:0042742	defense response to bacterium	1.80E-10		defense response to bacterium	1
GO:0001101	response to acid chemical	2.10E-10		response to acid chemical	0.098

GO term	*pls* mutant upregulated cf WT GO term	FDR		GO term PLS OX downregulated cf WT	FDR
GO:0006793	phosphorus metabolic process	3.70E-10		phosphorus metabolic process	1
GO:0009719	response to endogenous stimulus	4.20E-10		response to endogenous stimulus	0.41
GO:0007154	cell communication	4.20E-10		cell communication	1
GO:0006796	phosphate-containing compound metabolic process	4.40E-10		phosphate-containing compound metabolic process	1
GO:0044711	single-organism biosynthetic process	2.00E-09		single-organism biosynthetic process	4.50E-06
GO:0009658	chloroplast organization	4.40E-09		chloroplast organization	4.20E-06
GO:0071704	organic substance metabolic process	1.00E-08		organic substance metabolic process	1.80E-06
GO:0007165	signal transduction	1.10E-08		signal transduction	1
GO:0043412	macromolecule modification	1.20E-08		macromolecule modification	1
GO:0044237	cellular metabolic process	2.00E-08		cellular metabolic process	3.40E-13
GO:0044700	single organism signaling	2.30E-08		single organism signaling	1
GO:0023052	signaling	2.40E-08		signaling	1
GO:0006979	response to oxidative stress	1.30E-07		response to oxidative stress	1
GO:0009611	response to wounding	2.10E-07		response to wounding	1
GO:0006464	cellular protein modification process	6.20E-07		cellular protein modification process	1
GO:0036211	protein modification process	6.20E-07		protein modification process	1
GO:0019748	secondary metabolic process	1.00E-06		secondary metabolic process	1
GO:0008219	cell death	1.70E-06		cell death	Not detected
GO:0009814	defense response, incompatible interaction	3.10E-06		defense response, incompatible interaction	Not detected
GO:0044550	secondary metabolite biosynthetic process	5.00E-06		secondary metabolite biosynthetic process	1
GO:0071456	cellular response to hypoxia	5.00E-06		cellular response to hypoxia	Not detected
GO:0043436	oxoacid metabolic process	6.70E-06		oxoacid metabolic process	0.036
GO:0000302	response to reactive oxygen species	8.00E-06		response to reactive oxygen species	0.73
GO:0052542	defense response by callose deposition	8.00E-06		defense response by callose deposition	Not detected
GO:0036294	cellular response to decreased oxygen levels	8.00E-06		cellular response to decreased oxygen levels	Not detected
GO:0071453	cellular response to oxygen levels	8.00E-06		cellular response to oxygen levels	Not detected
GO:0052482	defense response by cell wall thickening	1.60E-05		defense response by cell wall thickening	Not detected

GO term	*pls* mutant upregulated cf WT GO term	FDR		GO term PLS OX downregulated cf WT	FDR
GO:0052544	defense response by callose deposition in cell wall	1.60E-05		defense response by callose deposition in cell wall	Not detected
GO:0070887	cellular response to chemical stimulus	1.80E-05		cellular response to chemical stimulus	1
GO:0055114	oxidation-reduction process	2.60E-05		oxidation-reduction process	6.20E-05
GO:0010035	response to inorganic substance	2.70E-05		response to inorganic substance	0.00081
GO:0031347	regulation of defense response	2.90E-05		regulation of defense response	1
GO:0006082	organic acid metabolic process	3.10E-05		organic acid metabolic process	0.025
GO:0031425	chloroplast RNA processing	4.00E-05		chloroplast RNA processing	Not detected
GO:0044281	small molecule metabolic process	5.20E-05		small molecule metabolic process	0.0039
GO:0080134	regulation of response to stress	5.20E-05		regulation of response to stress	1
GO:0019752	carboxylic acid metabolic process	7.00E-05		carboxylic acid metabolic process	0.036
GO:0001666	response to hypoxia	0.0001		response to hypoxia	Not detected
GO:0042430	indole-containing compound metabolic process	0.0001		indole-containing compound metabolic process	Not detected
GO:0033554	cellular response to stress	0.00012		cellular response to stress	1
GO:0010193	response to ozone	0.00022		response to ozone	Not detected
GO:0036293	response to decreased oxygen levels	0.00022		response to decreased oxygen levels	Not detected
GO:0070482	response to oxygen levels	0.00024		response to oxygen levels	Not detected
GO:0009266	response to temperature stimulus	0.00025		response to temperature stimulus	1.30E-07
GO:0052545	callose localization	0.00025		callose localization	Not detected
GO:0012501	programmed cell death	0.00029		programmed cell death	Not detected
GO:0052543	callose deposition in cell wall	0.00029		callose deposition in cell wall	Not detected
GO:0009793	embryo development ending in seed dormancy	0.0003		embryo development ending in seed dormancy	1
GO:0009725	response to hormone	0.0003		response to hormone	0.22
GO:0052386	cell wall thickening	0.00035		cell wall thickening	Not detected
GO:0033037	polysaccharide localization	0.0004		polysaccharide localization	Not detected
GO:0009751	response to salicylic acid	0.00055		response to salicylic acid	1
GO:0009723	response to ethylene	0.00062		response to ethylene	1
GO:0044238	primary metabolic process	0.00063		primary metabolic process	2.70E-05
GO:0042391	regulation of membrane potential	0.00063		regulation of membrane potential	Not detected
GO:0009790	embryo development	0.00077		embryo development	1

GO term	*pls* mutant upregulated cf WT GO term	FDR		GO term PLS OX downregulated cf WT	FDR
GO:0065007	biological regulation	0.00099		biological regulation	0.5
GO:0010304	PSII associated light-harvesting complex II catabolic process	0.001		PSII associated light-harvesting complex II catabolic process	Not detected
GO:0006520	cellular amino acid metabolic process	0.0011		cellular amino acid metabolic process	1
GO:0010154	fruit development	0.0012		fruit development	1
GO:0032501	multicellular organismal process	0.0013		multicellular organismal process	0.068
GO:0009817	defense response to fungus, incompatible interaction	0.0013		defense response to fungus, incompatible interaction	Not detected
GO:0071369	cellular response to ethylene stimulus	0.0013		cellular response to ethylene stimulus	1
GO:0048583	regulation of response to stimulus	0.0013		regulation of response to stimulus	1
GO:0006801	superoxide metabolic process	0.0013		superoxide metabolic process	Not detected
GO:0048316	seed development	0.0014		seed development	1
GO:0042793	transcription from plastid promoter	0.0014		transcription from plastid promoter	Not detected
GO:0050789	regulation of biological process	0.0015		regulation of biological process	0.046
GO:0048731	system development	0.002		system development	0.18
GO:0050794	regulation of cellular process	0.0025		regulation of cellular process	0.025
GO:0007275	multicellular organism development	0.0031		multicellular organism development	0.019
GO:0009873	ethylene-activated signaling pathway	0.0032		ethylene-activated signaling pathway	1
GO:0016143	S-glycoside metabolic process	0.0032		S-glycoside metabolic process	Not detected
GO:0042343	indole glucosinolate metabolic process	0.0032		indole glucosinolate metabolic process	Not detected
GO:0009620	response to fungus	0.0033		response to fungus	1
GO:0000160	phosphorelay signal transduction system	0.0034		phosphorelay signal transduction system	1
GO:0043170	macromolecule metabolic process	0.0034		macromolecule metabolic process	0.038
GO:0009626	plant-type hypersensitive response	0.0035		plant-type hypersensitive response	Not detected
GO:0009816	defense response to bacterium, incompatible interaction	0.0037		defense response to bacterium, incompatible interaction	Not detected
GO:0016053	organic acid biosynthetic process	0.0037		organic acid biosynthetic process	0.023
GO:0048544	recognition of pollen	0.0037		recognition of pollen	Not detected

GO term	*pls* mutant upregulated cf WT GO term	FDR		GO term PLS OX downregulated cf WT	FDR
GO:0034050	host programmed cell death induced by symbiont	0.0037		host programmed cell death induced by symbiont	Not detected
GO:0044283	small molecule biosynthetic process	0.004		small molecule biosynthetic process	0.002
GO:0009636	response to toxic substance	0.004		response to toxic substance	1
GO:0071451	cellular response to superoxide	0.0044		cellular response to superoxide	Not detected
GO:0071450	cellular response to oxygen radical	0.0044		cellular response to oxygen radical	Not detected
GO:0098869	cellular oxidant detoxification	0.0044		cellular oxidant detoxification	Not detected
GO:1990748	cellular detoxification	0.0044		cellular detoxification	Not detected
GO:0019430	removal of superoxide radicals	0.0044		removal of superoxide radicals	Not detected

Table S8. Downregulated genes of GO term response to metal ion in pls mutant

GO term/gene	Protein function	FDR	Pvalue
GO:0010038 response to metal ion		8.5E-07	5.70E-05
AT5G24770	acid phosphatase activity dependent on the presence of divalent cations (Mg2+, Co2+, Zn2+, Mn2+) and anti-insect activity		
AT2G30860	GSTF9 glutathione S-transferase, metal responsive oxidative stress		
AT5G14545	MIR398b downregulated by biotic and abiotic stress		
AT1G11840	GLX1 glyoxalase/bleomycin resistance protein/ dioxygenase superfamily protein - link to water stress		
AT5G67300	MYBR1 mediates abiotic stress responses such as salt stress, oxidative stress		
AT3G56240	CCH copper chaperone involved in Cu homeostasis		
AT5G59780	MYB59 induced by cadmium (Cd) and plays a key role in the regulation of cell cycle progression and root elongation, controlling plant growth and stress responses		
AT4G25100	Fe-superoxide dismutase - required for oxidative stress tolerance		

GO term/gene	Protein function	FDR	Pvalue
AT3G13782	NAP1,4 - nucleosome assembly protein - required for resistance to genotoxic stresses such as UV		
AT1G35720	annexin and Ca transporter with peroxidase activity, links ROS and Ca signalling		
AT3G51860	CAX3 - a Ca transporter with role in phosphate homeostasis and metal sequestration		
AT1G07610	cysteine rich metallothionin required for heavy metal tolerance		
AT4G23670	Cu-binding polyketide cyclase/ dehydrase and lipid transport protein involved in abiotic stress		
AT3G16450	mannose-binding lectin involved in root cap salt stress response		
AT4G04460	PASPA3, Saposin-like aspartyl protease involved in PCD		
AT4G13430	methylthioalkyl malate isomerase involved in glucosinolate biosynthesis		
AT3G03780	putative methionine synthase		
AT1G07590	Tetratricopeptide repeat (TPR)-like superfamily protein - link to ABA signalling and osmotic stress		
AT4G37260	MYB73, role in salt stress		

Table S9.
Primers for RT-qPCR

Gene Forward/Reverse	Primer sequence 5'-3'	Tm (ºC)
PLS Forward	CAGAGAGAAAGAGAAGAGCACG	58.5
PLS Reverse	TAATTTCAGGCGAAGGTCCAT	57.4
PLS pre-T-DNA Forward	GCAGTGTCTCACTGAAACATG	57.5
PLS pre-T-DNA Reverse	CAATGGATTTTAAAAAGTTTAAA CAATTTTGC	58.3
ERF11 Forward	AGCACCGTGGAATCATCGTT	60.04
ERF11 Reverse	CCATCACCACCGACGAAGAA	60.04
ERF19 Forward	CCACCGGTGAAAGTCAGTCA	59.89
ERF19 Reverse	TTCACGCTGGTACTGTGGAC	59.97
ERF61 Forward	CTCCGTCTATCTCCGCCAAC	59.97
ERF61 Reverse	GAACGATGGCATCCTCGCTA	59.97
TDR1 Forward	CAGAGATTCGAGACCCGTCG	59.97
TDR1 Reverse	AGCGAGATGACCCCTAAGGT	60.03
PP2C Forward	AGCAGGGTGAGGATTTGGTG	59.4
PP2C Reverse	ATTCACCTGGCAAATCCGGT	57.3
ACTIN2 Forward	GGATCGGTGGTTCCATTCTTGC	56
ACTIN2 Reverse	AGAGTTTGTCACACACAAGTGCA	55

Primers for *PLS::PLS* cloning from previously cloned sequence in TOPO2.1 (Casson et al. 2002)

PLS Forward:

GGGGACAAGTTTGTACAAAAAAGCAGGCTTCAAGCTTTAGCCCGTGCGG

PLS Reverse:

GGGGACCACTTTGTACAAGAAAGCTGGGTCATGGATTTTAAAAAGTTTAAACAATTTTGCTACTAATAA

ATAAG

Primers for cloning *ETR1* and *PLS* sequences for yeast 2-hybrid and co-immuniprecipitation

ETR1:

Forward primer GAA TCC ATG GAA GTC TGC AAT TGT A (Eco RI on 5' end)

Reverse primer GTC GAC TTA CAT GCC CTC GTA CA (Sal I on 5'end)

PLS:

Forward primer CTG GAG ATG AAA CCC AGA CTT TGT (Xho I on 5' end)

Reverse primer GTC GAC ATG GAT TTT AAA AAG TTT (Sal I on 5' end)
