# Supplementary material for: POLARIS is a copper-binding peptide that interacts with ETR1 to negatively regulate ethylene signaling in Arabidopsis
Source: Plant Commun. 2025 Jun 25;6(12):101432. doi: 10.1016/j.xplc.2025.101432 (PMC12744750; doi:10.1016/j.xplc.2025.101432)
Supplement: Document S2. Article plus supplemental information [file mmc8.pdf]

Plant Communications
Research article

CellPress Partner Journal

POLARIS is a copper-binding peptide that interacts with ETR1 to negatively regulate ethylene signaling in *Arabidopsis*

Anna J. Mudge[1,9], Saher Mehdi[1,4,9], Will Michaels[1,2,9], Beatriz Orosa-Puente[1,5,6], Weiran Shen[1], Charlie Tomlinson[2,7], Wenbin Wei[1], Claudia Hoppen[3], Buket Uzun[3], Dipan Roy[1], Flora M. Hetherington[1,8], Jennifer F. Topping[1], Ari Sadanandom[1], Georg Groth[3], Nigel J. Robinson[1,2,*] and Keith Lindsey[1,*]

[1]Department of Biosciences, Durham University, Durham DH1 3LE, UK

[2]Department of Chemistry, Durham University, Durham DH1 3LE, UK

[3]Institute of Biochemical Plant Physiology, Heinrich Heine University Düsseldorf, 40204 Düsseldorf, Germany

[4]Present address: wellOwise Research and Development, IIITD Innovation & Incubation Centre, New Delhi, Delhi 110020, India

[5]Present address: School of Biological Sciences, University of Edinburgh, Edinburgh EH9 3FF, UK

[6]Present address: Centro Singular de Investigación en Química Biolóxica e Materiais Moleculares (CIQUS) and Departamento de Química Orgánica, 15782, Universidade de Santiago de Compostela, Spain

[7]Present address: Department of Biology, University of York, Heslington, York YO10 5DD, UK

[8]Present address: University of Bath, Claverton Down, Bath BA2 7AY, UK

[9]These authors contributed equally to this article.

***Correspondence: Nigel J. Robinson (nigel.robinson@durham.ac.uk), Keith Lindsey (keith.lindsey@durham.ac.uk)**

https://doi.org/10.1016/j.xplc.2025.101432

ABSTRACT

Ethylene signaling is one of the classic hormonal pathways in plants, with diverse roles in development and stress responses. The dimeric ethylene receptor localizes to the endoplasmic reticulum and contains Cu(I) ions essential for ethylene binding and signal transduction. We previously discovered that mutants of the *Arabidopsis* gene *POLARIS* (*PLS*), encoding a 36-amino-acid peptide, exhibit enhanced ethylene signaling responses suggestive of reduced receptor activity, but the role and activity of the PLS peptide in this signaling cascade have not been defined. Here, we report that *Arabidopsis* PLS binds copper as a 1:2 thiol-dependent Cu(I):PLS_2 complex with an affinity of 3.79 (±1.5) × 10^{19} M^{-2} via two cysteine residues conserved in the related species *Camelina sativa*. These residues are also essential for biological function. This affinity precludes a role for PLS as a cytosolic Cu chaperone. We demonstrate that PLS localizes to endomembranes and interacts with the transmembrane domain of the receptor protein ETR1. PLS–ETR1 binding is increased in the presence of copper, and this interaction provides a Cu-dependent mechanism for mediating the repression of ethylene responses. Because *PLS* transcription is upregulated by auxin and downregulated by ethylene, PLS–ETR1 interactions also provide a mechanism for modulation of ethylene responses in high-auxin tissues.

Key words: plant hormone signaling, *arabidopsis*, ethylene, protein metalation, hormone receptor

Mudge A.J., Mehdi S., Michaels W., Orosa-Puente B., Shen W., Tomlinson C., Wei W., Hoppen C., Uzun B., Roy D., Hetherington F.M., Topping J.F., Sadanandom A., Groth G., Robinson N.J., and Lindsey K. (2025). POLARIS is a copper-binding peptide that interacts with ETR1 to negatively regulate ethylene signaling in *Arabidopsis*. Plant Comm. **6**, 101432.

INTRODUCTION

Ethylene is a gaseous hormone used by plants to regulate many aspects of development and responses to biotic and abiotic stresses (Johnson and Ecker, 1998). It is perceived by a family of receptors that, in *Arabidopsis*, comprises five members located on the endoplasmic reticulum (ER) (Chen et al., 2002; Grefen et al., 2008): ETR1 (ETHYLENE RESPONSE 1), ERS1 (ETHYLENE RESPONSE SENSOR 1), ERS2, ETR2, and EIN4 (ETHYLENE-INSENSITIVE 4) (Chang et al., 1993; Hua et al.,

 © 2025 The Author(s). Published by Elsevier Inc. on behalf of CAS Center for Excellence in Molecular Plant Sciences, Chinese Academy of Sciences, and Chinese Society for Plant Biology. This is an open access article under the CC BY license (http://creativecommons.org/licenses/by/4.0/).

1995; 1998; Sakai et al., 1998). The receptors are related to bacterial two-component systems (Chen et al., 2002), form dimers through disulfide bonding at the N-terminal hydrophobic domains (Schaller et al., 1995; Hall et al., 2000), and contain Cu (I) ions bound to residues Cys65 and His69, which are essential for ethylene binding and signal transduction (Rodriguez et al., 1999; McDaniel and Binder, 2012). In the absence of ethylene, these receptors activate the negative regulator CTR1 (CONSTITUTIVE TRIPLE RESPONSE 1), which is a mitogen-activated protein kinase kinase kinase (MAPKKK), thus preventing ethylene responses (Chang, 2003; Gao et al., 2003). The mechanisms by which receptor activity is regulated are not fully understood.

Introduction of copper to the ER and ethylene receptor requires the RAN1 (RESPONSIVE TO ANTAGONIST1) protein. This is a predicted copper-transporting P-type ATPase homologous to yeast Ccc2p and human Menkes and Wilson disease proteins (Hirayama et al., 1999). Strong *RAN1* loss-of-function mutants in *Arabidopsis* (e.g., *ran1-3*, *ran1-4*) exhibit an enhanced ethylene signaling response (Binder et al., 2010) consistent with a loss of receptor function and similar to that of higher-order loss-of-function receptor mutants, which also show an ethylene hypersignaling phenotype (Qu et al., 2007). The mechanisms of copper homeostasis at ETR1 are unknown, as is true for other compartmentalized cuproproteins supplied with copper, for example, via Ccc2p, Menkes, or Wilson ATPases. RAN1 in *Arabidopsis* localizes to endomembrane systems, including the *trans*-Golgi and ER compartments, and is necessary for both ethylene-receptor biogenesis and copper homeostasis; loss-of-function *ran1* mutants suggest that copper is required for both ethylene binding and receptor function (Binder et al., 2010). RAN1 can interact directly with ETR1 and the copper chaperones ANTIOXIDANT1 (ATX1) and COPPER CHAPERONE (CCH), suggesting that copper is transported between proteins to deliver it to the ethylene receptor as part of the receptor biogenesis pathway at the ER (Hoppen et al., 2019).

Our understanding of receptor function is still incomplete, however. For example, how is Cu(I) delivery from RAN1 to ETR1 mediated? How does Cu(I) influence receptor conformation and function? Are there other Cu(I)-binding components involved? Is the receptor regulated in a tissue-specific manner or in response to the hormonal environment in a tissue? Is this process part of the crosstalk mechanism with other hormone signaling pathways? It is well established that ethylene signaling interacts with and is affected by other hormonal pathways, but does this influence the receptor metalation state and have developmental consequences?

We previously showed that the loss-of-function *polaris* (*pls*) mutant has some phenotypic similarities to *ran1* loss-of-function alleles and to *ctr1*, exhibiting a triple-response phenotype (short hypocotyl and root, exaggerated apical hook, radial expansion) in the dark in the absence of ethylene (Chilley et al., 2006) and a short-root phenotype in light-grown seedlings, consistent with its known expression in the root meristem of light-grown seedlings. Transgenic complementation of the mutant by the *PLS* gene (AT4G39403), which encodes a 36-amino-acid peptide, suppresses the mutant phenotype (Casson et al., 2002). The *pls* mutant phenotype is rescued by the gain-of-function ethylene-resistant mutation *etr1-1* and by pharmacological inhibition of ethylene signaling by silver ions (Chilley et al., 2006). The *pls* mutant produces ethylene gas at wild-type levels, indicating that the peptide plays a role in ethylene signaling rather than ethylene biosynthesis (Chilley et al., 2006). By contrast, *PLS* transgenic overexpressors (PLSOx seedlings) exhibit suppression of the triple-response phenotype when grown in the presence of the ethylene precursor 1-aminocyclopropane-1-carboxylic acid (ACC), similar to the gain-of-function *etr1-1* mutant, but this suppression is incomplete (PLSOx seedlings show some response to ACC; Casson et al., 2002; Chilley et al., 2006). *PLS* overexpression also partially suppresses the *ctr1* mutant phenotype, indicating that the PLS peptide acts upstream of CTR1 (Chilley et al., 2006).

In this paper, we show that the POLARIS peptide is required for correct ethylene responses, binds copper via two cysteine residues essential for biological function, but is unlikely to act as a metallochaperone intermediary; however, it co-localizes and forms a copper-adduct with ETR1. We suggest that POLARIS provides cell-type control and a crosstalk regulatory mechanism for ethylene responses through its physical interaction with the ethylene receptor.

RESULTS

The POLARIS peptide is a negative regulator of ethylene responses

The PLS peptide is translated from a low-abundance transcript that, in light-grown seedlings, is most strongly expressed in the embryo and silique, seedling root tip, and leaf vascular tissues (supplemental Figure 1), a pattern reflected in promoter–reporter expression patterns (Casson et al., 2002). Seedlings of the *pls* mutant have short roots in the light in air (Figure 1A). They also have shorter hypocotyls and roots than the wild type and have an exaggerated apical hook when grown in the dark in air but are similar to the wild type in the presence of the ethylene precursor ACC (supplemental Figure 2), indicative of an ethylene hyper-responsive phenotype (Chilley et al., 2006). To investigate the *pls* molecular phenotype, we performed an RNA sequencing (RNA-seq) analysis of the loss-of-function *pls* mutant and transgenic PLSOx seedlings to identify differentially regulated genes, comparing each with wild-type seedlings of the same ecotype as controls (Figure 1B and 1C). The *pls* mutant does not express the full-length *PLS* coding sequence owing to a T-DNA insertion in the coding region and therefore shows disrupted PLS function (Casson et al., 2002). By contrast, the PLSOx seedlings express significantly higher levels of *PLS* transcript compared with the wild type ($\log_2$ fold 10.8, p-adj = 2.02×10^{-33}; supplemental Tables 1 and 2). Eight hundred and thirty-six genes were significantly upregulated and 292 downregulated in the *pls* mutant compared with wild-type control seedlings (p-adj < 0.05) (supplemental Table 1). A total of 1487 genes were significantly upregulated and 1281 downregulated in PLSOx seedlings compared with wild-type controls (p-adj < 0.05; supplemental Table 2). Gene Ontology (GO) analysis of genes upregulated in *pls* mutant seedlings compared with wild-type seedlings showed significant enrichment of genes associated with responses to hormone

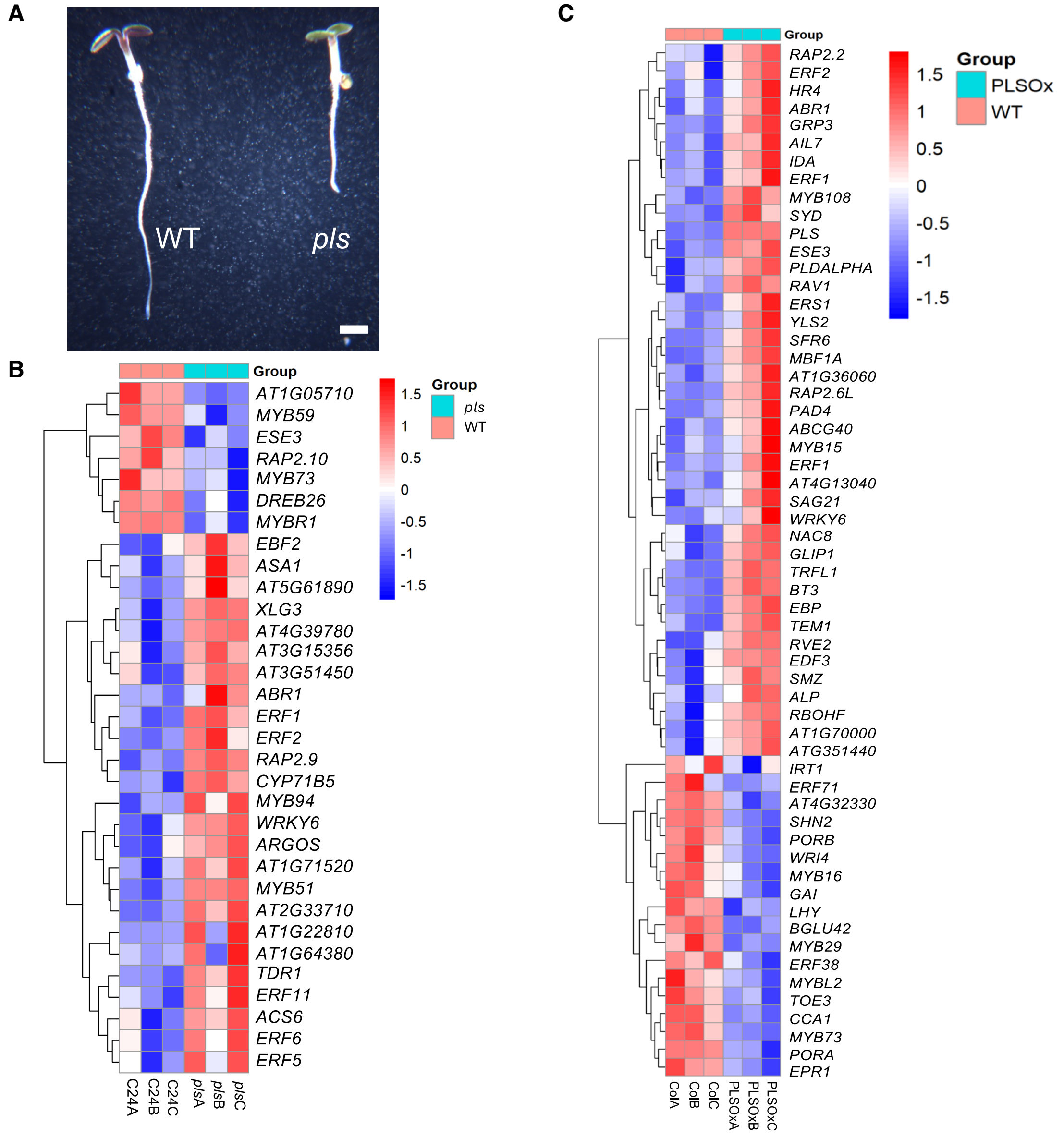

Figure 1. The PLS peptide is required for ethylene control of seedling growth.

(A) Wild type (ecotype C24, left) and *pls* mutant (in C24 background, right). Scale bar corresponds to 5 mm.

(B and C) Heat maps showing expression levels of 32 ethylene-responsive genes in *pls* **(B)** and 58 genes in *PLS* overexpressing (PLSox in Col-0 background) seedlings **(C)** compared with wild-type (Col-0) levels. Data for three biological replicates (**A**, **B**, and **C**; independent seedling samples from which RNA was extracted and its expression analyzed and used for statistical analysis) are shown and are expressed as $\log_2$-fold changes in *pls* mutant and PLSox seedlings compared with the wild type, with corresponding significance levels (*P*-adj values) provided in supplemental Tables 1 and 2. $P < 0.05$ and $\log_2$(fold change) of ±0.5 were used to identify differentially expressed genes.

signaling, biotic and abiotic defense responses, and cell death (supplemental Table 3).

Out of 307 genes annotated with the GO term GO:0009723 (response to ethylene), 25 were significantly upregulated and 7 downregulated in the *pls* mutant compared with the wild type, and 40 were upregulated and 18 downregulated in the PLSOx seedlings (Figure 1C; supplemental Tables 3, 4, 5, and 6; Mudge et al., 2024), indicating that control over *PLS* expression levels is required for correct ethylene responses. While GO:0009723 (response to ethylene) was significantly enriched in genes upregulated in the *pls* mutant compared with the wild type (FDR = 0.00062; supplemental Table 3), a large number of upregulated genes in *pls* were significantly associated with immunity, response to pathogens, and the hypersensitive response (supplemental Table 3). Downregulated genes in *pls* were significantly enriched in GO terms such as hormone biosynthetic process (GO:0042446, FDR = 0.038), hormone metabolic process (GO:0042445, FDR = 0.0096), and regulation of hormone levels (GO:0010817, FDR = 0.0013) (supplemental Table 4; Mudge et al., 2024), consistent with our previous studies that described PLS-dependent crosstalk among ethylene, auxin, and

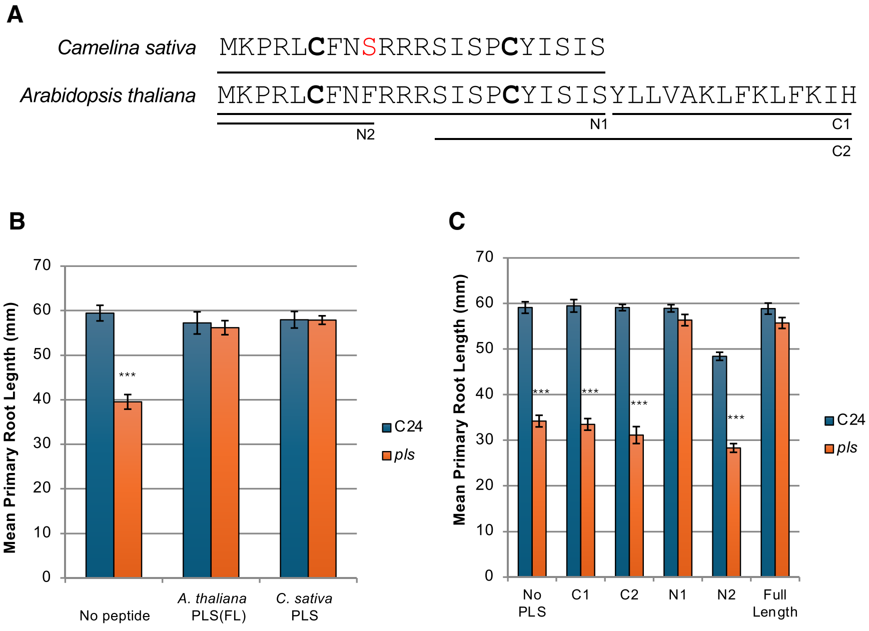

Figure 2. The PLS peptide exhibits structural and functional conservation in Brassicaceae.

(A) Amino acid sequence of the PLS peptide from *Arabidopsis thaliana* with the *Camelina sativa* PLS sequence and the synthetic truncations N1, N2, C1, and C2 indicated by horizontal lines. Two cysteine residues are highlighted in bold.

(B) Effect of the synthetic *Arabidopsis* PLS full-length peptide, *A. thaliana* PLS(FL), and the *Camelina* PLS peptide (*C. sativa* PLS) on *Arabidopsis* primary root length. Wild-type (C24; blue bars) and *pls* mutant seedlings (orange bars) were grown hydroponically in the presence (100 nM) or absence of peptide for 10 days. ***$P < 0.05$, *t*-test between C24 and *pls*. Error bars show ±1 standard error of the mean, $n = 20$.

(C) Effect of synthetic full-length and truncated peptides on primary root length of wild-type (blue bars) and *pls* mutant (orange bars) *Arabidopsis*. Seedlings were grown hydroponically in the presence of 50 nM peptide for 10 days. C1, C-terminal 14 amino acids; C2, C-terminal 24 amino acids; N1, N-terminal 22 amino acids; N2, N-terminal 9 amino acids; full-length PLS, 36 amino acids. Error bars show ±1 standard error of the mean, $n = 25$. ***$P < 0.05$, *t*-test between C24 and *pls*.

cytokinin signaling (Liu et al., 2010; 2013; Moore et al., 2015). In PLSOx overexpressors, both up- and downregulated genes included those associated with GO terms such as “hormone response,” “stress response,” and “immune response.” Genes associated with “photosynthesis” were downregulated in PLSOx seedlings, consistent with repressed photosynthetic activity in the root tips (supplemental Tables 5 and 6; Casson et al., 2002). Interestingly, when comparing the 120 most significantly differentially expressed GO terms upregulated in the *pls* mutant vs. wild type with the same GO terms in PLSOx vs. wild type, we found that most GO terms were restored to wild-type levels (i.e., nonsignificant *P* values between PLSOx and the wild type), although some terms were—notably, “response to stimulus” and “response to abiotic stimulus,” among others (summarized in supplemental Table 7). Four candidate PLS-regulated ethylene-responsive genes identified in the RNA-seq data—*ERF11* (AT1G28370), *ERF19* (AT1G22810), *ERF61* (AT1G64380), and *TDR1* (AT3G23230)—were validated by RT–qPCR and showed consistent upregulation in the *pls* mutant with either nonsignificant or slight downregulation in PLSOx seedlings (supplemental Figure 3). *ERF19*, *ERF61*, and *TDR1* encode integrase-type transcription factors, members of the AINTEGUMENTA-like (AIL) subfamily of the AP2/EREBP family essential for meristem function and stress tolerance (Ma et al., 2024). These data are consistent with a role for PLS in a number of ethylene responses and other related signaling, stress, and developmental processes.

Peptides with high sequence similarity to PLS are found in the Brassicaceae. BLAST searches using the 36-amino-acid PLS peptide sequence as a query identified peptides with significant alignments in *Arabidopsis lyrata* (35 amino acids), *Camelina sativa* (22 amino acids), *Eutrema salsugineum* (28 amino acids), and *Raphanus sativa* (34 amino acids) (supplemental Figure 4) and 36-amino-acid peptides with <50% alignment in *Cardaminopsis suecica* and the hybrid *A. thaliana* × *A. arenosa*. Structural predictions using AlphaFold2 indicated limited structural conservation, but it has been demonstrated that AlphaFold2 is poor at predicting peptide structures (McDonald et al., 2023), and these models should therefore be interpreted with caution.

To better understand the relationship between PLS peptide structure and function between species, we performed hydroponic feeding experiments using synthetic versions of the PLS peptide from *Arabidopsis* and its close relative *C. sativa*. The *C. sativa* gene shows partial sequence identity to the *Arabidopsis PLS* gene, and its predicted 22-amino-acid peptide sequence is identical to the N-terminal 22 amino acids of the *Arabidopsis* PLS, except for a phenylalanine-to-serine substitution at position nine (Figure 2A). We synthesized the full-length PLS peptide, PLS(FL), as well as truncated versions from both *Arabidopsis* and *C. sativa* (Figure 2A), and supplied the peptides hydroponically to *Arabidopsis pls* mutant seedlings. The full-length peptides from both *Arabidopsis* and *C. sativa* and the N-terminal 22-amino-acid sequence of the *Arabidopsis* peptide (N1) were each able to rescue the short primary root length of the *Arabidopsis pls* mutant (Figure 2B); these results were similar to those obtained by transgenic overexpression and genetic complementation using the wild-type *PLS* coding sequence (Casson et al., 2002; Chilley et al., 2006). It is interesting to note that although the predicted structure of the *C. sativa* peptide is quite dissimilar from that of the *Arabidopsis* peptide (supplemental Figure 4), it still retains function in *Arabidopsis.* The mean primary root length of wild-type (C24) seedlings did not differ across all peptide treatments, whereas treatment of *pls* seedlings with 50 nM PLS(FL) and N1 peptides rescued root growth (Figure 2C), and *t*-tests showed that the rescue effects of PLS(FL) and PLS(N1) did not differ significantly ($P > 0.1$, $n = 25$). However, neither a 9-amino-acid sequence (N2, Figure 2C) from the N terminus nor C-terminal sequences of 14 (C1) or 24 (C2) amino acids from *Arabidopsis* PLS were able to rescue the mutant (Figure 2C); each of these shorter peptides

lacked one of the two Cys residues found in the functional longer peptides PLS(FL) and PLS(N1). A fluorescently tagged (5-carboxyfluorescein [5-FAM]) version of the *Arabidopsis* N-terminal 22-amino-acid sequence (N1) was taken up by the roots and also rescued the mutant root phenotype (supplemental Figure 5A and 5B).

PLS localizes to endomembranes, including the endoplasmic reticulum

Because genetic studies suggest that PLS acts close to the ethylene receptor (Chilley et al., 2006), we hypothesized that it would localize to the same subcellular compartment as ETR1. The ethylene receptor in *Arabidopsis* is localized predominantly at the ER (Chang, 2003), and a *proPLS::PLS:GFP*-generated fusion protein (PLS:GFP) was used to investigate sub-cellular localization. Of five independent transformants with a single-copy insertion of the *proPLS::PLS:GFP* gene in the *pls* mutant background, four fully complemented the *pls* mutant (supplemental Figure 6), similar to the wild-type cDNA (Casson et al., 2002), demonstrating the functionality of the gene fusion. PLS:GFP in transgenic plants co-localized with the ER marker dye ER-Tracker Red, which binds sulfonylurea receptors of ATP-sensitive channels on the ER (ThermoFisher; Figure 3A–3C). It also co-localized with the ER lumen–targeted red fluorescent protein RFP:HDEL (Lee et al., 2013) (Figure 3G–3I). These observations suggest that PLS:GFP is located both on the ER membrane and in the lumen. PLS:GFP also appeared to localize to the nucleus and cytoplasm (Figure 3C). As a control, free GFP expressed under the control of the *PLS* promoter did not co-localize to the ER (Figure 3D–3F), and, as expected, the Golgi marker SH:GFP did not co-localize with ER Tracker (Figure 3M–3O). Visualization of *trans*-Golgi-localized SULFOTRANSFERASE1 (ST1) mCherry (Bauer and Papenbrock, 2002) showed that PLS:GFP did not localize to the Golgi (Figure 2J–2L). To identify the side of the ER membrane to which PLS localizes, we performed transient expression of redox-sensitive GFP (roGFP2) fusions of PLS (driven by the CaMV35S promoter, Hoppen et al., 2019). The different excitation properties of roGFP2 in an oxidizing (ER lumen) or reducing environment (cytosol) enabled us to determine the precise location of PLS fused to roGFP2. Ratiometric analysis and comparison with proteins of known localization (i.e., cytosolic roGFP2, ER luminal roGFP2, cytosolic v-SNARE SEC22:GFP, and ER luminal roGFP2:SEC22; Brach et al., 2009) revealed that PLS, as either an N- or C-terminal roGFP2 fusion, resided at the cytosolic side of the ER (as well as other vesicular compartments; Figure 3P). However, there may have been some translocation of PLS:GFP into the ER lumen, as indicated by co-localization with RFP:HDEL. Although the PLS:GFP fusion protein is relatively large compared with the PLS peptide, and this may affect its localization, the fusion was able to complement the *pls* mutation, showing that it is biologically functional. Although it is also formally possible that the GFP moiety is cleaved from the fusion and that the native PLS peptide acts to complement the mutant, the PLS:GFP localization pattern was more specific than that of free GFP or SH-GFP, suggesting that the majority of the fusion peptide localized to the indicated membrane compartments (Figure 3M–3O).

PLS interacts with the ethylene receptor protein ETR1

We hypothesized that PLS plays a role in receptor function and investigated whether this involved direct interaction with the receptor complex. Preliminary yeast 2-hybrid analyses suggested that PLS interacts with ETR1 (supplemental Figure 7). Because there may be some PLS:GFP localization to the nucleus (Figure 3), it is possible that some ETR1 and native PLS also localize to the nucleus to account for the interaction in a yeast 2-hybrid assay. Confirmation of the physical interaction between PLS and ETR1 in plants came from co-immunoprecipitation (CoIP) analysis. *Agrobacterium* containing plasmids encoding PLS linked to a C-terminal GFP, GFP without PLS (control), and ETR1 with a C-terminal HA tag were infiltrated into *Nicotiana benthamiana* leaves for transient expression, each gene under the control of the CaMV35S promoter. After 3 days, the interaction was confirmed by western blotting after CoIP, showing that ETR1:HA was expressed in all samples and that ETR1:HA bound to PLS:GFP. The GFP-only controls did not show binding to ETR1 (Figure 4A). This demonstrates that the interaction is dependent on the presence of the PLS peptide. The addition of 0.5 μM copper sulfate to the protein extract used for CoIP experiments stabilized the PLS–ETR1 interaction. The presence of copper ions resulted in almost three-fold more PLS:GFP detected upon pulldowns with ETR1:HA, or conversely of ETR1:HA pulled down with PLS:GFP, compared with the same assay in the presence of the metal chelator 2 mM EDTA (Figure 4A, 4B, 4E, and 4F).

To investigate the specificity of PLS binding, synthetic PLS(FL) was introduced into infiltrated *N. benthamiana* leaves 30 min before tissue harvest in the presence of both copper to maximize PLS–ETR1 interaction and the proteasome inhibitor MG-132 to prevent protein degradation. The addition of 25 nM synthetic PLS caused an ~80% reduction in PLS:GFP binding to ETR1:HA (Figure 4C and 4D), suggesting that the synthetic PLS peptide competed for ETR1 binding, showing the specificity of PLS for ETR1. The anti-GFP beads bound two sizes of PLS-GFP protein (Figure 4E), both of which were larger than a GFP-only control, suggesting that the PLS peptide undergoes cleavage, a change in conformation, post-translational modification, or incomplete reduction of Cys residues on some PLS; similarly, free GFP was also seen in the input samples from *N. benthamiana* leaves (Figure 4A, inset panel). When ETR1:HA was used to pull down PLS-GFP, only the larger peptide was present (Figure 4A), suggesting that ETR1 binds a longer version of the PLS peptide.

To pinpoint the interaction site at the receptor in more detail, *in vitro* binding studies were performed with purified receptor variants and PLS by microscale thermophoresis (Figure 4G). Binding of PLS was observed only with receptor variants containing the N-terminal transmembrane domain. By contrast, no binding was detected with ETR1 that lacked this domain ($ETR1^{306-738}$). The terminal transmembrane domain harbors the ethylene and copper binding region (Schott-Verdugo et al., 2019).

PLS binds Cu(I)

Cysteine residues are common metal-ligand binding residues in low-molecular-weight copper-handling peptides, and predictions of PLS structure (supplemental Figure 4) suggest a single α-helix plus an unstructured region with two cysteines ($CX_{10}C$ arrangement, where X is any amino acid) with some analogy to copper-metallochaperones such as Cox17 or other CX_9C twin

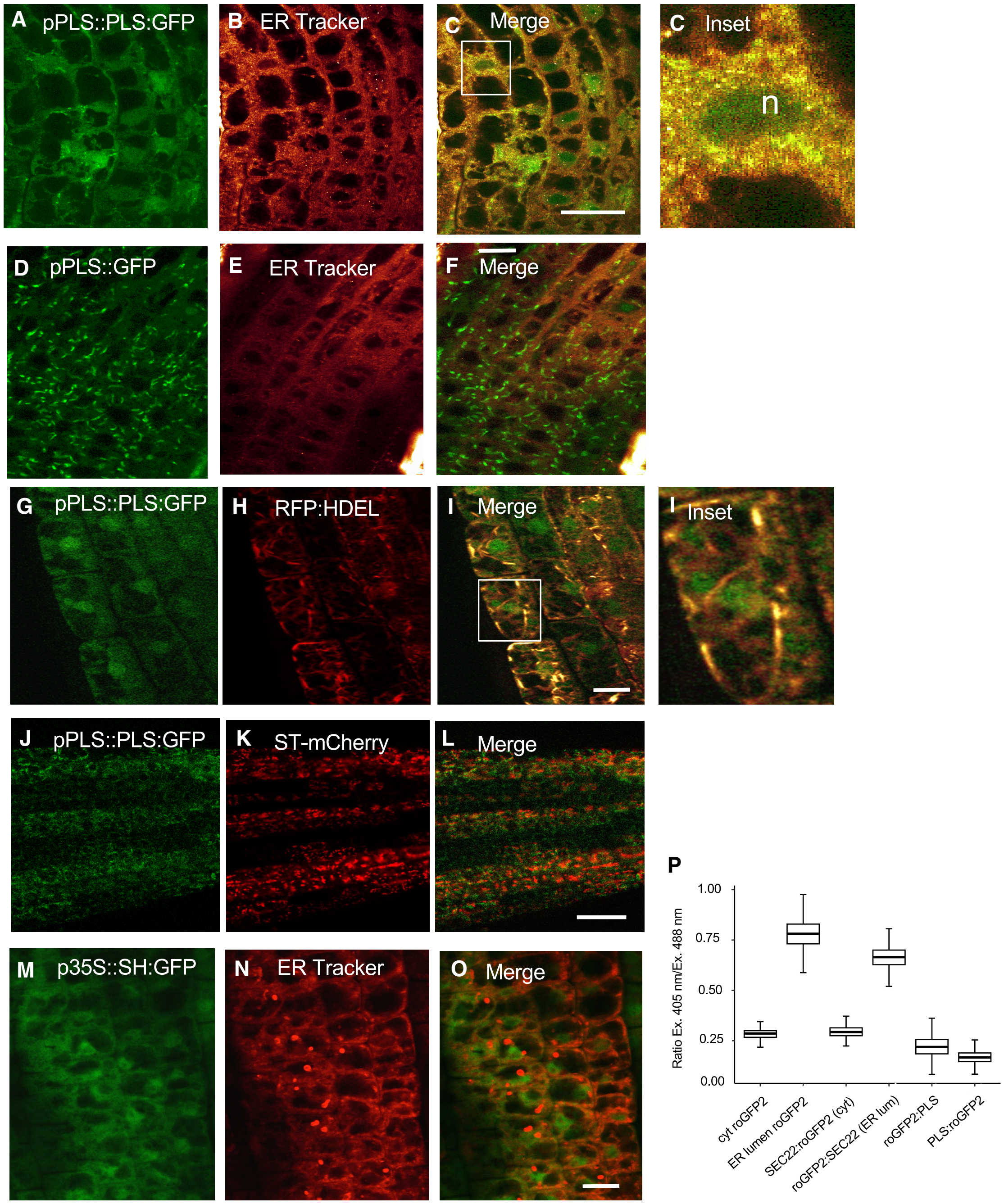

Figure 3. PLS localizes to the endoplasmic reticulum.

(A–O) PLS::PLS:GFP fusion protein localization in stably transformed *Arabidopsis* plants. **(A, G, and J)** PLS::PLS:GFP co-localizes with the endoplasmic reticulum markers ER Tracker **(B, C, and C** inset**)** and RFP:HDEL **(H, I, and I** inset**)**, but SH:GFP (which is cytoplasmically localized) does not **(D–F and M–O)**. PLS:GFP fluorescence is seen also in nuclei (n). PLS::PLS:GFP **(J)** does not co-localize with the *trans*-Golgi marker ST-mCherry **(K and L)**. Scale bars correspond to 25 μm **(C and L) and** 10 μm **(F, I, and O)**. Root epidermal cells in the transition zone were imaged. **(P)** Ratiometric analysis of roGFP2 fusion constructs transiently expressed in *N. benthamiana*. Comparison of the excitation ratios of PLS-roGFP2 and roGFP2-PLS with control constructs (free roGFP2, SEC22 fusions) reveals that PLS localizes at least predominantly to the cytosolic side of the ER.

proteins (Robinson and Winge, 2010; Yamasaki et al., 2009; Zhang and Li, 2013; Zhang et al., 2014). In view of both structural considerations and the copper dependency of ETR1, we examined whether the two cysteine residues (C6 and C17) in PLS play a functional role by analyzing both *pls* mutant complementation and copper binding by PLS peptide variants.

A mutated *Arabidopsis* full-length peptide in which both cysteines were replaced with serines, PLS(FL C6S, C17S), was non-functional in hydroponic root-feeding assays, failing to rescue the short primary root phenotype of the *pls* mutant (Figure 5A). Furthermore, as indicated above, the 9-amino-acid sequence N2 and the C-terminal sequences C1 and C2 from *Arabidopsis*

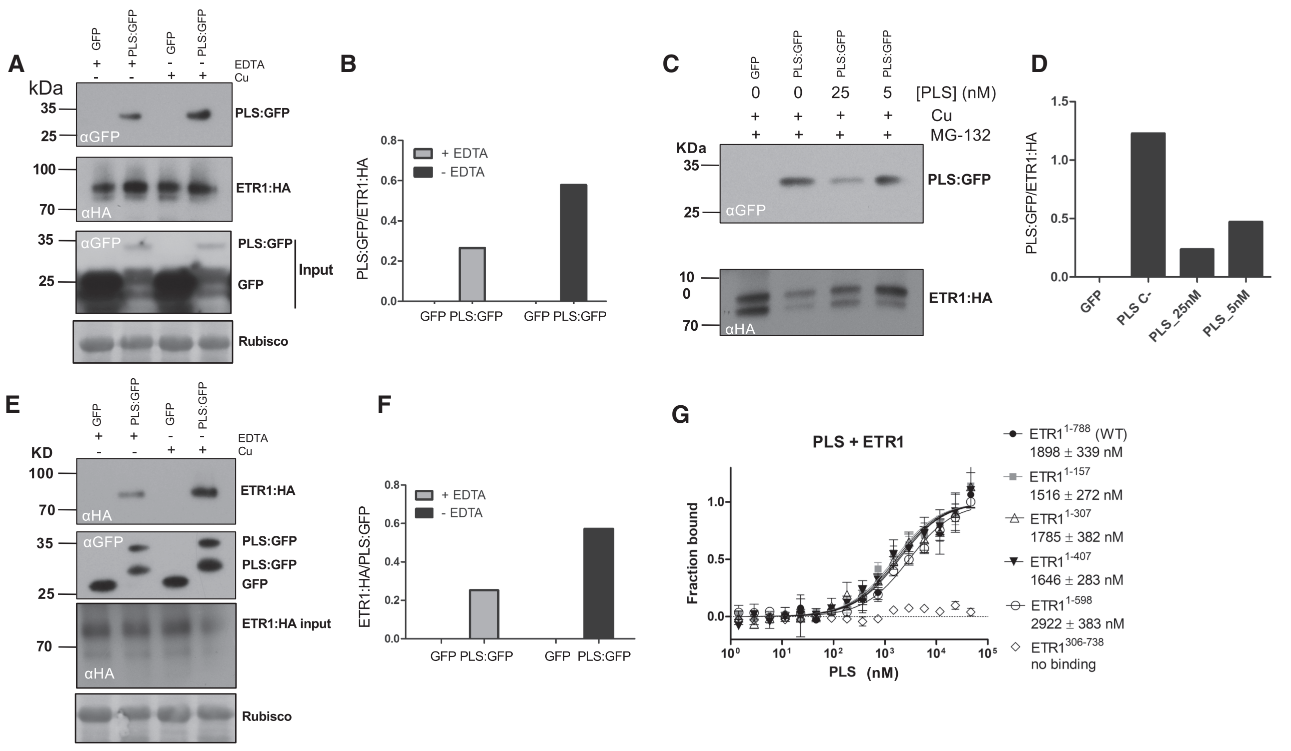

Figure 4. PLS interacts with the ethylene receptor ETR1.

(A) Co-immunoprecipitation of PLS:GFP by ETR1:HA (anti-HA beads, upper panel) in leaves of *N. benthamiana* in the presence and absence of 0.5 μM $CuSO_4$ and EDTA (to remove Cu). Lower panels show the presence of ETR1:HA in extracts using anti-HA antibody (αHA) and protein input blots using anti-GFP (αGFP) and anti-Rubisco (Rubisco) antibodies.

(B) Densitometry scan of immunoblot.

(C) Competition assay showing a reduced binding between PLS:GFP and ETR1:HA in the presence of 0, 5, or 25 nM PLS peptide, in the presence of 0.5 μM $CuSO_4$ and 50 μM MG-132, a proteasome inhibitor (upper panel). Lower panel shows ETR1:HA in extracts using anti-HA (αHA) antibody.

(D) Densitometry scan of immunoblot.

(E) Co-immunoprecipitation of ETR1:HA by PLS:GFP (anti-GFP beads, upper panel) or PLS:GFP and GFP using anti-GFP beads (second panel) in leaves of *N. benthamiana*, showing the effect of EDTA (to remove Cu) on the interaction between ETR1:HA and PLS:GFP. Lower two panels show the presence of ETR1:HA in protein input blots using anti-ETR1:HA (αHA) and anti-Rubisco (Rubisco) antibodies. αHA, anti-HA antibody beads; αGFP, anti-GFP antibody beads. Appropriate anti-HA, anti-GFP, and anti-Rubisco antibodies were used for protein visualization.

(F) Densitometry scan of immunoblot in **(E)**.

(G) *In vitro* microscale thermophoresis binding curves of different recombinant ETR1 truncations with PLS. Binding of PLS was observed with full-length ETR1 and all C-terminal truncations but not with $ETR1^{306-738}$, which lacked the N-terminal transmembrane part of the receptor. The values shown in the figure represent the binding constants of PLS with the indicated ETR1 constructs. Superscript numbers in the construct names denote amino acid positions within the protein. The values are shown with their respective standard deviations.

PLS, which each contain only one cysteine residue, were unable to rescue the mutant (Figure 2C). These results indicate that the two cysteine residues are required for biological activity, measured as primary root growth. Interestingly, RNA-seq data showed that 19 out of 287 genes associated with the GO term response to metal ion (GO:0010038) were also significantly downregulated in the *pls* mutant (supplemental Table 4, enrichment FDR = 0.000057). All 19 genes are associated with stress responses, including oxidative, salt, metal, and osmotic stress. For example, AT5G14545 (MIR598b) and AT4G25100 (encoding a Fe-superoxide dismutase) are both major targets of the Cu deficiency response regulator SQUAMOSA-PROMOTER BINDING PROTEIN-LIKE 7 (SP7) (Yamasaki et al., 2009; Zhang and Li., 2013; Zhang et al., 2014), which also interacts with and regulates RAN1 in ethylene signaling and is feedback-regulated by ethylene signaling (Yang et al., 2022). AT3G03780 encodes a methionine synthase (linked to ethylene biosynthesis), and AT3G56240 encodes the CCH copper chaperone (supplemental Table 8).

To determine a possible role for PLS and the cysteine residues in binding Cu(I), synthesized PLS peptide was titrated with Cu(I) ions under strictly anaerobic conditions and monitored for copper-dependent spectral features (Figure 5B–5D). Both the UV–vis (putative metal-to-ligand charge-transfer) and fluorescence (putative tyrosine solvent access) spectra of PLS changed as a function of Cu(I) ion concentration, consistent with complex formation (Figure 5C). We titrated PLS peptide against bicinchoninic acid (BCA) (Figure 5B), which is a chromophore that binds Cu(I) (Xiao et al., 2011; Figure 5D and supplemental Figures 8 and 9). The rationale was to determine whether PLS could compete for Cu(I) with BCA, which would be indicative of binding by PLS. PLS peptide was titrated with Cu(I) in the presence of 87 μM BCA. The presence of PLS peptide (44.8 μM) increased the

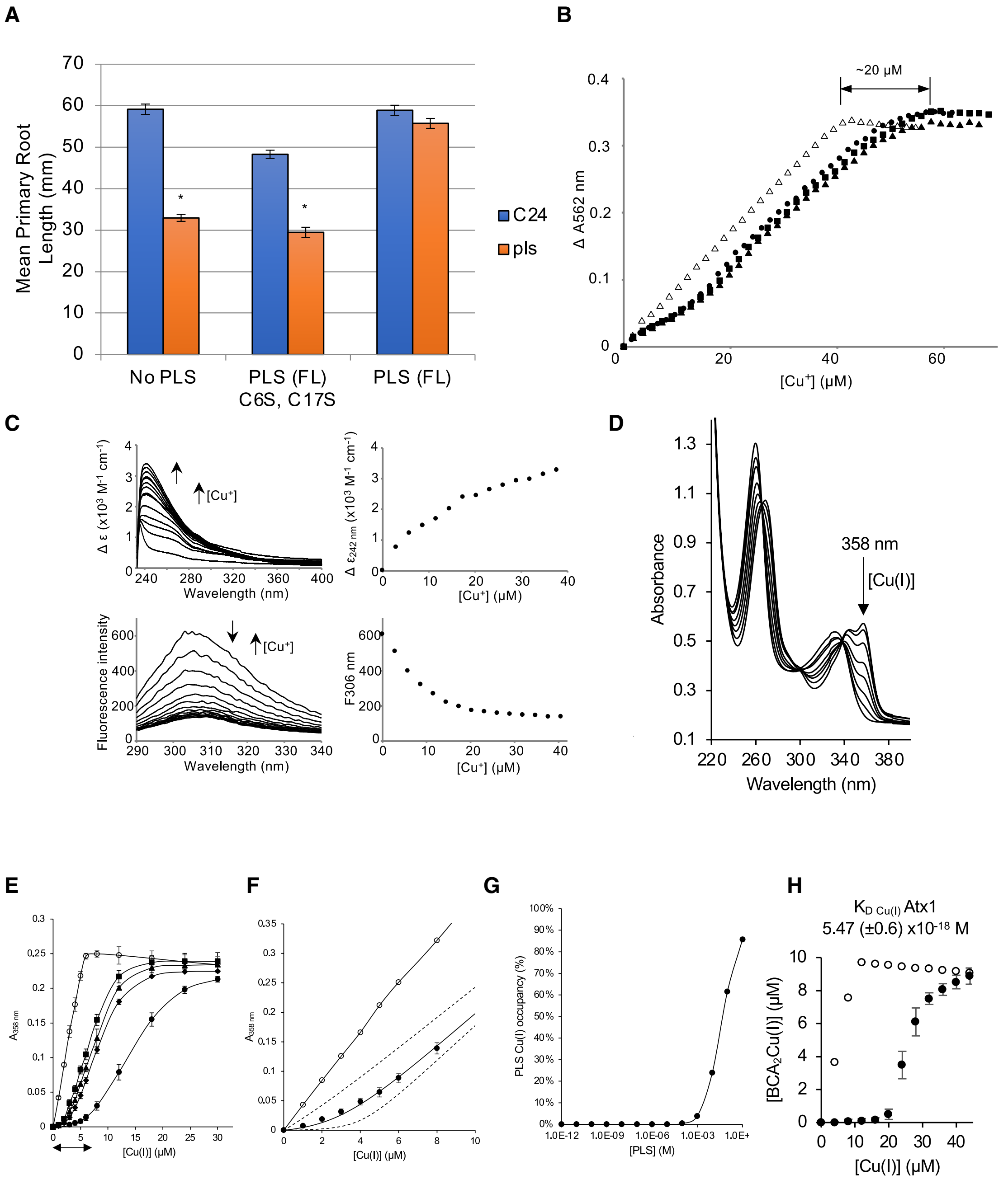

Figure 5. PLS binds copper.

(A) C6S and C17S are required for PLS function. Seedlings were grown hydroponically for 10 days in the presence (100 nM) or absence of peptide (FL or C6S, C17S). Blue bars are the wild type (C24), and orange bars are *pls*. $*P < 0.05$, *t*-test between C24 and *pls*, $n = 25$. Error bars show ±1 standard error, $n = 14$.

(B) Bicinchoninic acid (BCA) absorbance in the presence of synthetic PLS (44.8 μM, filled symbols) or an equivalent volume (to PLS) of DMSO (open symbols). Experiments with PLS used 88.3 μM (circles), 84 μM (triangles), and 88.4 μM (squares) BCA. The control experiment with DMSO used 85.3 μM BCA.

(C) UV–vis apo subtracted difference spectra of synthetic PLS (19.8 μM) upon titration with CuCl (top left), and binding isotherm of the feature at 242 nm (top right). Fluorescence spectra of PLS (19.8 μM) upon titration with CuCl (bottom left), and binding isotherm of the feature at 306 nm (bottom right). The first arrow indicates an increase or decrease in intensity (upward or downward, respectively) with increasing (second upward arrow) addition of Cu(I).

(D) Absorbance of BCA (17.3 μM) titrated with Cu(I) (representative spectrum, $n = 2$, full data in supplemental Figure 8).

(E) Binding isotherms ($A_{358\ nm}$) of BCA (10 μM) in the presence/absence (filled/empty symbols) of 14 μM MBP:PLS (circles) or MBP:PLS mutants (C6S, C17S, and C6S/C17S: triangles, diamonds, and squares, respectively) titrated with Cu(I) ($n = 3$, ±SD). Arrow indicates ∼7 μM withholding of Cu(I) from BCA.

(legend continued on next page)

concentration of Cu(I) required to saturate BCA by ~20 μM (Figure 5B), and the initial gradient was shallower than in the control reaction, which contained BCA and Cu(I) but lacked PLS. Thus, PLS withholds Cu(I) from BCA, implying that its affinity for Cu(I) (K_{Cu} PLS) is tighter than that of BCA (K_{Cu} BCA); these data also support a 2:1 stoichiometry of binding to the tightest site. Titration experiments also showed that although synthetic PLS(FL) withholds Cu(I) from BCA, mutant PLS peptide (FL C6S, C17S) does not (supplemental Figure 10), confirming a role for the cysteine residues in Cu(I) binding. Because problems with PLS solubility could potentially reduce the accuracy with which we determined the stoichiometry and affinity for Cu(I) (complete solubility is required for precise affinity quantification), we also tested a version of PLS fused to maltose binding protein (MBP:PLS), which retained solubility when titrated with Cu(I) ions under strictly anaerobic conditions. MBP:PLS (14 μM) was found to withhold ~7 μM Cu(I) from BCA, again indicative of a 2:1 PLS:Cu(I) stoichiometry, and tight binding was cysteine dependent (Figure 5E). A β_2 affinity of 3.79 (±1.5) × 10^{19} M^{-2} was determined by competition against an excess of BCA, and the fit significantly departed from simulations 10× tighter or weaker (Figure 5F and supplemental Figure 11).

Metal binding in biology is challenging to predict because the formation of metal-protein complexes is a combined function of metal affinity for a given protein and metal availability, which would need to be known for Cu(I) in the *Arabidopsis* cytosol in the case of PLS (assuming the same orientation of binding residues as with the GFP tag; Figure 3P). Cu(I) occupancy of the cytosolic copper chaperone ANTIOXIDANT PROTEIN 1 (ATX1) tracks fluctuations in available cytosolic Cu(I) such that its affinity approximates to the mid-point of the range of Cu(I) availabilities within this eukaryotic compartment (Yu et al., 2017; Morgan et al., 2019). *Arabidopsis* ATX1 was therefore expressed and purified to determine a 1:1 ATX1:Cu (I) stoichiometry and an affinity $K_{D\ Cu(I)}$ of 5.47 (±0.6) × 10^{-18} M (Figure 5H and supplemental Figure 12). The mid-point availability was thus estimated to be 5.47 (±0.6) × 10^{-18} M, noting that this number informs how tightly (as an activity) a labile, ligand-exchangeable pool is bound rather than some negligible concentration of hydrated Cu(I) (for simplicity, we have not calculated free energies for complex formation here). Figure 5F and 5G reveal that the cytosolic concentration of PLS would need to exceed (improbable) millimolar concentrations for Cu(I)-dependent homodimers to form at this cytosolic Cu(I) availability (mathematical models and equations are shown in supplemental text). It is thus unlikely that the Cu(I):PLS_2 complex alone delivers Cu(I) to, or retrieves it from, the interacting cuproprotein ETR1. Cu(I)-dependent PLS–ETR1 heterodimeric complexes are the more likely functional species, and we conclude that PLS binding alters either ETR1 conformation or ethylene availability to regulate receptor activity.

DISCUSSION

We present evidence for a role of the PLS peptide as a new Cu(I)-binding peptide that, on the basis of its physical interactions with the receptor and its ethylene-signaling function as revealed by genetic and physiological experiments, acts as a regulatory component of the ethylene signaling pathway. Peptides can act as ligands for receptor kinases to regulate signaling pathways, such as in plant immunity (Campos et al., 2018), development (Willoughby and Nimchuk, 2021), or responses to abiotic stresses (Kim et al., 2021). We propose that the PLS peptide is a new component of the regulatory mechanism for ethylene receptor function linked to its copper-binding activity (Rodriguez et al., 1999). Loss of copper binding through mutation of the two cysteines in PLS ablates its biological activity (Figures 2C, 5A, and 5E; supplemental Figure 10). The *pls* mutant phenotype has some similarities (enhanced ethylene responses) to that of strong *ran1* mutant alleles in which copper delivery to the receptor is compromised (Binder et al., 2010). It also shares (statistically significant) overlap in differentially expressed genes (DEGs) with the *ctr1* mutant: 11.9% of the *ctr1* upregulated DEGs are shared with *pls* (each compared with its respective wild type; supplemental Figure 13).

Compared with the wild type, the *pls* mutant shows significant upregulation of genes associated with diverse GO terms that include responses to biotic and abiotic stimuli and plant immunity, which in turn are regulated by hormonal systems that include ethylene signaling. However, an intriguing feature of *PLS* overexpression is that there was no significant downregulation of these gene categories to levels below those seen in the wild type. Rather, their expression returned to wild-type levels but was not repressed further. For example, although *ESE3* was upregulated in the *pls* mutant and downregulated in PLSOx seedlings, other ethylene-responsive genes were either upregulated in both genotypes or restored to wild-type levels in PLSOx (supplemental Tables 1, 2, 3, and 4). This suggests that *PLS* overexpression does not have a significant additional biological effect beyond restoration to wild-type levels, consistent with the lack of a strong seedling phenotype in light-grown PLSOx seedlings (although they do show reduced sensitivity to ACC inhibition of root growth; Casson et al., 2002; Chilley et al., 2006). One possible interpretation is that saturation of the PLS–receptor interaction occurs over a small concentration range of the PLS peptide. It is also likely that crosstalk between ethylene and other signaling pathways influences the output of these interactions when measured as gene expression levels or patterns (Moore et al., 2024), and so some pleiotropic effects of the *pls* mutation may be seen.

Many plant peptides involved in signaling are processed from longer pre-proteins (Olsson et al., 2019). By contrast, PLS is translated from an open reading frame to produce a functional 36-amino-acid peptide; the peptide appears to be cleaved or

(F) Binding isotherms ($A_{358\ nm}$) of BCA (50 μM) in the presence/absence of 10 μM MBP:PLS (filled/empty symbols) titrated with Cu(I). Model (solid line) describes Cu(I) binding as a 2:1 complex, with a β_2 affinity of 3.79 (±1.5) × 10^{19} M^{-2}. Dotted lines simulate 10× weaker or tighter affinity (n = 3, ±SD).

(G) Simulated Cu(I) occupancy (calculated as described in the supplemental text) as a function of [PLS] using a β_2 Cu(I) affinity of 3.79 × 10^{19} M^{-2} and conditions matching Cu(I) availability at 50% ATX1 saturation from a determined KD Cu(I) ATX1 (supplemental Figure 12).

(H) *Arabidopsis* ATX1 (20 μM, filled circles) withholds one Cu(I) equivalent from 20 μM BCA (open circles, BCA alone) (n = 3, ±SD), with K_D Cu(I) 5.47 × 10^{-18} M (supplemental Figure 12).

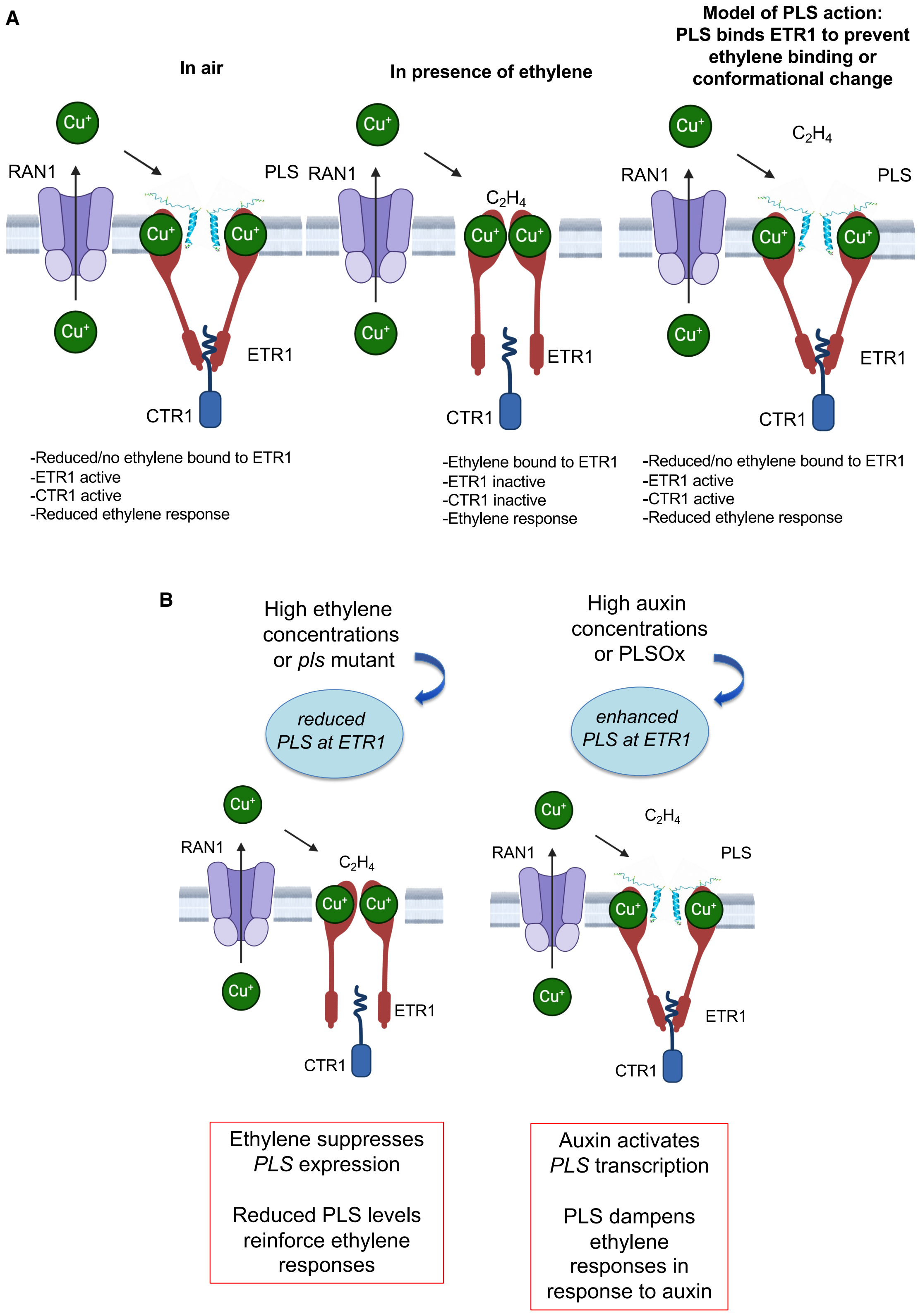

Figure 6. Model of the role of PLS in ethylene signaling.

In this model, we assume that RAN1 and ETR1 are in the same membranous compartment (likely the ER), at least transiently.

(A) Model of PLS interaction with ETR1. RAN1 is believed to pump Cu(I) into the ER lumen and then to ETR1. In air (absence of ethylene; left panel), the receptor complex is active and activates CTR1, a negative regulator of ethylene responses, and PLS likely binds the receptor. In the presence of ethylene

(legend continued on next page)

otherwise modified in the cell (Figure 4E). It is possible that the three arginines (amino acids 10–12) may represent a cleavage site (Duckert et al., 2004; Capraro et al., 2015), which would produce the observed shorter GFP fusion protein (∼30 kDa) detected in anti-GFP CoIP blots (Figure 4E). The size of this PLS:GFP cleavage product suggests that this peptide fragment represents GFP (∼27 kDa) plus a 3 kDa fragment of the C-terminal region of PLS. Such cleavage would likely inactivate PLS function, as we found that shorter N- and C-terminal fragments (each with only one cysteine residue) were not biologically active (Figure 2C). PLS:GFP localizes to several subcellular compartments (although not to the *trans*-Golgi), including the ER, where the ethylene receptor is found (Figure 3). Furthermore, PLS can interact directly with the receptor protein ETR1 (and specifically with the N-terminal copper-binding transmembrane domain of ETR1).

Previously, a model was proposed for copper delivery into the cell via the COPT family of transporters, which are located at the root plasma membrane (Chen et al., 2022), to RAN1 via ATX1 and/or CCH (Li et al., 2017; Hoppen et al., 2019). *Arabidopsis* has three known copper chaperones, ATX1, CCH, and a copper chaperone for superoxide dismutase, CCS (Casareno et al., 1998; Chu et al., 2005; Puig et al., 2007). These are required for copper homeostasis, and both ATX1 and CCH have been shown to interact with RAN1 (Li et al., 2017), providing a link with ethylene signaling (Andrés-Colás et al., 2006; Puig et al., 2007). Chaperones are required for the transport of reactive copper to the correct compartment via these chaperones, avoiding cytotoxicity, after import into the cell; and into the xylem from the cytosol via HMA5, which is structurally similar to RAN1 (Williams and Mills, 2005; Burkhead et al., 2009). The loss-of-function mutants *cch* and *atx1* have no abnormal phenotype when grown under standard conditions, but *atx1* and the *atx1 cch* double mutant, although not the *cch* single mutant, are hypersensitive to exogenous copper (Shin et al., 2012). The functions of ATX1 and CCH have likely diverged, a view supported by their differential transcriptional regulation by copper and different cell-type specificities (Shin et al., 2012). The *ran1* mutant, however, is not copper hypersensitive, whereas *hma5* is; and the *pls* mutant, like *ran1*, is not copper hypersensitive (supplemental Figure 14), suggesting that its role is distinct from that of ATX1 or CCH. Interestingly, however, CCH is downregulated in the *pls* mutant (supplemental Table 8), suggesting some functional interaction between PLS and CCH. However, given its predicted low concentration in the cell and its 2:1 stoichiometry with Cu(I) (Figure 5F and 5G and supplemental Figures 11 and 12), we conclude that PLS is unlikely to act as a cytosolic copper chaperone.

A lack of copper delivery in the strong *ran1-3* and *ran1-4* null alleles causes constitutive ethylene responses (Woeste and Kieber, 2000), producing a triple-response phenotype similar to that of the *pls* mutant (Casson et al., 2002). These *ran1* mutants fail to bind ethylene owing to a lack of copper at the ethylene binding site (Binder et al., 2010). The decrease in ethylene binding in the *ran1-3* and *ran1-4* mutants is not due to reduced levels of ETR1 protein (Binder et al., 2010), and regulation of the ethylene receptor ETR1 is generally regarded to be independent from *ETR1* transcription or degradation (Hua et al., 1998). Weaker *ran1-1* and *ran1-2* alleles show an ethylene response phenotype only if they are grown in the presence of copper chelators (Binder et al., 2010). Therefore, defective RAN1 results in defective copper delivery to wild-type receptors, generating non-functional receptors that cannot interact with CTR1, resulting in downstream ethylene responses.

Intriguingly, the *ran1-3* and *ran1-4* mutants with constitutive ethylene responses are distinct from the ethylene-insensitive *etr1-1* mutant, which also cannot bind copper. Therefore, both the *etr1-1* mutant receptor and the strong *ran1* mutant alleles have reduced ethylene binding ability, but the *etr1-1* mutation, which is a gain-of-function mutation, produces seedling phenotypes very different from those of seedlings carrying the ethylene-hypersensitive wild-type receptors, which simply lack copper because of defective delivery (in *ran1-3* and *ran1-4).* A receptor carrying the *etr1-1* mutation is maintained in its "active" state and constitutively inhibits downstream ethylene responses, whereas in the *ran1* mutants, ETR1 remains in the "inactive" state, promoting ethylene responses (Binder et al., 2010). Those authors therefore proposed that copper has more than one role in the regulation of ETR1 and ethylene signaling: it is crucial for binding ethylene molecules but is also needed for the process of signal transduction by the receptor protein, through an unknown mechanism, to regulate downstream ethylene responses.

We propose that PLS represents a new component of this pathway, providing some tissue specificity to ethylene receptor activity. Given the low probability that PLS acts as a cytosolic copper chaperone, it is more likely to regulate ethylene responses via its demonstrated Cu-dependent ETR1 binding to induce the active state of the receptor, with consequent inhibition of ethylene responses. The RFP:HDEL and roGFP2 co-localization experiments indicated the presence of PLS at the cytosolic side of the ER, in the ER lumen, and binding with the ETR1 transmembrane domain (as well as other compartments; Figure 3). Given that the PLS fusion proteins may not have mimicked native PLS localization perfectly, we cannot exclude the possibility that

(center panel), the receptor is in an inactive state and cannot activate CTR1, resulting in ethylene responses. In the model for PLS function, PLS binds to ETR1 (with binding potentially enhanced by Cu(I) in the ETR1 transmembrane domain) and (as in the left panel) affects the receptor conformation, potentially blocking ethylene binding to ETR1 or inducing some other conformational change, leading to active ETR1, active CTR1, and reduced ethylene signaling. This model is consistent with the enhanced ethylene responses observed in the *pls* loss-of-function mutant.

(B) Model for the regulation of ethylene responses by auxin and ethylene via effects on *PLS* expression. Left: under high-ethylene conditions or in the *pls* mutant, ETR1 and CTR1 are inactive, leading to enhanced ethylene responses. Right: under high-auxin conditions or in PLS-overexpressing plants, *PLS* transcription is relatively high, and PLS binds to the receptor to disrupt ethylene binding in an otherwise inactive ETR1 receptor (with ethylene responses activated), thus reducing ethylene responses. In, for example, a root tip with relatively high auxin concentrations, *PLS* expression would suppress ethylene responses to allow root growth; but under high-ethylene conditions, root growth would be inhibited (as seen in the *pls* mutant in the absence of high ethylene levels).

PLS is present at both the cytosolic and luminal domains of the ER, or indeed in the membrane.

The *pls* mutant is rescued by the supply of silver ions (Casson et al., 2002), and silver is likely delivered to the ethylene receptor via RAN1 (Binder et al., 2010); these findings argue against a mechanism in which PLS acts downstream of RAN1 to metalate ETR1 in the ER lumen. It is also unlikely that PLS removes Cu(I) from the receptor, as this would inactivate the receptor and lead to enhanced ethylene signaling in the presence of PLS, which is the opposite of what would be expected from the *pls* mutant phenotype. We therefore favor a model (Figure 6) in which PLS binds ETR1 as part of the receptor biogenesis mechanism, with the PLS–ETR1 physical interaction enhanced in the presence of copper (Figure 4). We propose that this interaction regulates receptor conformation to generate the active form in which ethylene responses are repressed through promotion of receptor interaction with CTR1. This may occur through regulation of Cu(I) homeostasis at the receptor or via other structural interactions; for example, PLS could physically block ethylene binding at the Cu(I) pocket. This question can form the basis of future investigations.

Given that *PLS* is predominantly expressed in specific tissues, notably root tip and leaf vascular tissues (Casson et al., 2002), it seems likely that PLS adds a level of regulation of receptor function in specific tissues, with a focus in this paper on the root (although developmental defects in vascular patterning of the leaf are also observed in the *pls* mutant; Casson et al., 2002). The relatively spatially restricted pattern of *PLS* expression might account for the less strong ethylene hypersignaling phenotype of the *pls* mutant compared with, for example, the strong *ran1* or *ctr1* mutants, although there are similarities, as described above. We propose that PLS acts to dampen ethylene responses at sites of high auxin concentration. *PLS* transcription is strongly upregulated by auxin, and its expression profile is similar to that of the auxin reporter DR5 (Sabatini et al., 1999; Casson et al., 2002). The root tip, where *PLS* is most strongly expressed, is a site of high auxin concentration, whereby an auxin maximum is formed around the stem cell niche to regulate both stem cell identity and function (Sabatini et al., 1999). The vascular tissues are also a site of high auxin concentration (Bishopp et al., 2011). Auxin can induce ethylene biosynthesis by upregulation of the ethylene biosynthetic enzyme ACC synthase (Abel et al., 1995), and high ethylene concentrations can lead to both reduced meristem function and aberrant division of the quiescent center and cells within the stem cell niche and columella (Ortega-Martinez et al., 2007). Therefore, ethylene biosynthesis and signaling in the root tip must be tightly regulated to allow growth control. *PLS* expression is transcriptionally activated by auxin and suppressed by ethylene. We have shown in previous work that there is crosstalk among PLS, ethylene, and other signaling pathways, notably those of auxin, cytokinin, and ABA, in the root (e.g., Liu et al., 2010; 2013; Moore et al., 2015; 2024), and the distinctiveness of *pls* compared with other ethylene signaling mutants may be linked to its restricted expression pattern. This network provides a mechanism to suppress growth-inhibitory ethylene responses in the high-auxin environment of the root tip, which is dependent on the tissue-specific expression of *PLS* (Liu et al., 2010; Figure 6B and supplemental Figure 15) and is mediated through its interaction with the ethylene receptor.

Future structural studies should reveal more about the PLS–ETR1 interaction and the diverse roles of copper ions in ethylene signal transduction, which represents a new paradigm for the regulation of signaling-protein function by metals.

METHODS

Plant material

proPLS::PLS:GFP comprises a 1.5-kb *PLS* promoter sequence (Casson et al., 2002) fused to the *PLS* open reading frame in pMDC107 (obtained from Prof. P.J. Hussey, Durham University) as a C-terminal GFP translational fusion. The *PLS* ORF with its 1.5-kb promoter was amplified from the TOPO 2.1 vector, which contained the *PLS* gene sequence with promoter (Casson et al., 2002); the primers used for cloning are listed in supplemental Table 9. *pls* mutant plants were transformed using the method of Clough and Bent (1998). *proPLS::GFP* plants were generated using the 1.5-kb *PLS* promoter (Casson et al., 2002) fused to *GFP* in a wild-type background. *p35S::GFP* seedlings were obtained from Dr. Piers Hemsley, Durham University. Seedlings of *Arabidopsis thaliana* ecotype C24 or Col-0, *pls* mutants, and transgenic *PLS* overexpressers, all from lab stocks, were grown on solid sterile half-strength (2.2 g/l) Murashige and Skoog medium (Sigma-Aldrich) containing 10 g/l sucrose (half-strength [½] MS10 medium) solidified with 2.5 g/l Phytagel (Sigma-Aldrich) in 90-mm Petri dishes (Sarstedt, Leicester, UK) at 21°C under a 16-h photoperiod as described previously (Casson et al., 2002; Chilley et al., 2006). *N. benthamiana* plants (lab stocks) were grown in a controlled environment (21°C, 16-h photoperiod) for transient expression studies in leaf tissue. For hydroponic feeding studies, *Arabidopsis* seedlings were cultured in liquid ½ MS10 medium (1 ml/well) in sterile 24-well plates (Sarstedt), essentially as described in Matsuzaki et al. (2010). For hormone and peptide assays, one seedling was grown in each well at 21°C under a 16-h photoperiod. For peptide feeding experiments, purified freeze-dried peptide was dissolved in DMSO to create a 500-μM stock solution. The peptide stock solution was added to liquid ½ MS10 medium containing 0.1% DMSO to obtain a final peptide concentration of 50 or 100 nM (or 10, 25, 50, and 100 nM for dose-dependent assays). For copper treatments, 1 mM $CuSO_4$ solution was filter sterilized and added to autoclaved liquid ½ MS10 medium to obtain final $CuSO_4$ concentrations of 0, 5, 10, 15, 20, 25, 30, 35, 40, 45, and 50 μM. Seedlings were scanned to create a digital image, and root lengths of the seedlings were measured using ImageJ. Statistical analysis was performed using Real Statistics Resource Pack software (release 3.8, www.real-statistics.com) in Excel (Microsoft) using data from at least 10 independently grown seedlings for root growth analysis; specific numbers are defined for each experiment.

Peptide synthesis

Peptides were either obtained from Cambridge Research Biochemicals (Billingham, UK) or synthesized in the laboratory by Fmoc solid-phase peptide synthesis on a CEM Liberty1 single-channel peptide synthesizer equipped with a Discover microwave unit. Reactions were performed in a 30-ml PTFE reaction vessel with microwave heating FW:EpiLife Media and agitation by bubbling nitrogen. Peptide synthesis was performed using 2-chlorotrityl chloride resin (0.122 mmol g^{-1}, Novabiochem) using Fmoc-protected amino acids. The first amino acid residue at the C terminus (histidine) was coupled manually by mixing 76 mg (1 eq.) Fmoc-His (Trt)-OH, 0.09 ml (4 eq.) *N,N*-diisopropylethylamine (DIPEA), 1 ml dichloromethane (DCM), and 1 ml dimethylformamide (DMF) until the amino acid powder had dissolved. The mixture was added to 0.1 mmol resin and stirred gently for 120 min at room temperature. Resin was washed with 3× DCM/MeOH/DIPEA (17:2:1), 3× DCM, 2× DMF, and 2× DCM. Amino acid coupling reactions were performed using Fmoc-protected amino acids present in five-fold excess (2 M concentration), HOBt (0.5 M HOBt

in DMF, used at the activator position), and DIC (0.8 M in DMSO, used at the activator base position). For double and triple couplings, the reaction vessel was drained after each coupling cycle, and fresh reagents were added. Before each coupling, a room temperature pre-activation period of 1–2 h was used. Microwave-assisted couplings were performed for 10 min at 75°C at 25 W power. Cys and His residues were coupled at low temperature (10 min at room temperature followed by 10 min at 50°C, 25 W). Arg residues were double coupled, first for 45 min at room temperature plus 5 min at 75°C (25 W) and then with the standard microwave conditions above. Fmoc groups were removed by two piperidine solution treatments (20% piperidine in DMF) in succession: 5 min, then 10 min. The peptide was cleaved from the resin using 3 ml 95% TFA in dH_2O/TIPS (2.85 ml TFA, 0.15 ml dH_2O, 0.15 ml triisopropylsilane), then dissolved in water with a small volume of MeCN and lyophilized to produce a powder using a Christ ALPHA 1-2 LDplus freeze dryer. The PLS truncation N1 (Figure 2A) was tagged at the N terminus with a 5-FAM fluorescent tag to enable detection of peptide uptake. The 5-FAM molecule contains a carboxylic acid moiety that can be attached to the N-terminal primary amine and is excited at 488 nm. The remainder of the molecule is planar, with four 6-carbon rings and a chemical formula of $C_{21}H_{12}O_7$. Because of the large size of 5-FAM, it was attached to the shorter PLS(N1) peptide rather than the full-length PLS to avoid the possibility that the latter construct was too large for root uptake.

Preparative HPLC

Peptide products were analyzed and purified by high-performance liquid chromatography (HPLC) at 280 nm. Freeze-dried peptide sample (25–50 mg) was dissolved in 1 ml 1:1 H_2O:MeCN and injected onto a Speck and Burke Analytical C18 column (5.0 μm, 10.0 × 250 mm) attached to a PerkinElmer (MA) Series 200 LC Pump and a 785A UV/Vis Detector. Separation was achieved by a gradient elution of 10%–80% solvent B (solvent A = 0.08% TFA in water; solvent B = 0.08% TFA in acetonitrile) over 60 min, followed by 80%–100% B over 10 min, with a flow rate of 2 ml/min. Selected peptide fractions were lyophilized and a mass assigned using MALDI-TOF MS. Peptide sequences were identified by MALDI-TOF MS using an Autoflex II ToF/ToF mass spectrometer (Bruker Daltonik, Germany) equipped with a 337-nm nitrogen laser. MS data were processed using FlexAnalysis 2.0 (Bruker Daltonik).

Imaging

CSLM images for propidium iodide and GFP were obtained using a Leica SP5 TCS confocal microscope as described previously (Rowe et al., 2016). *proPLS::PLS:GFP*, *proPLS::GFP*, and *pro35S::GFP* seedlings were grown for 7 days on Phytagel ½ MS10 medium before ~25 mm of the root tip was removed and mounted in dH_2O prior to imaging. The ER marker *pro35S::RFP:HDEL* (Lee et al., 2013) (provided by Dr. Pengwei Wang, Durham University), and the *trans*-Golgi apparatus marker *proFGC-ST:mCherry* (from Nottingham Arabidopsis Stock Centre, www.arabidopsis.info) were introduced into *proPLS::PLS:GFP* plants by the floral dip method of transformation (Clough and Bent, 1998) using *A. tumefaciens* GV3101. The ER was also visualized using ER Tracker Red (ThermoFisher). Seven-day-old seedlings were stained for 30 min in the dark in liquid ½ MS10 medium containing 1 μM ER Tracker Red. Fluorophores were excited by the following lasers: 405 nm UV (for ethidium bromide), 488 nm 20 mW argon (for all GFP, 5-FAM, and acridine orange experiments), 543 nm 1.2 mW HeNe (for propidium iodide, RFP, and mCherry fluorophores), and 594 nm 2 mW HeNe (for ER Tracker). For standard photomultiplier tubes, a laser power of 21% and a smart gain of 800–1000 mV were used, depending on the intensity of the fluorescence. The HyD detector was used at 70%–120%.

Ratiometric analysis of roGFP2 fusion proteins

N- and C-terminal roGFP2 fusion proteins of PLS under the control of the CaMV35S promoter were generated by Gateway cloning using the pENTR/D-TOPO Cloning Kit (Invitrogen) as described previously (Hoppen et al., 2019). Infiltration and transient expression of roGFP2 fusions and control proteins were carried out as described in Brach et al. (2009). Image acquisition and data analysis were carried out as described by Hoppen et al. (2019). A minimum of 10 leaf optical sections were imaged and used for ratiometric analysis of the redox sensitive excitation properties of roGFP2.

RNA isolation, RNA-seq, and RT–qPCR

RNA was extracted from 7-day-old seedlings grown on ½ MS10 medium essentially as described previously (Thompson et al., 2023). The Illumina HiSeq 2500 system was used for RNA-seq of three biological replicate samples; libraries were prepared using the Illumina TruSeq Stranded Total RNA with Ribo-Zero Plant Sample Preparation kit (RS-122-2401) essentially as described in Thompson et al. (2023). Library quality control was carried out again using a TapeStation with D1000 ScreenTape (cat. no. 50675582). RNA-seq data were aligned to the TAIR10 genome sequence (EnsemblPlants, release 58) with the corresponding gtf file using STAR (v 2.7.11a; Dobin et al., 2013) to obtain a read count per gene. Read count data were analyzed using DESeq2 v 1.40.2 (Love et al., 2014) to obtain P values, adjusted P values, and $\log_2$ fold changes. DEGs were identified using the criterion of adjusted $P < 0.05$. GO analysis was performed using AgriGO (Tian et al., 2017) singular enrichment analysis. Gene expression heatmaps were generated from $\log_2$ normalized counts using pheatmap (v 1.0.12, https://cran.r-project.org/web/packages/pheatmap/index.html) with row scaling. RNA-seq data were deposited in GEO (https://www.ncbi.nlm.nih.gov/geo/) with accession number GSE256166 (Mudge et al., 2024).

For RT–qPCR, RNA was extracted from 7-day-old seedlings (3 biological replicates, 20 mg of tissue per replicate) as described previously (Thompson et al., 2023). Samples were checked for the presence of genomic DNA by PCR with the *ACTIN2* primers ACT2 forward and reverse. Primer sequences were determined using Primer-BLAST (https://www.ncbi.nlm.nih.gov/tools/primer-blast/). For each cDNA sample, transcript abundance of a gene of interest was quantified in relation to a stably expressed internal reference "housekeeping" gene, with three technical replicates. This allows the determination of fold-differences in the expression of the gene of interest that can be attributed to the treatment of the sample and corrects for variation between samples, such as differences in the quality or quantity of the extracted RNA. The data were analyzed by comparative quantitation using Rotor-Gene software. Mean transcript abundance was calculated from relative transcript abundance for each biological sample and represented graphically. Error bars show the upper and lower limits of the standard error of the mean. Primers are listed in supplemental Table 9.

Protein–protein interaction studies

Yeast 2-hybrid

The GAL4 2-hybrid phagemid vector system was used to detect protein–protein interactions *in vivo* in yeast, using the reporter genes β-galactosidase *(lacZ)* and histidine (*HIS3*) in the YRG-2 yeast strain, essentially as described previously (Zhong et al., 2008). DNA sequences encoding the target (ETR1) and bait (PLS) were inserted into the pAD-GAL4-2.1 A and pBD-GAL4 Cam phagemid vectors, respectively, and expressed as hybrid proteins. The hybrid proteins were then assayed for protein–protein interaction.

DNA sequences encoding the target and bait proteins were prepared by PCR amplification using primers designed specifically for the target (ETR1) and bait (PLS). Each set of primers contained specific endonuclease recognition sites on the ends corresponding to the restriction sites in the MCS of the pAD-GAL4-2.1 A and pBD-GAL4 Cam phagemid vectors. The DNA constructs for the target (ETR1) and bait (PLS) with specific restriction sites on the ends were then transformed into the TOPO 2.1 vector, and the sequence of the amplified DNA was confirmed by sequencing with M13 forward (CTG GCC GTC GTT TTA C) and M13 reverse (CAG GAA ACA GCT ATG AC) primers. The two vectors, pAD-GAL4-2.1 and

pBD-GAL4 Cam, were digested using specific restriction endonucleases and dephosphorylated prior to ligating the insert DNA. The DNA sequences encoding the target (ETR1) and bait (PLS) were then ligated into the same reading frame as the GAL4 AD of the pAD-GAL4-2.1 phagemid vector and the GAL4 BD of the pBD-GAL4 Cam phagemid vector. The primers used to clone ETR1 and PLS are found in supplemental Table 9.

The pGAL4 control plasmid was used alone to verify that induction of the *lacZ* and *HIS3* genes occurred and that the gene products were detectable. The pLamin C control plasmid was used in pairwise combination with the pAD-wild-type control plasmid or the pAD-MUT control plasmid to verify that the *lacZ* and *HIS3* genes were not induced, as the proteins expressed by each of these pairs do not interact *in vivo*.

Control plasmids were transformed into the YRG-2 strain prior to the initial transformation of the bait and target plasmids and used separately or in pairwise combinations for transformation of the YRG-2 yeast strain. Yeast competent cells were co-transformed with the bait and target plasmids by sequential transformation.

Gene cloning for Co-IP

To investigate the interaction between the PLS peptide and the ethylene receptor ETR1, two DNA constructs were created by Gateway cloning. The 105-bp *PLS* gene (without the stop codon) was inserted into the pEarlyGate103 (pEG103) destination vector, which contained the *pro35S* promoter and a C-terminal GFP tag, to produce a vector containing *pro35S::PLS:GFP* DNA. The ETR1 cDNA was inserted into the pEarlyGate301 (pEG301) vector to create a *pro35S::ETR1:HA* construct to express an ETR1 protein with a C-terminal HA tag. *pro35S::GFP* was used as a control. The primers used to clone ETR1 and PLS are found in supplemental Table 9.

Infiltration into N. benthamiana

Constructs were transiently expressed in *N. benthamiana* (tobacco) leaves as described previously (Voinnet et al., 2003). Experiments were replicated up to five times. Competent *Agrobacterium tumefaciens* GV3101 cells were transformed with the desired plasmid containing the gene of interest and injected with a syringe. The plants were approximately 7–10 weeks old; the chosen leaves were healthy and 3–6 cm in length, and 3 to 4 leaves were infiltrated with each construct.

Protein extraction and PLS/ETR1 Co-IP

Total protein was extracted from the infiltrated *N. benthamiana* plants 3 days after infiltration for Co-IP experiments to investigate the interaction between PLS and ETR1, essentially as described previously (Srivastava et al., 2020). For competition assays, 5 or 25 nM full-length PLS peptide was also infiltrated in the presence of 50 μM MG-132 (a proteasome inhibitor) 30 min prior to tissue freezing. ChromoTek (Planegg, Germany) anti-GFP beads were used to immunoprecipitate the PLS:GFP protein, and Sigma-Aldrich (St. Louis, MO, USA) anti-HA beads were used for the HA-tagged ETR1.

SDS–PAGE was used to separate protein fragments. The complexed proteins from the pulldown assay were analyzed on 10%–12% acrylamide gels. Membranes were incubated with primary antibody for 2.5 h (GFP, Abcam, Cambridge, UK: rabbit, 1:10 000; HA, Roche, rat, 1:3000; Rubisco large subunit, Agrisera, rabbit, 1:10 000). Excess primary antibody was then removed by washing three times in 2× TBST (150 mM NaCl, 10 mM Tris, 0.1% v/v Tween 20 [pH 7.4]) for 2, 5, and 10 min, and then incubated for 1 h with the ECL peroxidase-labeled anti-rabbit or anti-rat IgG secondary antibody diluted 1:20 000 in TBST. Excess secondary antibody was again removed by washing three times in 1× TBST. To visualize the probed blot, the membrane was incubated with ECL Western Blotting Detection Reagent immediately prior to imaging. The horseradish peroxidase conjugated to the secondary antibody was detected using X-ray film. The experiment was carried out four times with consistent results.

Estimation of synthetic PLS concentration

Freeze-dried synthetic PLS peptide (Cambridge Research Biochemicals) was dissolved in DMSO. An aliquot was added to aqueous buffer (10 mM HEPES [pH 7], 20 mM NaCl, 80 mM KCl), and absorbance was recorded at 280 nm. PLS concentration was estimated from the absorbance and the ProtParam estimated extinction coefficient of 2980 M^{-1} cm^{-1}. Concurrently, a sample was submitted for quantitative amino acid analysis (Abingdon Health Laboratory Services). From this analysis, a conversion factor of 2.27 was generated, which was applied to the concentrations determined by $A_{280\ nm}$.

ATX1 purification

E. coli BL21(DE3) containing pETatx1 was used to overexpress the wild-type *ATX1* gene from *Arabidopsis thaliana* (optimized for expression in *E. coli*, NovoPro Bioscience). Harvested cells were collected and frozen at −20°C overnight, then defrosted and resuspended in 20 mM HEPES (pH 7.0), 10 mM EDTA, 100 mM NaCl, and 10 mM DTT. Cells were sonicated (Bandelin Sonoplus), and the supernatant was separated by size-exclusion chromatography (GE Healthcare, HiLoad 26.600 Superdex 75 pg) using metal-free buffer lacking EDTA. Fractions containing ATX1 were incubated overnight and pooled before transfer to an anaerobic chamber (Belle Technology) via desalting column where the reductant was removed. ATX1 was quantified by a combination of Bradford assay and Ellman's reagent to ensure the fully reduced state of the protein. Samples were also analyzed for metal content by inductively coupled plasma mass spectrometry (ICP–MS) to ensure >95% apo-ATX1.

MBP:PLS/mutant peptide purification

A fusion of PLS to MBP was created using the NEBExpress MBP Fusion and Purification System. Two complementary oligonucleotide primers encoding PLS (optimized for expression in *E. coli*) were annealed and inserted into the pMal-c5x plasmid at the *Xmn*I and *Sal*I insertion sites. The three mutants MBP:PLS(C6S), MBP:PLS(C17S), and MBP:PLS (C6S/C17S) were created by site-directed mutagenesis (QuikChange II, Agilent). *E. coli* NEB Express containing the pMal plasmid with the correct MBP:PLS mutant was used to overexpress each protein. Harvested cells were resuspended in 20 mM Tris–HCl (pH 7.4), 200 mM NaCl, and 1 mM EDTA, then frozen at −20°C overnight. Cells were defrosted in cold H_2O, sonicated, purified by ammonium sulfate precipitation (where MBP-PLS precipitates >60% saturation), separated on an MBP-Trap column (GE Healthcare), and eluted using buffer containing 10 mM maltose. MBP:PLS-containing fractions were pooled and concentrated using a centrifugal concentrator (Corning, Spin-X UF 30 kDa) and buffer exchanged by desalting column into a metal-free buffer of 20 mM HEPES (pH 7.0) and 50 mM NaCl in an anaerobic chamber (Belle Technology). Mutants containing thiols were quantified using Ellman's assay, and MBP:PLS(C6S/C17S), which lacks all thiols, was quantified using the Bradford assay alone. Samples were also analyzed for metal content by ICP–MS to ensure >95% apo-protein.

Anaerobic spectroscopic analysis of Cu(I) complexes

All Cu(I) titration experiments were carried out in an anaerobic chamber (Belle Technology) using metal-free CHELEX-treated, degassed buffers. For titration experiments with Cu(I), aqueous $CuSO_4$ stock was quantified in advance by ICP–MS and diluted to working concentrations. The reductant NH_2OH was included at a final concentration of 1 mM to maintain Cu(I) in its reduced state. Proteins were diluted in buffer to the final concentration specified in each titration in air-tight quartz cuvettes (Helma), and after addition of probe to the concentration specified, titrated with $CuSO_4$. After each addition, solutions were thoroughly mixed and absorbance spectra recorded using a Lambda 35 UV/Vis spectrophotometer (PerkinElmer). Titration isotherm data were fitted using simulated affinity curves with DynaFit (Kuzmič, 2009).

Interaction studies of PLS with copper transporter ETR1 by microscale thermophoresis

Fluorescently labeled ETR1 truncation mutants (Milić et al., 2018) were added to a dilution series of synthetic PLS in 50 mM HEPES, 150 mM NaCl, and 0.015% (w/v) FosCholine 16 (pH 7.6) or 50 mM Tris, 300 mM

NaCl, and 0.015% (w/v) FosCholine 16 (pH 7.6). Dissociation constants were calculated using GraphPad Prism 5.

Determination of dissociation constants for the PLS–ETR1 interaction

Full-length ETR1 and its truncation mutants were purified and labeled as described previously (Milić et al., 2018). Synthetic PLS (94 μM) was diluted serially in 50 mM Tris and 300 mM NaCl (pH 7.6). Fluorescently labeled receptor was added at a final concentration of 50 nM. Thermophoretic behavior was measured in premium capillaries at 50% LED and 50% microscale thermophoresis power. In case of a binding event, data were fitted using GraphPad Prism 5.

FUNDING

The authors acknowledge financial support from the UK Biotechnology and Biological Sciences Research Council (BB/E006531/1, BBS/B/0773X, and BB/J014516/1 to K.L.; BB/V006002/1 and BB/M011186/1 to N.J.R.) and from the Deutsche Forschungsgemeinschaft (German Research Foundation) (267205415 – SFB 1208 project B06 to G.G.).

ACKNOWLEDGMENTS

The authors declare no competing interests. We thank Prof. Steven Cobb (Department of Chemistry, Durham University) for advice on peptide synthesis and Dr. Andrew Foster (Departments of Biosciences and Chemistry, Durham University) for preliminary peptide–Cu interaction analysis. K.L. acknowledges the invaluable early work linking POLARIS and ethylene signaling carried out by Dr. Paul Chilley, who sadly passed away far too soon, and dedicates this paper to his memory.

AUTHOR CONTRIBUTIONS

K.L. and N.J.R. initiated the project. K.L., J.F.T., N.J.R., A.S., and G.G. designed and supervised various aspects of the work. A.J.M., S.M., W.M., B. O.-P., W.S., W.W., C.T., D.R., F.M.H., C.H., and B.U. performed the experiments and prepared the figures. K.L., N.J.R., and G.G. drafted the initial manuscript, and all authors contributed to reviewing and editing the final version.

SUPPLEMENTAL INFORMATION

Supplemental information is available at *Plant Communications Online*.

Received: January 31, 2025
Revised: May 2, 2025
Accepted: June 23, 2025
Published: June 25, 2025

REFERENCES

Abel, S., Nguyen, M.D., Chow, W., and Theologis, A. (1995). *ASC4*, a primary indoleacetic acid-responsive gene encoding 1-aminocyclopropane-1-carboxylate synthase in *Arabidopsis thaliana*. J. Biol. Chem. **270**:19093–19099.

Andrés-Colás, N., Sancenón, V., Rodríguez-Navarro, S., Mayo, S., Thiele, D.J., Ecker, J.R., Puig, S., and Peñarrubia, L. (2006). The Arabidopsis heavy metal P-type ATPase HMA5 interacts with metallochaperones and functions in copper detoxification of roots. Plant J. **45**:225–236.

Bauer, M., and Papenbrock, J. (2002). Identification and characterization of single-domain thiosulfate sulfurtransferases from *Arabidopsis thaliana*. FEBS Lett. **532**:427–431.

Binder, B.M., Rodríguez, F.I., and Bleecker, A.B. (2010). The copper transporter RAN1 is essential for biogenesis of ethylene receptors in Arabidopsis. J. Biol. Chem. **285**:37263–37270.

Bishopp, A., Help, H., El-Showk, S., Weijers, D., Scheres, B., Friml, J., Benková, E., Mähönen, A.P., and Helariutta, Y. (2011). A mutually inhibitory interaction between auxin and cytokinin specifies vascular pattern in roots. Curr. Biol. **21**:917–926.

Brach, T., Soyk, S., Müller, C., Hinz, G., Hell, R., Brandizzi, F., and Meyer, A.J. (2009). Non-invasive topology of membrane proteins in the secretory pathway. Plant J. **57**:534–541.

Burkhead, J.L., Gogolin Reynolds, K.A., Abdel-Ghany, S.E., Cohu, C. M., and Pilon, M. (2009). Copper homeostasis. New Phytol. **182**:799–816.

Campos, M.L., de Souza, C.M., de Oliveira, K.B.S., Dias, S.C., and Franco, O.L. (2018). The role of antimicrobial peptides in plant immunity. J. Exp. Bot. **69**:4997–5011.

Capraro, J., Sessa, F., Magni, C., Scarafoni, A., Maffioli, E., Tedeschi, G., Croy, R.R.D., and Duranti, M. (2015). Proteolytic cleavage at twin arginine residues affects structural and functional transitions of Lupin seed 11S storage globulin. PLoS One **10**:e0117406.

Casareno, R.L., Waggoner, D., and Gitlin, J.D. (1998). The copper chaperone CCS directly interacts with copper/zinc superoxide dismutase. J. Biol. Chem. **273**:23625–23628.

Casson, S.A., Chilley, P.M., Topping, J.F., Evans, I.M., Souter, M.A., and Lindsey, K. (2002). The *POLARIS* gene of Arabidopsis encodes a predicted peptide required for correct root growth and leaf vascular patterning. Plant Cell **14**:1705–1721.

Chang, C. (2003). Ethylene signaling: the MAPK module has finally landed. Trends Plant Sci. **8**:365–368.

Chang, C., Kwok, S.F., Bleecker, A.B., and Meyerowitz, E.M. (1993). Arabidopsis ethylene-response gene *ETR1* - similarity of product to 2-component regulators. Science **262**:539–544.

Chen, G., Li, J., Han, H., Du, R., and Wang, X. (2022). Physiological and molecular mechanisms of plant responses to copper stress. Int. J. Mol. Sci. **23**:12950.

Chen, Y.F., Randlett, M.D., Findell, J.L., and Schaller, G.E. (2002). Localization of the ethylene receptor ETR1 to the endoplasmic reticulum of Arabidopsis. J. Biol. Chem. **277**:19861–19866.

Chilley, P.M., Casson, S.A., Tarkowski, P., Hawkins, N., Wang, K.L.C., Hussey, P.J., Beale, M., Ecker, J.R., Sandberg, G.K., and Lindsey, K. (2006). The POLARIS peptide of Arabidopsis regulates auxin transport and root growth via effects on ethylene signaling. Plant Cell **18**:3058–3072.

Chu, C.C., Lee, W.C., Guo, W.Y., Pan, S.M., Chen, L.J., Li, H.M., and Jinn, T.L. (2005). A copper chaperone for superoxide dismutase that confers three types of copper/zinc superoxide dismutase activity in Arabidopsis. Plant Physiol. **139**:425–436.

Clough, S.J., and Bent, A.F. (1998). Floral dip: a simplified method for *Agrobacterium*-mediated transformation of *Arabidopsis thaliana*. Plant J. **16**:735–743.

Dobin, A., Davis, C.A., Schlesinger, F., Drenkow, J., Zaleski, C., Jha, S., Batut, P., Chaisson, M., and Gingeras, T.R. (2013). STAR: ultrafast universal RNA-seq aligner. Bioinformatics **29**:15–21.

Duckert, P., Brunak, S., and Blom, N. (2004). Prediction of preprotein convertase cleavage sites. Protein Eng. Des. Sel. **17**:107–112.

Gao, Z., Chen, Y.-F., Randlett, M.D., Zhao, X.-C., Findell, J.L., Kieber, J.J., and Schaller, G.E. (2003). Localization of the Raf-like kinase CTR1 to the endoplasmic reticulum of Arabidopsis through participation in ethylene receptor signaling complexes. J. Biol. Chem. **278**:34725–34732.

Grefen, C., Staedele, K., Ruzicka, K., Obrdlik, P., Harter, K., and Horak, J. (2008). Subcellular localization and *in vivo* interactions of the *Arabidopsis thaliana* ethylene receptor family members. Mol. Plant **1**:308–320.

Hall, A.E., Findell, J.L., Schaller, G.E., Sisler, E.C., and Bleecker, A.B. (2000). Ethylene perception by the ERS1 protein in Arabidopsis. Plant Physiol. **123**:1449–1458.

Hirayama, T., Kieber, J.J., Hirayama, N., Kogan, M., Guzman, P., Nourizadeh, S., Alonso, J.M., Dailey, W.P., Dancis, A., and Ecker, J.R. (1999). RESPONSIVE-TO-ANTAGONIST1, a Menkes/Wilson disease-related copper transporter, is required for ethylene signaling in Arabidopsis. Cell **97**:383–393.

Hoppen, C., Müller, L., Hänsch, S., Uzun, B., Milić, D., Meyer, A.J., Weidtkamp-Peters, S., and Groth, G. (2019). Soluble and membrane-bound protein carrier mediate direct copper transport to the ethylene receptor family. Sci. Rep. **9**:10715.

Hua, J., Chang, C., Sun, Q., and Meyerowitz, E.M. (1995). Ethylene insensitivity conferred by Arabidopsis *ERS* gene. Science **269**:1712–1714.

Hua, J., Sakai, H., Nourizadeh, S., Chen, Q.G., Bleecker, A.B., Ecker, J. R., and Meyerowitz, E.M. (1998). EIN4 and ERS2 are members of the putative ethylene receptor gene family in Arabidopsis. Plant Cell **10**:1321–1332.

Johnson, P.R., and Ecker, J.R. (1998). The ethylene gas signal transduction pathway: A molecular perspective. Annu. Rev. Genet. **32**:227–254.

Kim, J.S., Jeon, B.W., and Kim, J. (2021). Signaling peptides regulating abiotic stresses in plants. Front. Plant Sci. **12**:704490.

Kuzmič, P. (2009). DynaFit—a software package for enzymology. Methods Enzymol. **467**:247–280.

Lee, H., Sparkes, I., Gattolin, S., Dzimitrowicz, N., Roberts, L.M., Hawes, C., and Frigerio, L. (2013). An Arabidopsis reticulon and the atlastin homologue *RHD3-like2* act together in shaping the tubular endoplasmic reticulum. New Phytol. **197**:481–489.

Li, W., Lacey, R.F., Ye, Y., Lu, J., Yeh, K.-C., Xiao, Y., Li, L., Wen, C.-K., Binder, B.M., and Zhao, Y. (2017). Triplin, a small molecule, reveals copper ion transport in ethylene signaling from ATX1 to RAN1. PLoS Genet. **13**:e1006703.

Liu, J., Mehdi, S., Topping, J., Tarkowski, P., and Lindsey, K. (2010). Modelling and experimental analysis of hormonal crosstalk in Arabidopsis. Mol. Syst. Biol. **6**:373.

Liu, J., Mehdi, S., Topping, J., Friml, J., and Lindsey, K. (2013). Interaction of PLS and PIN and hormonal crosstalk in Arabidopsis root development. Front. Plant Sci. **4**:75.

Love, M.I., Huber, W., and Anders, S. (2014). Moderated estimation of fold change and dispersion for RNA-seq data with DESeq2. Genome Biol. **15**:550.

Ma, Z., Hu, L., and Jiang, W. (2024). Understanding AP2/ERF transcription factor responses and tolerance to various abiotic stresses in plants: A comprehensive review. Int. J. Mol. Sci. **25**:893.

Matsuzaki, Y., Ogawa-Ohnishi, M., Mori, A., and Matsubayashi, Y. (2010). Secreted peptide signals required for maintenance of root stem cell niche in Arabidopsis. Science **329**:1065–1067.

McDaniel, B.K., and Binder, B.M. (2012). ETHYLENE RECEPTOR 1 (ETR1) is sufficient and has the predominant role in mediating inhibition of ethylene responses by silver in *Arabidopsis thaliana*. J. Biol. Chem. **287**:26094–26103.

McDonald, E.F., Jones, T., Plate, L., Meiler, J., and Gulsevin, A. (2023). Benchmarking AlphaFold2 on peptide structure prediction. Structure **31**:111–119.e2.

Milić, D., Dick, M., Mulnaes, D., Pfleger, C., Kinnen, A., Gohlke, H., and Groth, G. (2018). Recognition motif and mechanism of ripening inhibitory peptides in plant hormone receptor ETR1. Sci. Rep. **8**:1–12.

Moore, S., Jervis, G., Topping, J.F., Chen, C., Liu, J., and Lindsey, K. (2024). A *predictive model for ethylene-mediated auxin and cytokinin patterning* in the *Arabidopsis* root. Plant Comms **5**:100886.

Moore, S., Zhang, X., Mudge, A., Rowe, J.H., Topping, J.F., Liu, J., and Lindsey, K. (2015). Spatiotemporal modelling of hormonal crosstalk explains the level and patterning of hormones and gene expression in *Arabidopsis thaliana* wildtype and mutant roots. New Phytol. **207**:1110–1122.

Morgan, M.T., Bourassa, D., Harankhedkar, S., McCallum, A.M., Zlatic, S.A., Calvo, J.S.,, Meloni, G., Faundez, V., and Fahrni, C.J. (2019). Ratiometric two-photon microscopy reveals attomolar copper buffering in normal and Menkes mutant cells. Proc. Natl. Acad. Sci. USA **116**:12167–12172.

Mudge, A.J., Mehdi, S., Michaels, W., Orosa-Puente, B., Shen, W., Tomlinson, C., Wei, W., Hoppen, C., Uzun, B., Roy, D., et al. (2024). Data from: POLARIS is a copper-binding peptide required for ethylene signalling control in *Arabidopsis*. GEO accession GSE256166: https://www.ncbi.nlm.nih.gov/geo/.

Olsson, V., Joos, L., Zhu, S., Gevaert, K., Butenko, M.A., and De Smet, I. (2019). Look closely, the beautiful may be small: Precursor-derived peptides in plants. Annu. Rev. Plant Biol. **70**:153–186.

Ortega-Martinez, O., Pernas, M., Carol, R.J., and Dolan, L. (2007). Ethylene modulates stem cell division in the *Arabidopsis thaliana* root. Science **317**:507–510.

Puig, S., Mira, H., Dorcey, E., Sancenón, V., Andrés-Colás, N., Garcia-Molina, A., Burkhead, J.L., Gogolin, K.A., Abdel-Ghany, S.E., Thiele, D.J., et al. (2007). Higher plants possess two different types of ATX1-like copper chaperones. Biochem. Biophys. Res. Commun. **354**:385–390.

Qu, X., Hall, B.P., Gao, Z., and Schaller, G.E. (2007). A strong constitutive ethylene-response phenotype conferred on Arabidopsis plants containing null mutations in the ethylene receptors *ETR1* and *ERS1*. BMC Plant Biol. **7**:3.

Robinson, N.J., and Winge, D.R. (2010). Copper metallochaperones. Annu. Rev. Biochem. **79**:537–562.

Rodriguez, F.I., Esch, J.J., Hall, A.E., Binder, B.M., Schaller, G.E., and Bleecker, A.B. (1999). A copper cofactor for the ethylene receptor ETR1 from Arabidopsis. Science **283**:996–998.

Rowe, J.H., Topping, J.F., Liu, J., and Lindsey, K. (2016). Abscisic acid regulates root growth under osmotic stress conditions via an interacting hormonal network with cytokinin, ethylene and auxin. New Phytol. **211**:225–239.

Sabatini, S., Beis, D., Wolkenfelt, H., Murfett, J., Guilfoyle, T., Malamy, J., Benfey, P., Leyser, O., Bechtold, N., Weisbeek, P., et al. (1999). An auxin-dependent distal organiser of pattern and polarity in the Arabidopsis root. Cell **99**:463–472.

Sakai, H., Hua, J., Chen, Q.G., Chang, C., Medrano, L.J., Bleecker, A. B., and Meyerowitz, E.M. (1998). *ETR2* is an *ETR1*-like gene involved in ethylene signaling in *Arabidopsis*. Proc. Natl. Acad. Sci. USA **95**:5812–5817.

Schaller, G.E., Ladd, A.N., Lanahan, M.B., Spanbauer, J.M., and Bleecker, A.B. (1995). The ethylene response mediator ETR1 from Arabidopsis forms a disulfide-linked dimer. J. Biol. Chem. **270**:12526–12530.

Schott-Verdugo, S., Müller, L., Classen, E., Gohlke, H., and Groth, G. (2019). Structural model of the ETR1 ethylene receptor transmembrane sensor domain. Sci. Rep. **9**:8869.

Shin, L.-J., Lo, J.-C., and Yeh, K.-C. (2012). Copper chaperone antioxidant protein1 is essential for copper homeostasis. Plant Physiol. **159**:1099–1110.

Srivastava, M., Srivastava, A.K., Orosa-Puente, B., Campanaro, A., Zhang, C., and Sadanandom, A. (2020). SUMO conjugation to BZR1 enables brassinosteroid signaling to integrate environmental cues to shape plant growth. Curr. Biol. **30**:1410–1423.e3.

Thompson, H.L., Shen, W., Matus, R., Kakkar, M., Jones, C., Dolan, D., Grellscheid, S., Yang, X., Zhang, N., Mozaffari-Jovin, S., et al. (2023). MERISTEM-DEFECTIVE regulates the balance between

stemness and differentiation in the root meristem through RNA splicing control. Development **150**:dev201476.

Tian, T., Liu, Y., Yan, H., You, Q., Yi, X., Du, Z., Xu, W., and Su, Z. (2017). agriGO v2.0: a GO analysis toolkit from the agricultural community, 2017 update. Nucleic Acids Res. **45**:W122–W129.

Yu, C.H., Yang, N., Bothe, J., Tonelli, M., Nokhrin, S., Dolgova, N.V., Braiterman, L., Lutsenko, S., and Dmitriev, O.Y. (2017). The metal chaperone Atox1 regulates the activity of the human copper transporter ATP7B by modulating domain dynamics. J. Biol. Chem. **292**:18169–18177.

Voinnet, O., Rivas, S., Mestre, P., and Baulcombe, D. (2003). An enhanced transient expression system in plants based on suppression of gene silencing by the p19 protein of tomato bushy stunt virus. Plant J. **33**:949–956.

Williams, L.E., and Mills, R.F. (2005). P(1B)-ATPases: an ancient family of transition metal pumps with diverse functions in plants. Trends Plant Sci. **10**:491–502.

Willoughby, A.C., and Nimchuk, Z.L. (2021). WOX going on: CLE peptides in plant development. Curr. Opin. Plant Biol. **63**:102056.

Woeste, K.E., and Kieber, J.J. (2000). A strong loss-of-function mutation in RAN1 results in constitutive activation of the ethylene response pathway as well as a rosette-lethal phenotype. Plant Cell **12**:443–455.

Xiao, Z., Brose, J., Schimo, S., Ackland, S.M., La Fontaine, S., and Wedd, A.G. (2011). Unification of the copper(I) binding affinities of the metallo-chaperones Atx1, Atox1, and related proteins: detection probes and affinity standards. J. Biol. Chem. **286**:11047–11055.

Yamasaki, H., Hayashi, M., Fukazawa, M., Kobayashi, Y., and Shikanai, T. (2009). SQUAMOSA promoter binding protein-like7 is a central regulator for copper homeostasis in *Arabidopsis*. Plant Cell **21**:347–361.

Yang, Y., Hao, C., Du, J., Xu, L., Guo, Z., Li, D., Cai, H., Guo, H., and Li, L. (2022). The carboxy terminal transmembrane domain of SPL7 mediates interaction with RAN1 at the endoplasmic reticulum to regulate ethylene signalling in Arabidopsis. New Phytol. **236**:878–892.

Zhang, H., and Li, L. (2013). SQUAMOSA promoter binding protein-like7 regulated microRNA408 is required for vegetative development in *Arabidopsis*. Plant J. **74**:98–109.

Zhang, H., Zhao, X., Li, J., Cai, H.,, Deng, X.W., and Li, L. (2014). MicroRNA408 is critical for the HY5-SPL7 gene network that mediates the coordinated response to light and copper. Plant Cell **26**:4933–4953.

Zhong, S., Lin, Z., and Grierson, D. (2008). Tomato ethylene receptor-CTR interactions: visualization of NEVER-RIPE interactions with multiple CTRs at the endoplasmic reticulum. J. Exp. Bot. **59**:965–972.

Plant Communications, Volume *6*

Supplemental information

POLARIS is a copper-binding peptide that interacts with ETR1 to negatively regulate ethylene signaling in *Arabidopsis*

Anna J. Mudge, Saher Mehdi, Will Michaels, Beatriz Orosa-Puente, Weiran Shen, Charlie Tomlinson, Wenbin Wei, Claudia Hoppen, Buket Uzun, Dipan Roy, Flora M. Hetherington, Jennifer F. Topping, Ari Sadanandom, Georg Groth, Nigel J. Robinson, and Keith Lindsey

Supplementary Text, Figures and Tables for:

POLARIS is a copper-binding peptide that interacts with ETR1 to negatively regulate ethylene signaling in *Arabidopsis*

Anna J. Mudge[1], Saher Mehdi[1], Will Michaels[1,2], Beatriz Orosa-Puente[1], Weiran Shen[1], Charlie Tomlinson[2], Wenbin Wei[1], Claudia Hoppen[3], Buket Uzun[3], Dipan Roy[1], Flora M. Hetherington[1], Jennifer F. Topping[1], Ari Sadanandom[1], Georg Groth[3], Nigel J. Robinson[1,2], Keith Lindsey[1]

Affiliations

[1]*Department of Biosciences, Durham University, Durham DH1 3LE, UK*

[2]*Department of Chemistry, Durham University, Durham DH1 3LE, UK*

[3]*Institute of Biochemical Plant Physiology, Heinrich Heine University Düsseldorf, D-40204 Düsseldorf, Germany*

Supplementary text

Derivation of equations used to describe the metalation of a protein (P) that forms a 2:1 complex (MP_2) with a metal (M).

Cu(I) occupancy of the cytosolic copper chaperone ATX1 is tuned to track with fluctuations in available cytosolic Cu(I) such that its affinity provides a first approximation of Cu(I) availability within this compartment. Based on the determined Cu(I) stoichiometry of *Arabidopsis* ATX1 (Fig. 5H) and subsequent affinity (Fig. S12), Cu(I) occupancy of a 1:2 Cu(I):PLS_2 complex has been calculated as a function of total cytosolic [PLS].

The following equilibrium describes the formation of a 1:2 complex of metal (M) and protein (P)

$$M + 2P \leftrightharpoons MP_2$$

The equilibrium constant for the association of the reaction is

$$\beta_2 = \frac{[MP_2]}{[M][P]^2} \qquad (1)$$

The fractional occupancy of the protein with the metal M can be defined as:

$$Fractional\ occupancy\ (\%) = 100 \text{ x } \frac{2[MP_2]}{[P]_{tot}} \qquad (2)$$

The following is the mass balance equation for the protein

$$[P]_{tot} = [P] + 2[MP_2] \qquad (3)$$

Equations *(2)* and *(3)* can be combined to give

$$\frac{2[MP_2]}{[P]_{tot}} = \frac{2[MP_2]}{[P]+2[MP_2]} \qquad (4)$$

Substituting *(1)* into *(4)* gives

$$\frac{2[MP_2]}{[P]_{tot}} = \frac{2\beta_2[M][P]^2}{[P]+2\beta_2[M][P]^2} \qquad (5)$$

Simplifying leaves the fractional occupancy in terms of metal affinities (β_2), available metal concentration ([M]), and concentration of *apo*- (uncomplexed) protein ([P]).

$$\frac{2[MP_2]}{[P]_{tot}} = \frac{2\beta_2[M]}{[P]^{-1}+2\beta_2[M]} \qquad (6)$$

The *apo*-protein concentration is itself a function of metal affinities, total protein concentration and available metal concentration. Substituting *(1)* into *(3)* and rearranging gives

$$[P]_{tot} = [P] + 2\beta_2[M][P]^2$$

$$So: 2\beta_2[M][P]^2 + [P] - [P]_{tot} = 0 \qquad (7)$$

Can solve this equation for [P] using the quadratic formula:

$$[P] = \frac{-b \pm \sqrt{b^2 - 4ac}}{2a} \qquad (8)$$

Where: $a = 2\beta_2[M],\ b = 1\ and\ c = -[P]_{tot}$

The fractional occupancy of the protein with metal can therefore be expressed as a function of metal affinity, metal availability and total protein concentration (*ie* measurable parameters) by combining equations (*6*) and (*8*)

$$\frac{2[MP_2]}{[P]_{tot}} = \frac{2\beta_2[M]}{\frac{-b \pm \sqrt{b^2 - 4ac}}{2a}^{-1} + 2\beta_2[M]} \qquad (9)$$

Where: $a = 2\beta_2[M],\ b = 1\ and\ c = -[P]_{tot}$

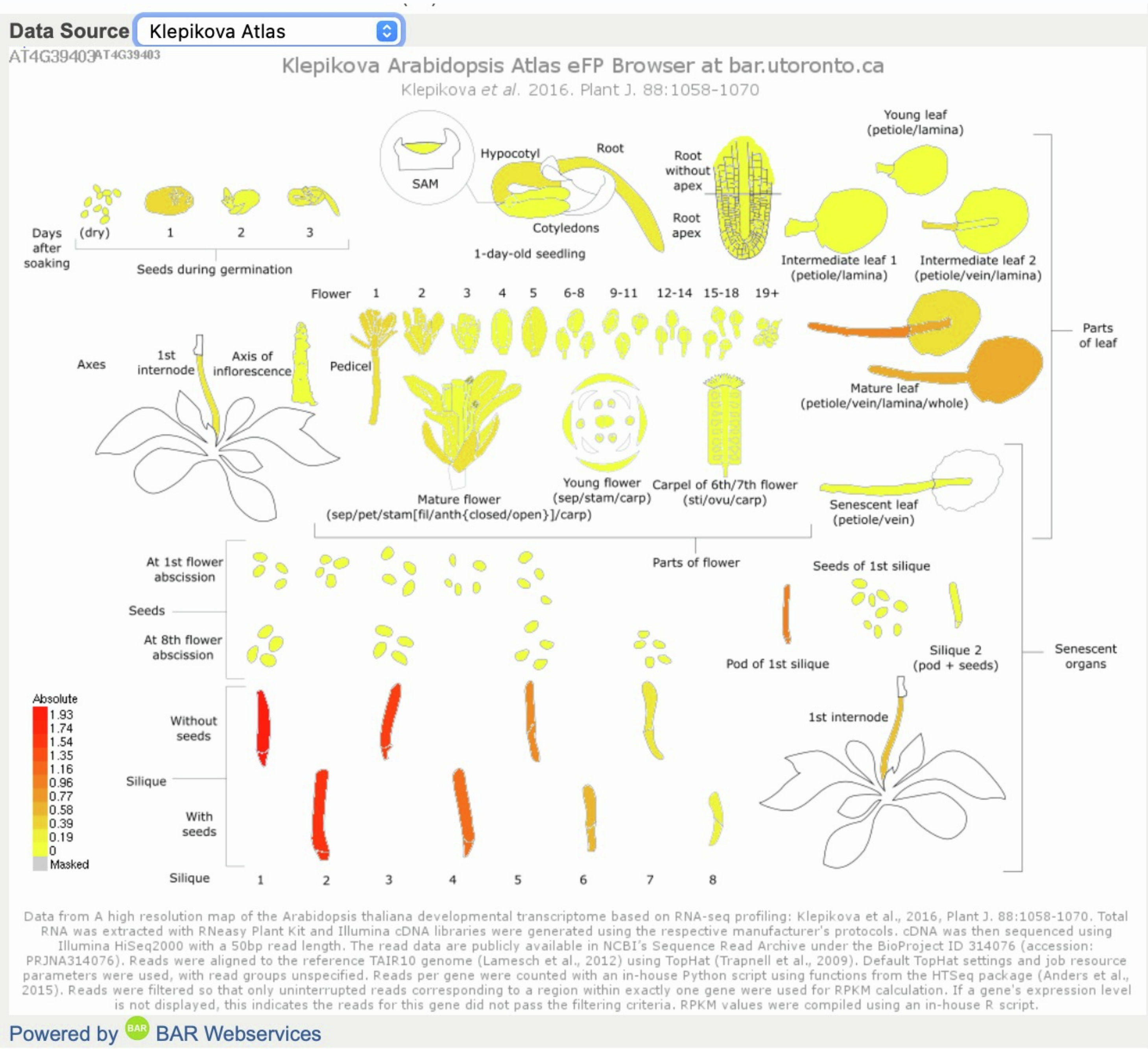

Figure S1. *POLARIS* (AT4G39403) expression.
A high resolution map of the *Arabidopsis thaliana* developmental transcriptome based on RNA-seq profiling: Klepikova et al., 2016, Plant J. 88:1058-1070.

From https://www.arabidopsis.org/servlets/TairObject?id=1001029664&type=locus

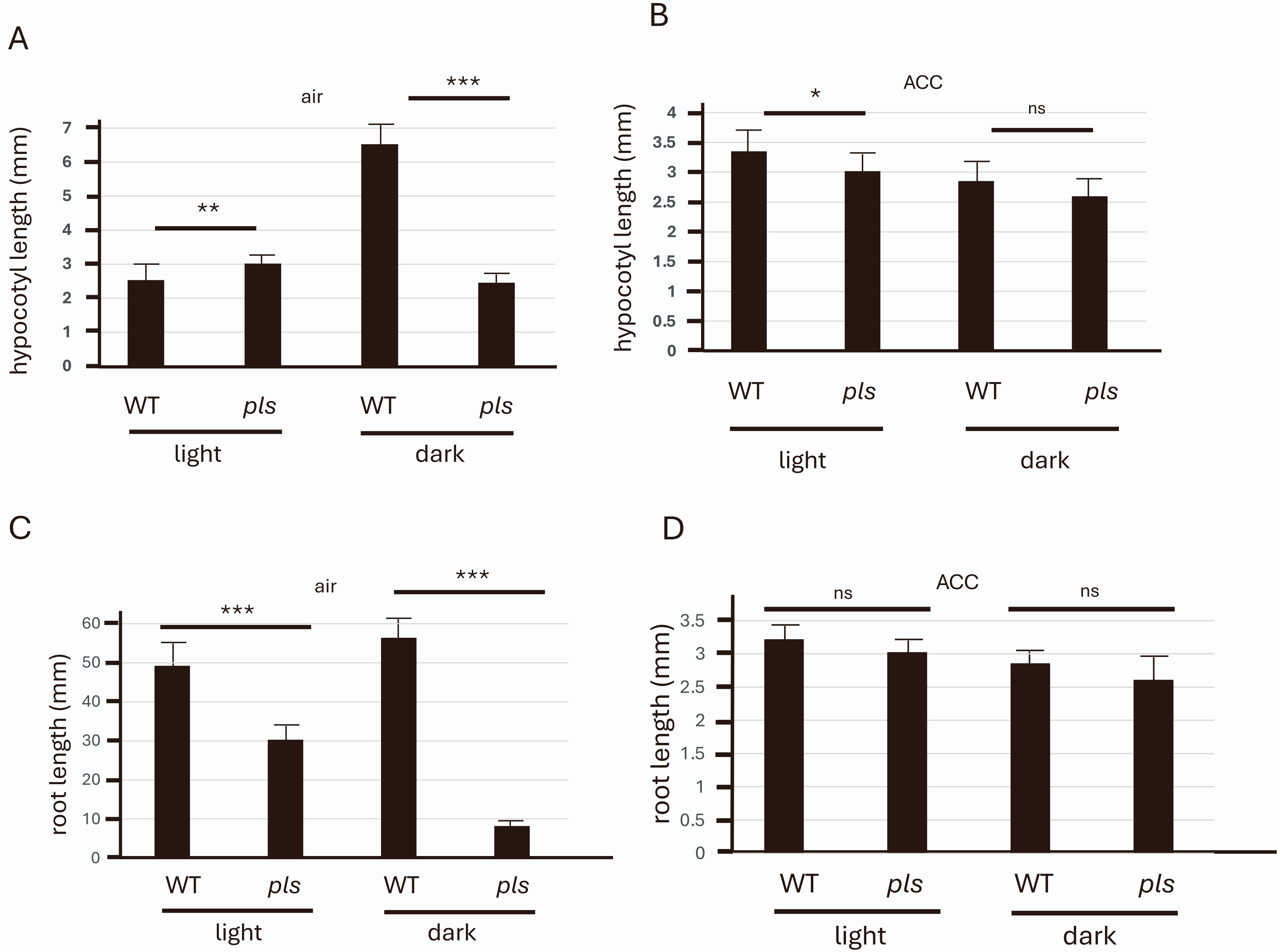
A
air
hypocotyl length (mm)
0
1
2
3
4
5
6
7
**
***
WT
pls
WT
pls
light
dark
B
ACC
hypocotyl length (mm)
0
0.5
1
1.5
2
2.5
3
3.5
4
*
ns
WT
pls
WT
pls
light
dark
C
air
root length (mm)
0
10
20
30
40
50
60
***
***
WT
pls
WT
pls
light
dark
D
ACC
root length (mm)
0
0.5
1
1.5
2
2.5
3
3.5
ns
ns
WT
pls
WT
pls
light
dark

Figure S2. Hypocotyl and root length measurements of 10 d old wild type and *pls* mutant seedlings grown in light or dark, in air or in the presence of 10 μ M ACC. Results represent means ± SD, n = 12. Significant differences determined by Student's t test: ns - no significant difference, * = P value <0.05, ** - P value < 0.01, *** = P value < 0.005.

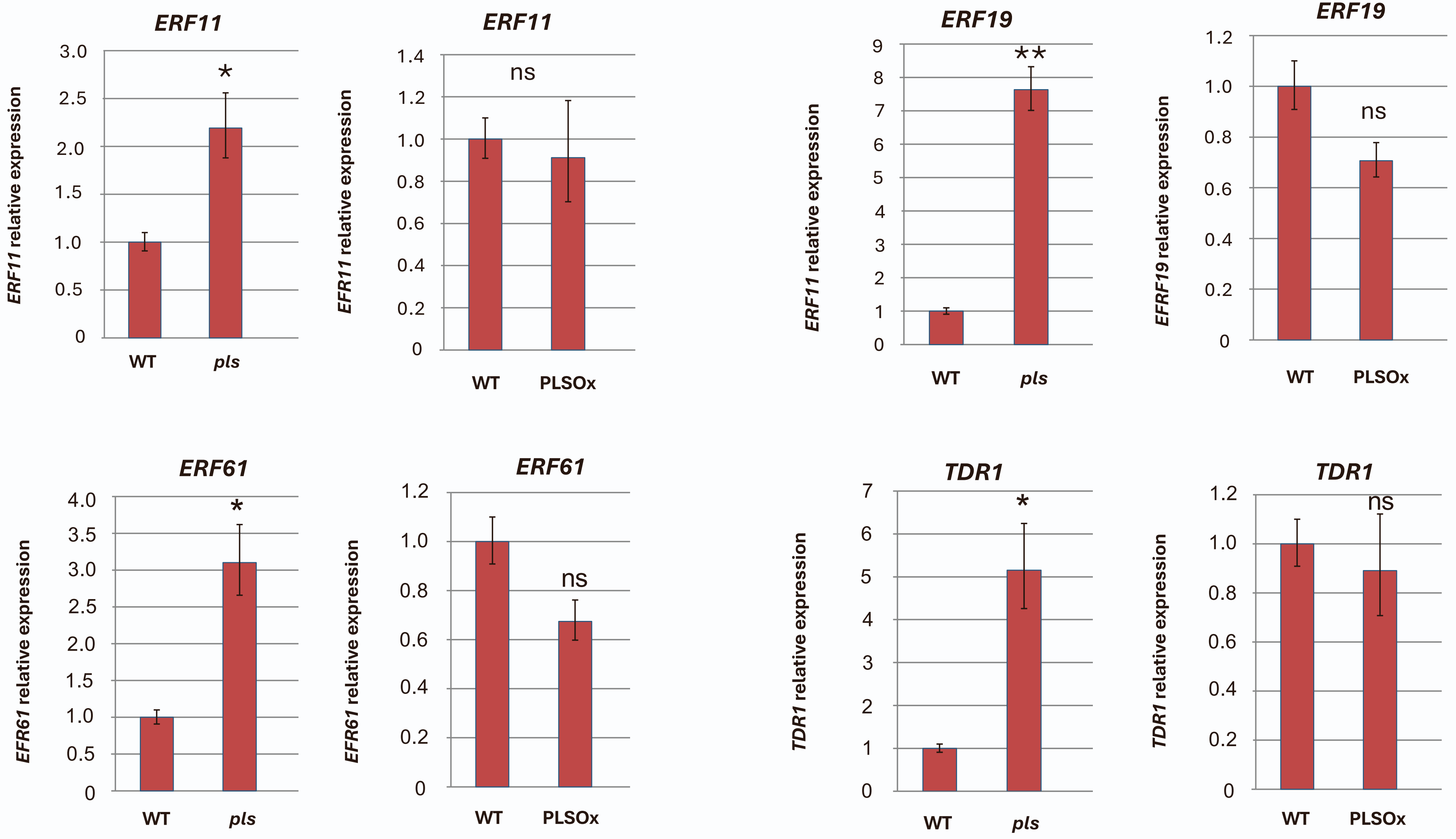

Figure S3. Validation RT-qPCR analysis of genes found to be up-regulated in the *pls* mutant by RNA-seq (Table S1). Values represents means and error bars are SEM (n = 3 biological repeats with three technical repeats) for both 7 day-old total seedlings of *pls* mutants and transgenic PLS overexpressers (PLSOx). *ACTIN1* was used as the reference gene and mutant data are compared to wild type which is normalised as 1. Statistical significance was determined using Student's t-test for independent samples compared to wild type values, with P-values <0.05 (*), P <0.01 (**).

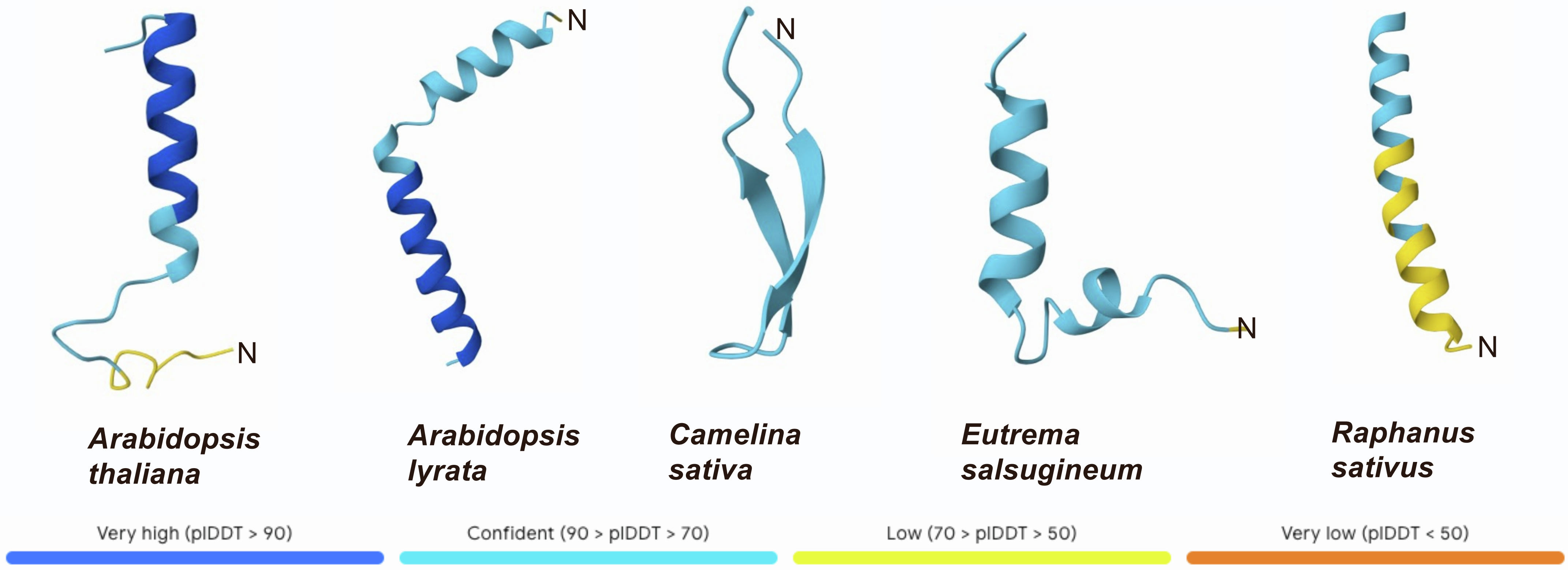

Figure S4. Predicted structures of POLARIS-like peptides from Arabidopsis and four relatives, generated using AlphaFold2. The 36 amino acid POLARIS sequence was used as a BLAST search term. The results show significant alignments in these species but limited structural conservation.

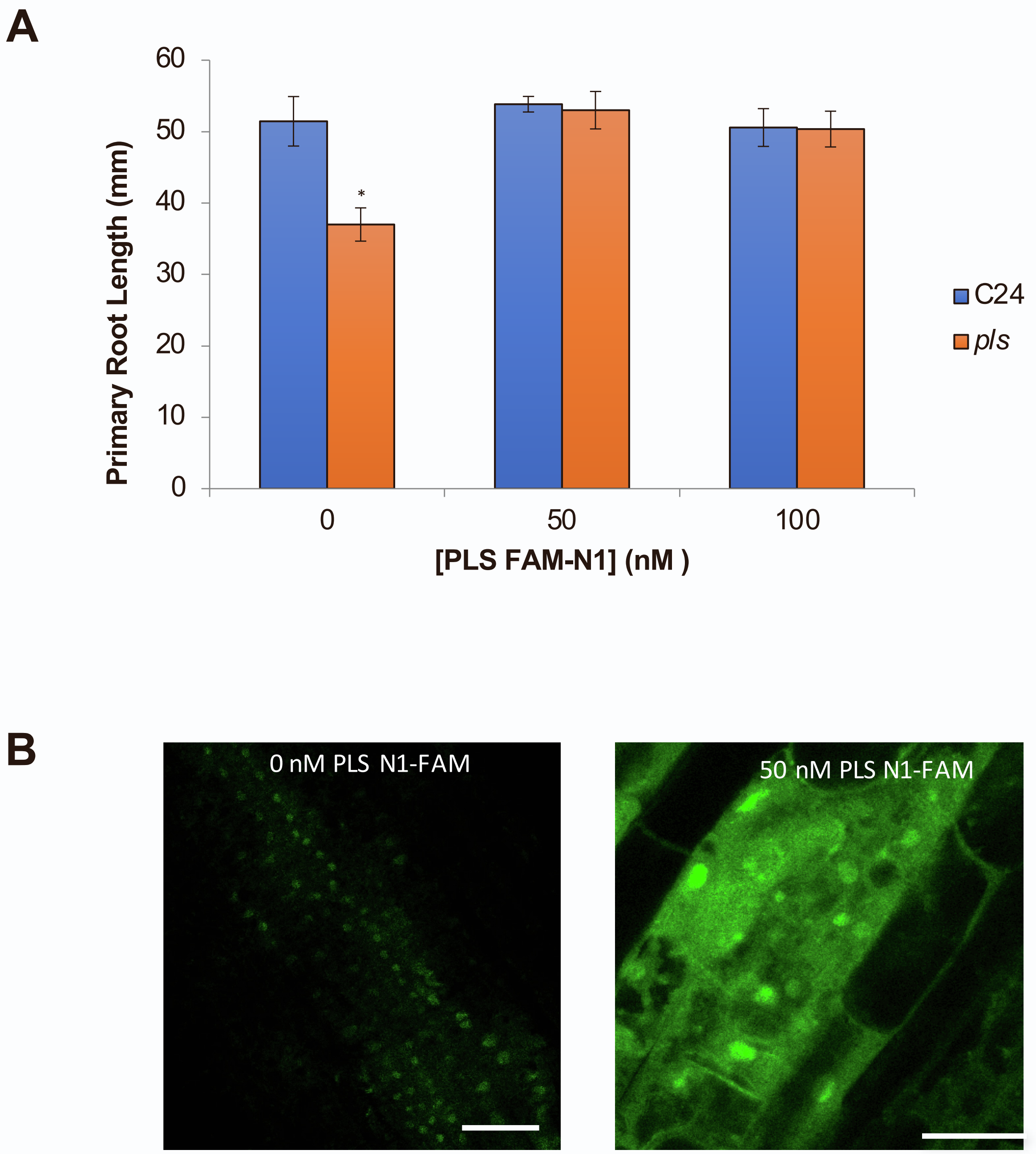

Figure S5. Synthetic PLS peptide is taken up and rescues root development in *pls* seedlings.

(**A**) Effect of fluorescently tagged (5-FAM) synthetic PLS (N1) peptide on primary root length of *Arabidopsis*. Wildtype (C24, blue bars) and *pls* mutant (red bars) seedlings were grown hydroponically in the presence of 0, 50 or 100 nM peptide for 10 days. *: $P < 0.05$, *t*-test between C24 and *pls*. Bars show ± standard errors of the mean, n = 15.

(**B**) 5-FAM-PLS(N1) is taken up by *Arabidopsis* roots. Confocal images of wildtype *Arabidopsis* roots (elongation zone) grown hydroponically for 10 days in the absence of peptide (left panel) and in the presence of 50 nM peptide (right panel). Images are representative of 8 independent root tips imaged. Scale bars = 10 µm.

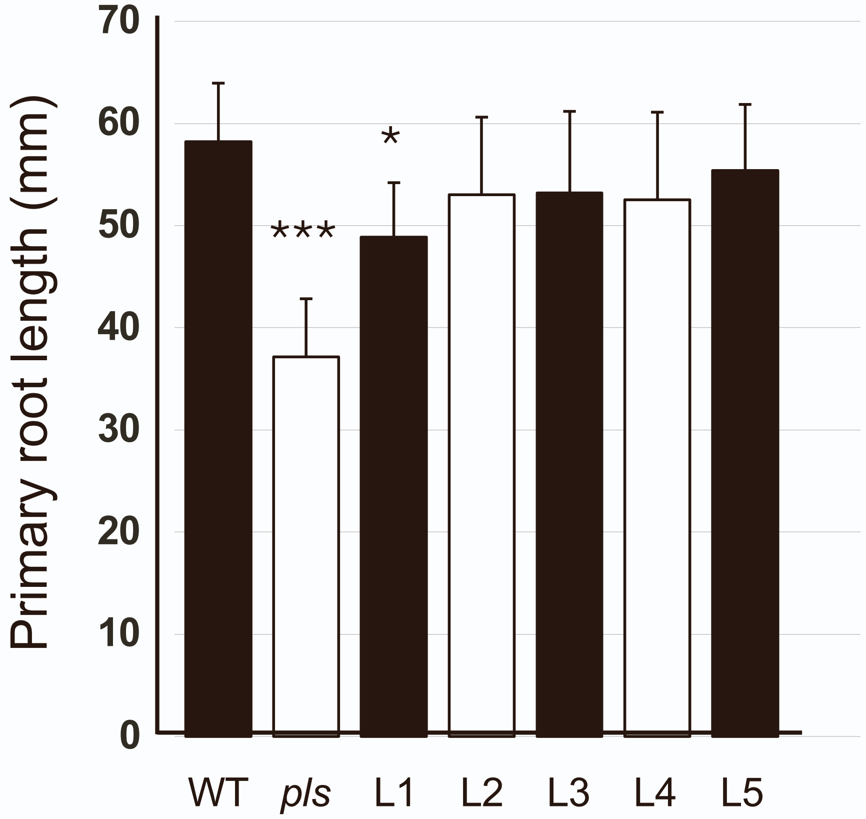

Figure S6. The *proPLS::PLS:GFP* gene fusion complements the *pls* mutant.

Primary root length of seedlings of wild type (WT), *pls* mutant (*pls*), and five independent transgenic lines (L1-L5) expressing *pro::PLS:PLS:GFP* in the *pls* mutant background. Seedlings were grown for 10 days on solid half MS10 agar medium. ***: P <0.0001, * : P <0.01 *t*-test between WT (C24) and *pls* or transgenic line. Bars represent +1 SD, n = 13. No significant difference detected between WT and Lines 2-5.

A

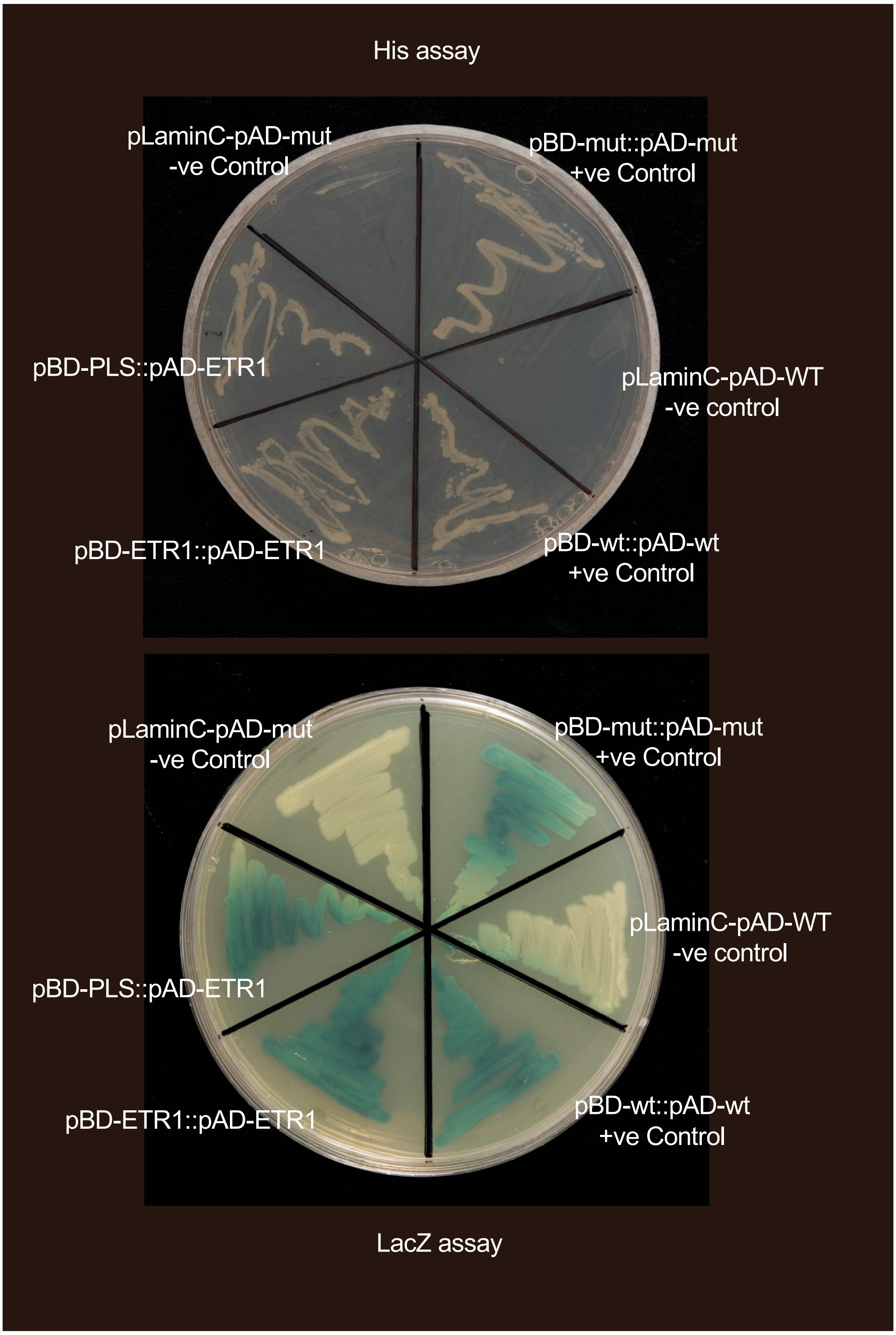
His assay
pLaminC-pAD-mut
-ve Control
pBD-mut::pAD-mut
+ve Control
pBD-PLS::pAD-ETR1
pLaminC-pAD-WT
-ve control
pBD-ETR1::pAD-ETR1
pBD-wt::pAD-wt
+ve Control
pLaminC-pAD-mut
-ve Control
pBD-mut::pAD-mut
+ve Control
pLaminC-pAD-WT
-ve control
pBD-PLS::pAD-ETR1
pBD-ETR1::pAD-ETR1
pBD-wt::pAD-wt
+ve Control
LacZ assay

B

The GAL4 two-hybrid vector system contains six control plasmids listed below:

Control plasmid	Insert description	Vector	Genotype	Function
pGAL4	Wild-type, full length GAL4	pRS415	LEU2, Amp^r	Positive control
pBD-WT	Wild-type fragment C of lamda cl repressor (aa 132-236)	pAD-GAL4 Cam	TRP1, Cam^r	Interaction control
pAD-WT	Wild-type fragment C of lamda cl repressor (aa 132-236)	pBD-GAL4 2.1	LEU2, Amp^r	Interaction control
pBD-MUT	E233K mutant fragment of lamda cl repressor (aa 132-236)	pBD-GAL4 Cam	TRP1, Cam^r	Interaction control
pAD-MUT	E233k mutant fragment of lamda cl repressor (aa 132-236)	pAD-GAL4 2.1	LEU2, Amp^r	Interaction control
pLaminC	Human Lamin C (aa 67-230)	pBD-GAL4	TRP1, Amp^r	Negative control

Figure S7. PLS interacts with ETR1 in yeast.
(A) Yeast 2-hybrid assays showing and ETR1 interacts with PLS and with ETR1, in both His3 (upper plate) and LacZ (lower plate) reporter assays. Positive controls (pBD-wt::pAD-wt and pBD-mut::pAD-mut) and negative controls (pLaminC::pAD-mut and pLaminC::PAD-wt; pBD-PLS::pAD-ETR1 and pBD-ETR1::pAD-ETR1) are shown for each. The control plasmids are described in the Table (B).

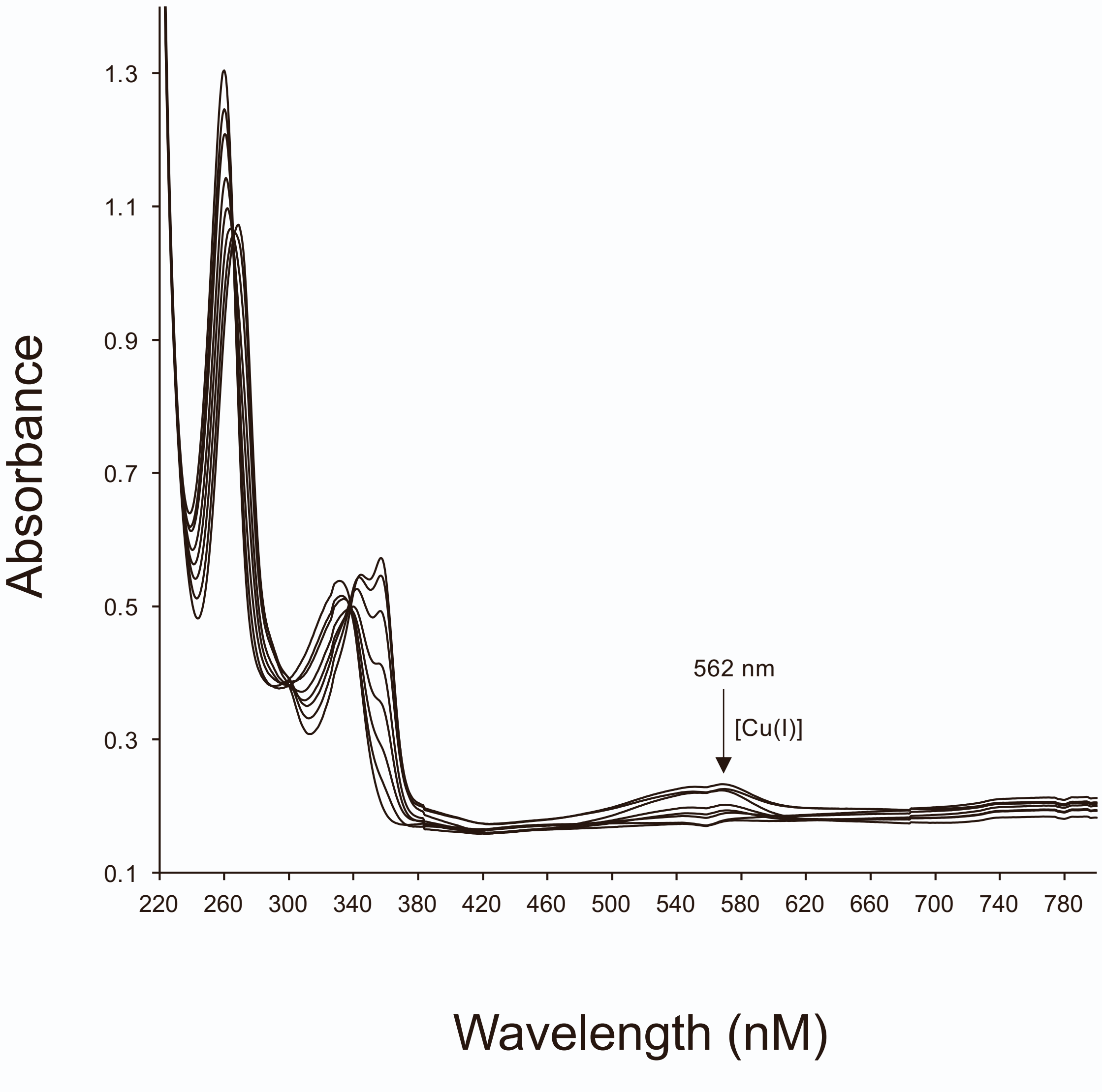

Figure S8. Full data representation from UV-vis spectra shown in Fig. 5D.

Titration of BCA with Cu(I) as in Figure 5D, showing additional copper-dependent features at 562 nm.

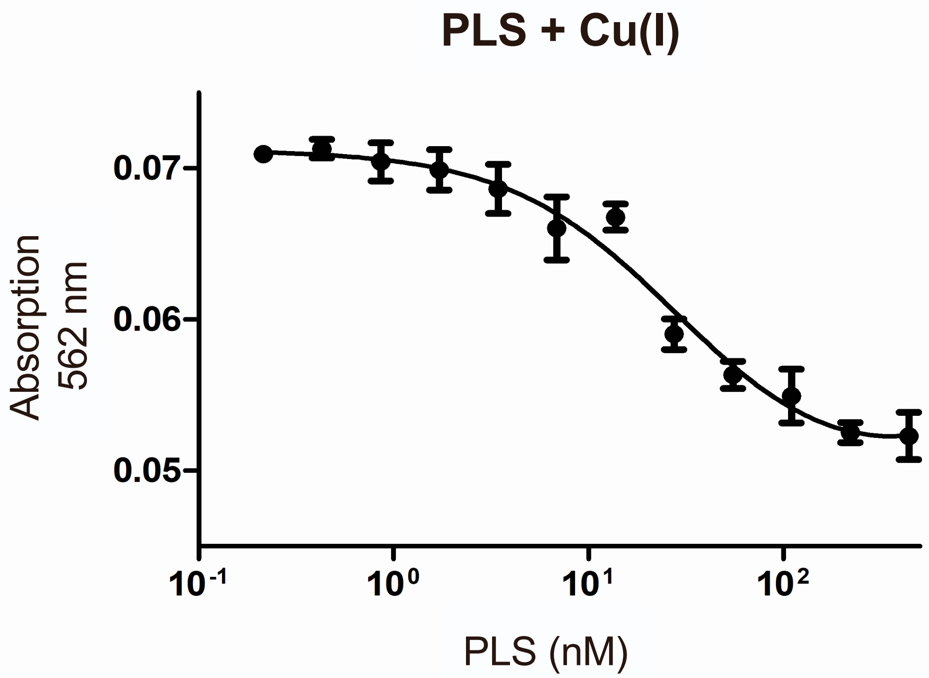

Figure S9. Titration of PLS in the presence of BCA_2-Cu(I)-Complex.
A solution of synthetic PLS in 50 µl buffer (50 mM HEPES, 150 mM NaCl, pH 7.6) was serially diluted from 496 µM to 214 nM. After the addition of 50 µl BCA_2-Cu(I) buffer (50 mM HEPES, 150 mM NaCl, 20 mM ascorbate, 125 µM BCA, 50 µM CuCl, pH 7.6) the absorbance at 562 nm was measured. Discoloration of the pink BCA_2-Cu(I) complex visible in the decrease of absorption at higher peptide concentration indicates that PLS is able to effectively compete and remove copper from the high affinity copper chelator BCA. Bars represent means ± SD, n = 3.

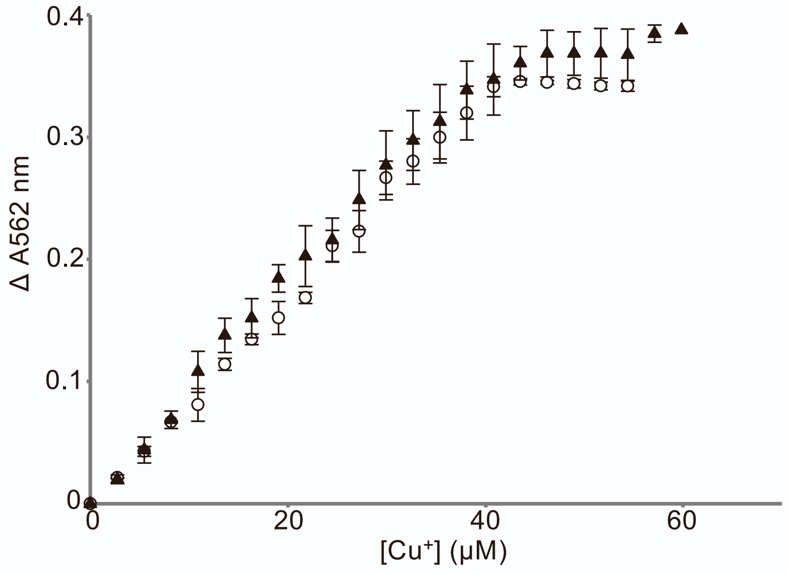

Figure S10. PLS requires C6S and C17S cysteine residues for copper binding.

BCA absorbance in the presence of synthetic PLS FL C6S, C17S ([BCA] = 93.3 μM, [PLS FL C6S, C17S] = 40 μM, closed triangles) or an equivalent volume (to PLS FL C6S, C17S) of DMSO ([BCA] = 87.4, open circles). Values represent means ± SD, n = 3.

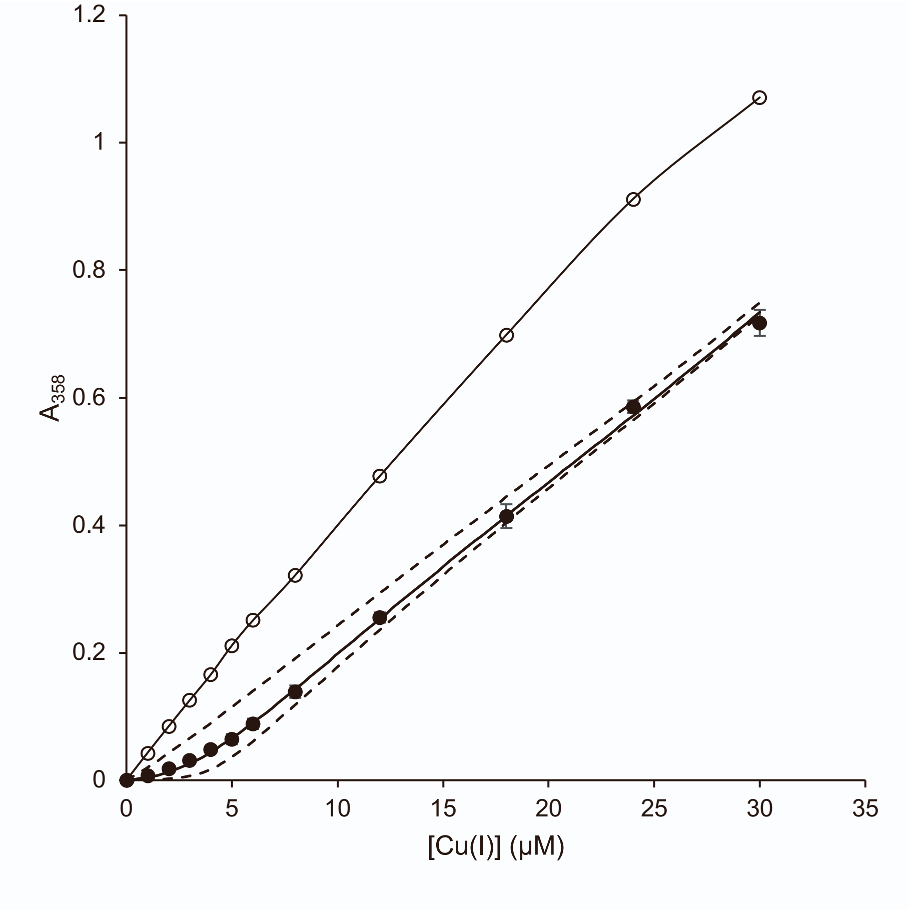

Figure S11. Complete data set for Figure 5F.

$A_{358\ nm}$ binding isotherm of BCA (50 µM) in the presence and absence of 10 µM MBP-PLS (filled and empty circles respectively) upon titration with $CuSO_4$ (recorded anaerobically at pH 7.0) in the presence of 1 mM NH_2OH. Dynafit model (solid line) describes Cu(I)-binding of MBP-PLS when modelled as forming a 2:1 complex with a β_2 value of 3.79 (±1.5) x10^{19} M^{-2} and one additional weak binding event. Dotted lines above and below represent the same model when the affinity is simulated 10x weaker or tighter, respectively (n = 3, ± SD).

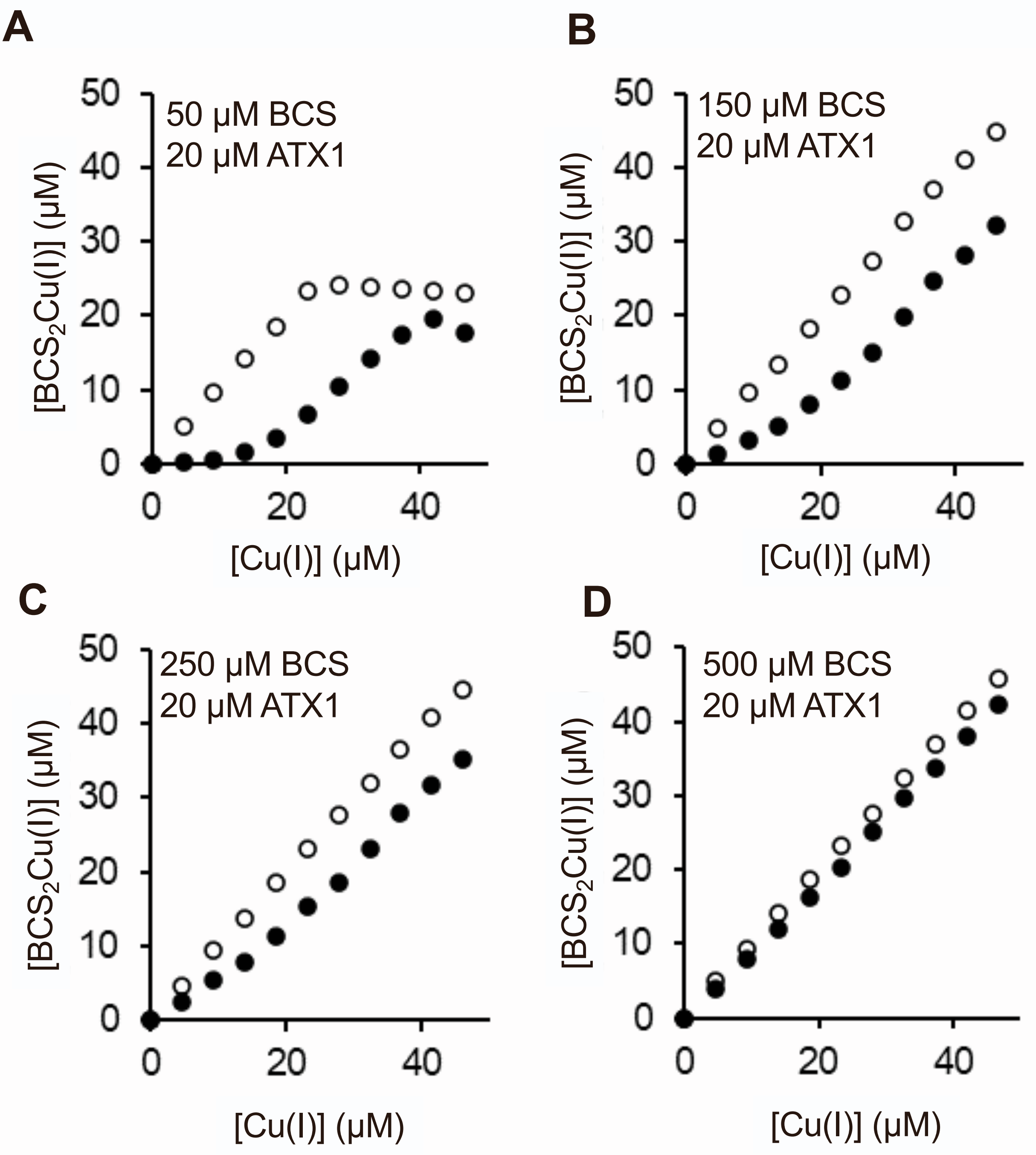

Figure S12. Determination of the Cu(I) binding affinity of the *Arabidopsis* ATX1.

Binding isotherms at A_{483nm} when BCS (50 µM **(A**), 15 µM **(B)**, 250 µM **(C)** or 500 µM **(D)**) in the presence and absence of 20 µM ATX1 (filled and empty circles, respectively), is titrated with Cu(I) under anaerobic conditions. Using the observed Cu(I):ATX1 stoichiometry of 1:1 (Fig. 5H), affinities of 5.79 $x10^{-18}$, 4.83 $x10^{-18}$, 5.10 $x10^{-18}$, 6.14 $x10^{-18}$ respectively, with a mean of 5.47 (±0.6) $x10^{-18}$ M, were calculated.

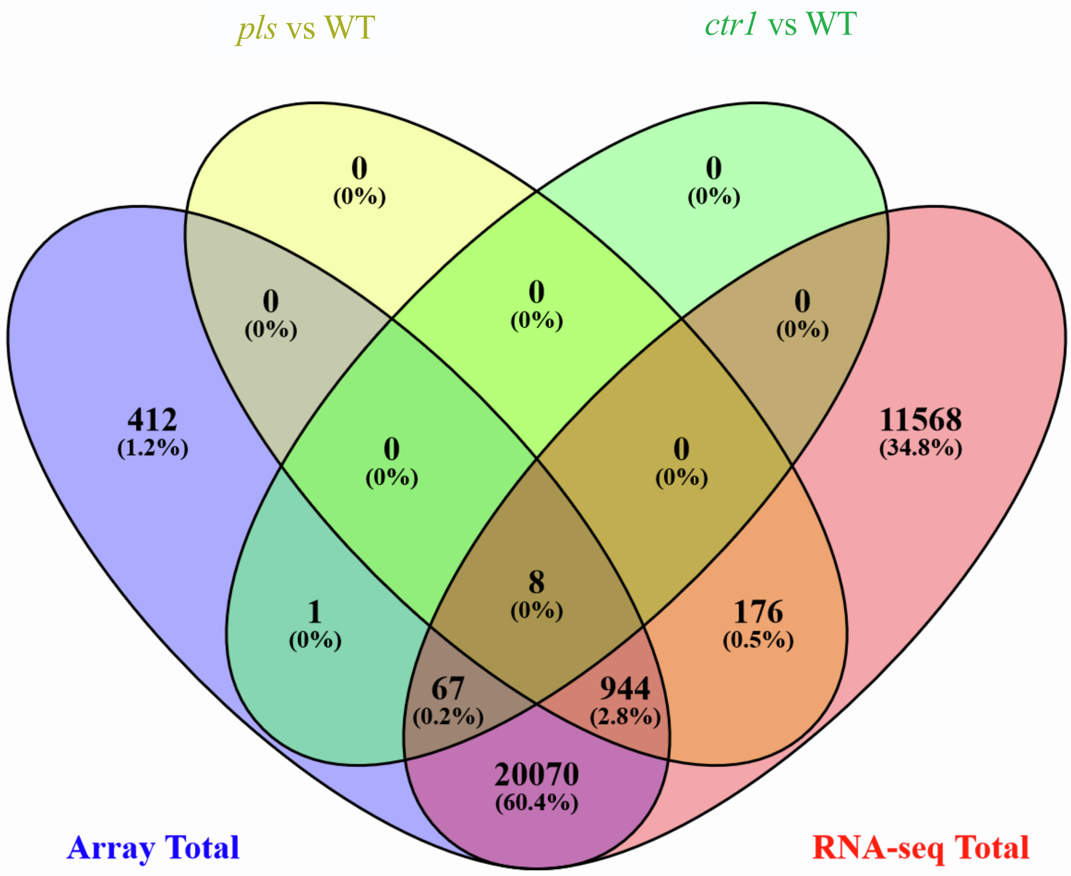

Figure S13. Overlap of DEGs (compared to respective wild types) between the *ctr1* and *pls* mutants.

Venn diagram generated from DEG lists generated by Brodersen *et al*. (2006) *Plant J*. **47**, 532-546, Table S2 for *ctr1 vs.* wild type, and data from Table S1 (this paper) for *pls vs.* wild type. Overlap between the CTR1- and PLS-regulated genes (i.e upregulated in both *ctr1* and *pls* mutants compared to wild type) is significant. P value of Fisher's exact test (alternative='greater') = 0.01959. 21089 genes are analysed in both experiments, of which the proportion of CTR1-regulated genes is 8+67/21089=0.36%. The proportion of CTR1-regulated genes in PLS-regulated genes is increased to 8/(8+944)=0.84%.

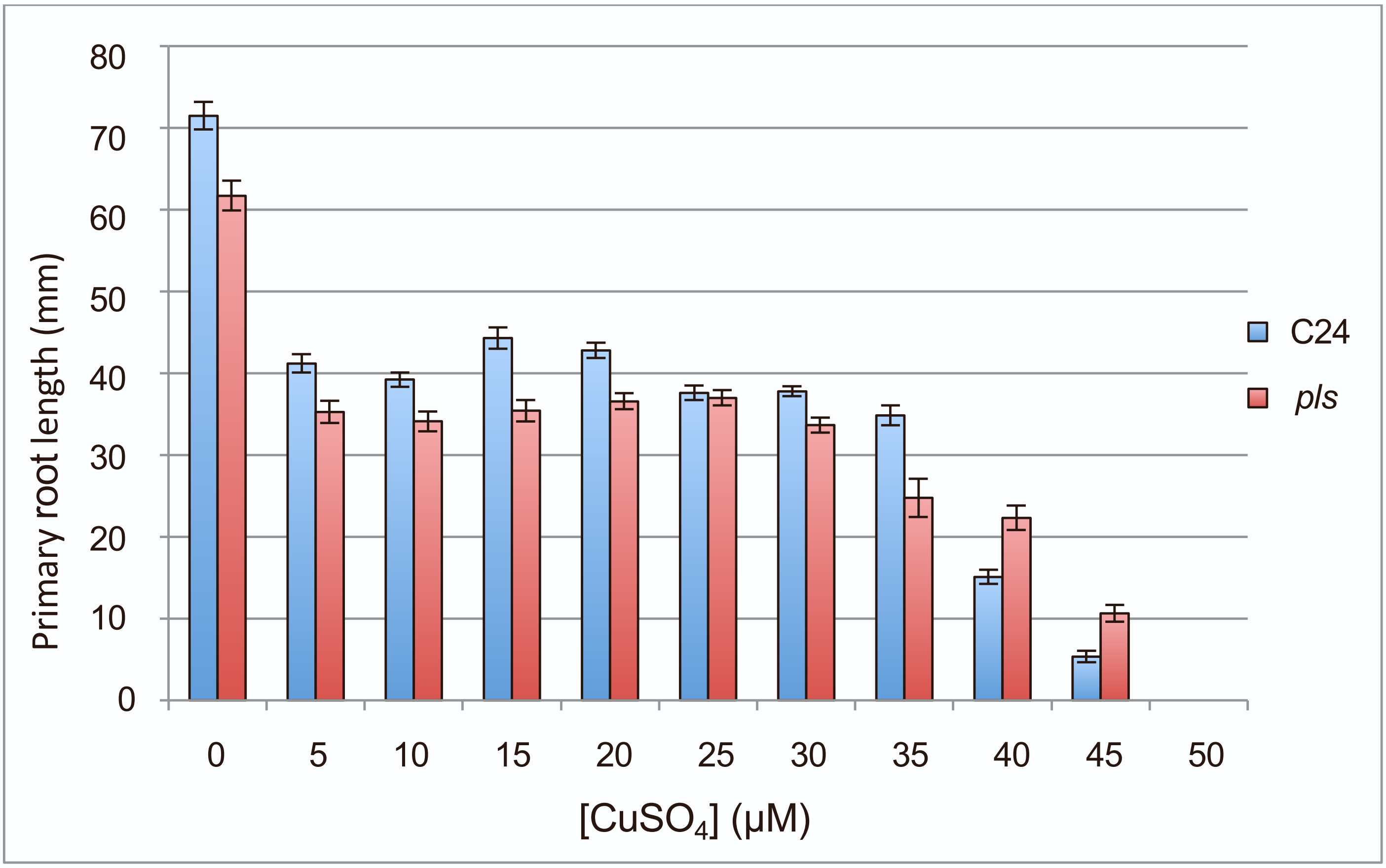

Figure S14. The *pls* mutant primary root is not hypersensitive to exogenous copper.
Seedlings were grown for 12 days in liquid 1⁄2 MS10 medium supplemented with different concentrations of $CuSO_4$. Root length (n = 18 from samples grown on 3 independent plates) was measured after 12 days using ImageJ. Error bars show means ± 1 standard error. Red bars represent *pls* mutant, blue bars represent wild type.

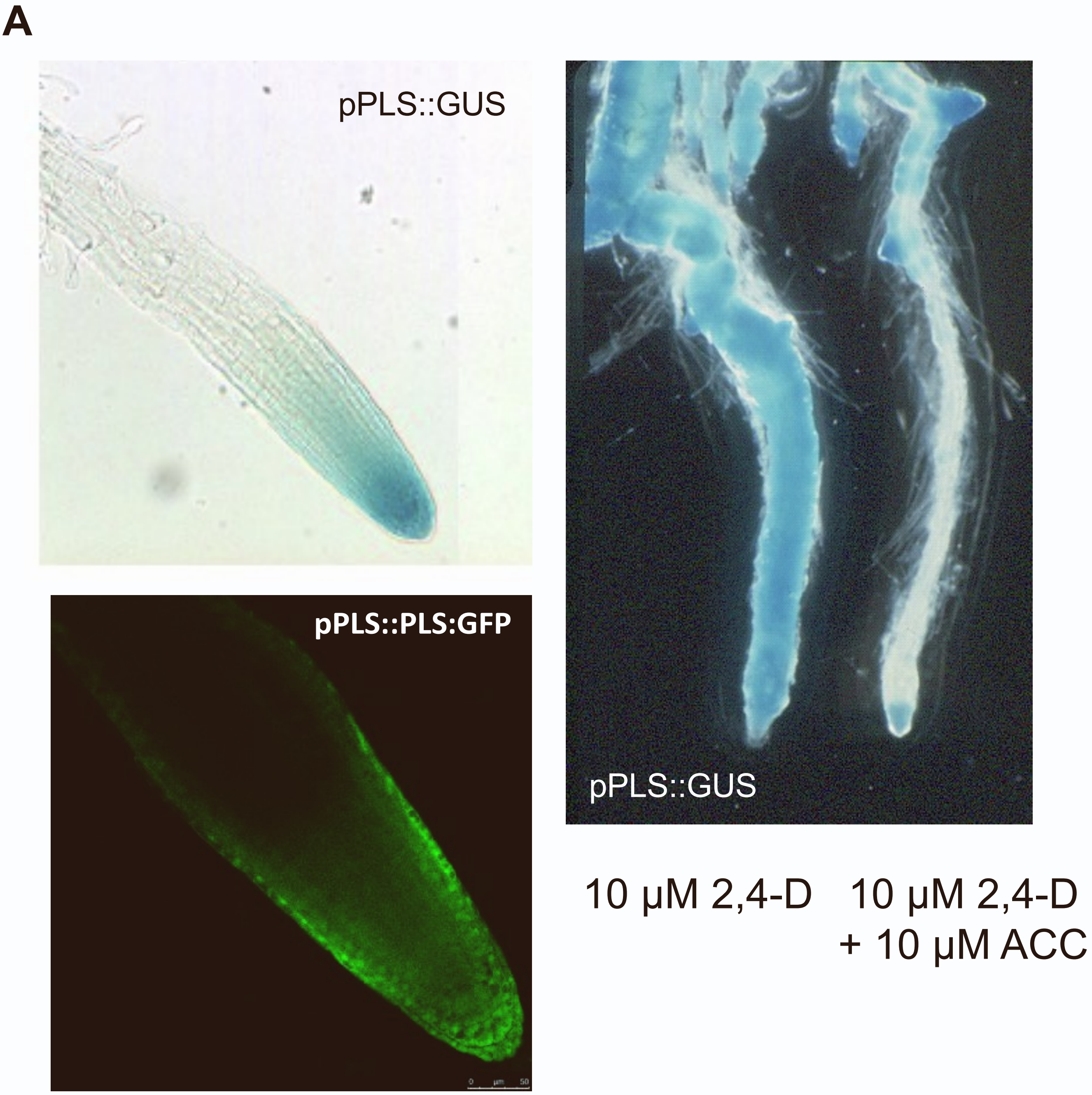

Figure S15. The link between auxin and ethylene signalling effects on *PLS* transcription, and downstream effects on ethylene responses.

(A) Antagonism between auxin and ethylene on PLS expression.
Auxin positively regulates *PLS* gene transcription, while ACC (ethylene precursor) treatment antagonizes the inductive effects of auxin. Left upper panel: pPLS::GUS expression in the Arabidopsis root tip under standard growth conditions. Left lower panel: pPLS::PLS:GFP expression in the Arabidopsis root tip under standard growth conditions. Right panel: pPLS::GUS expression in root tips treated with 10 µM 2,4-D, showing ectopic expression throughout the root (left); and pPLS::GUS expression in root tips treated with 10 µM 2,4-D plus 10 µm ACC, showing the antagonism of the inductive effect of 2,4-D on PLS::GUS expression. Images are representative of 10 independently grown roots.

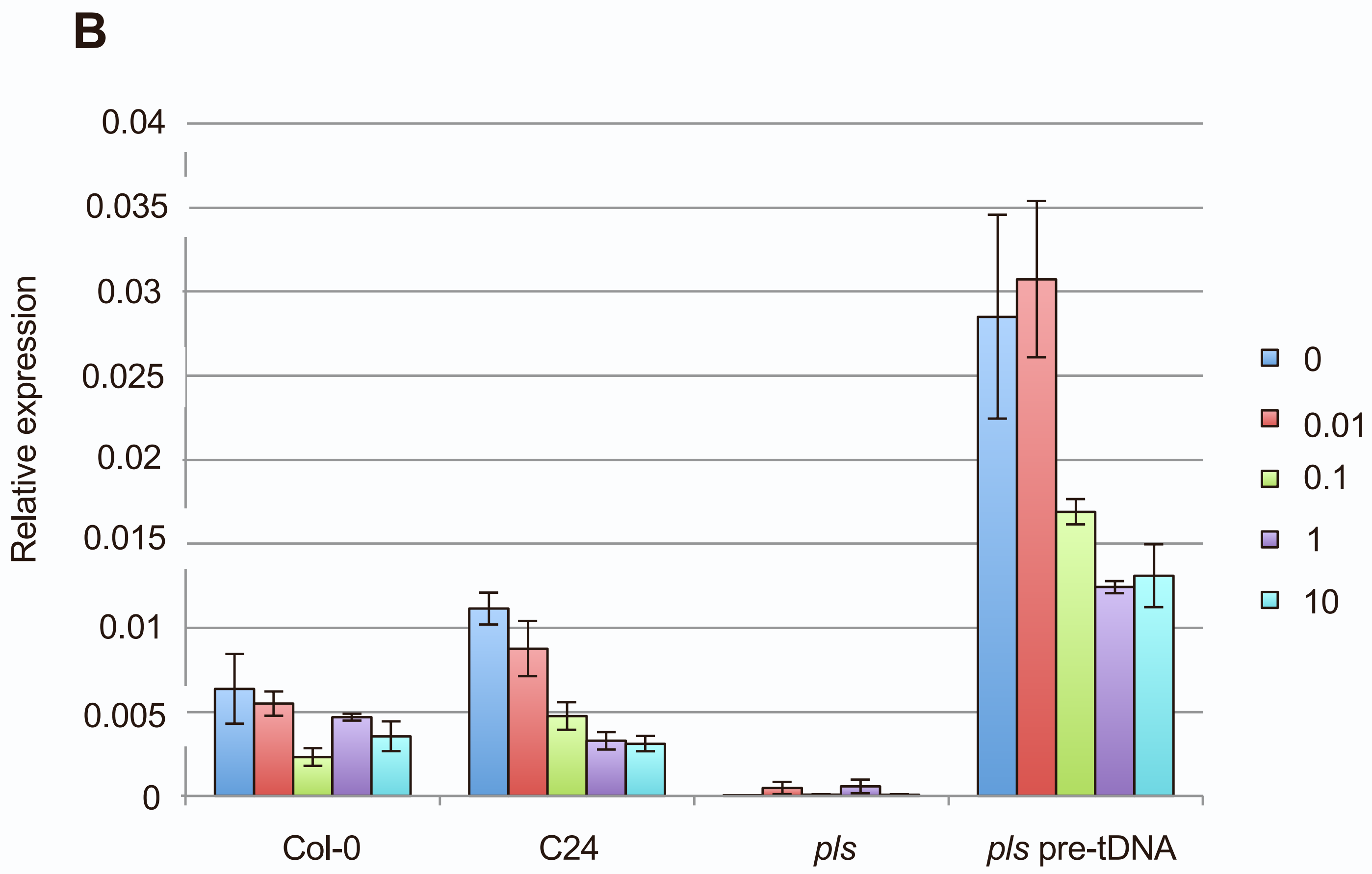

Figure S15. The link between auxin and ethylene signalling effects on *PLS* transcription, and downstream effects on ethylene responses.

(B) *PLS* gene expression under ACC treatment.
Col-0, C24 and *pls* seedlings were treated with 0, 0.01, 0.1, 1 or 10 μM ACC for seven d.a.g and quantitative PCR was carried out on the resulting cDNA. *PLS* transcript levels were measured alongside a reference gene transcript *PP2C.* Comparison is made between the *PLS* expression in Col-0 and C24 wild types and the *pls* mutant, in which the reverse qPCR primer is located beyond the tDNA insertion in the *pls* mutant. In the fourth set of bars, a second set of *PLS* primers were used, located before the tDNA insertion, to detect a truncated and inactive *PLS* transcript. Error bars show means ±1 standard error, n = 3 biological replicates with three technical replicates.

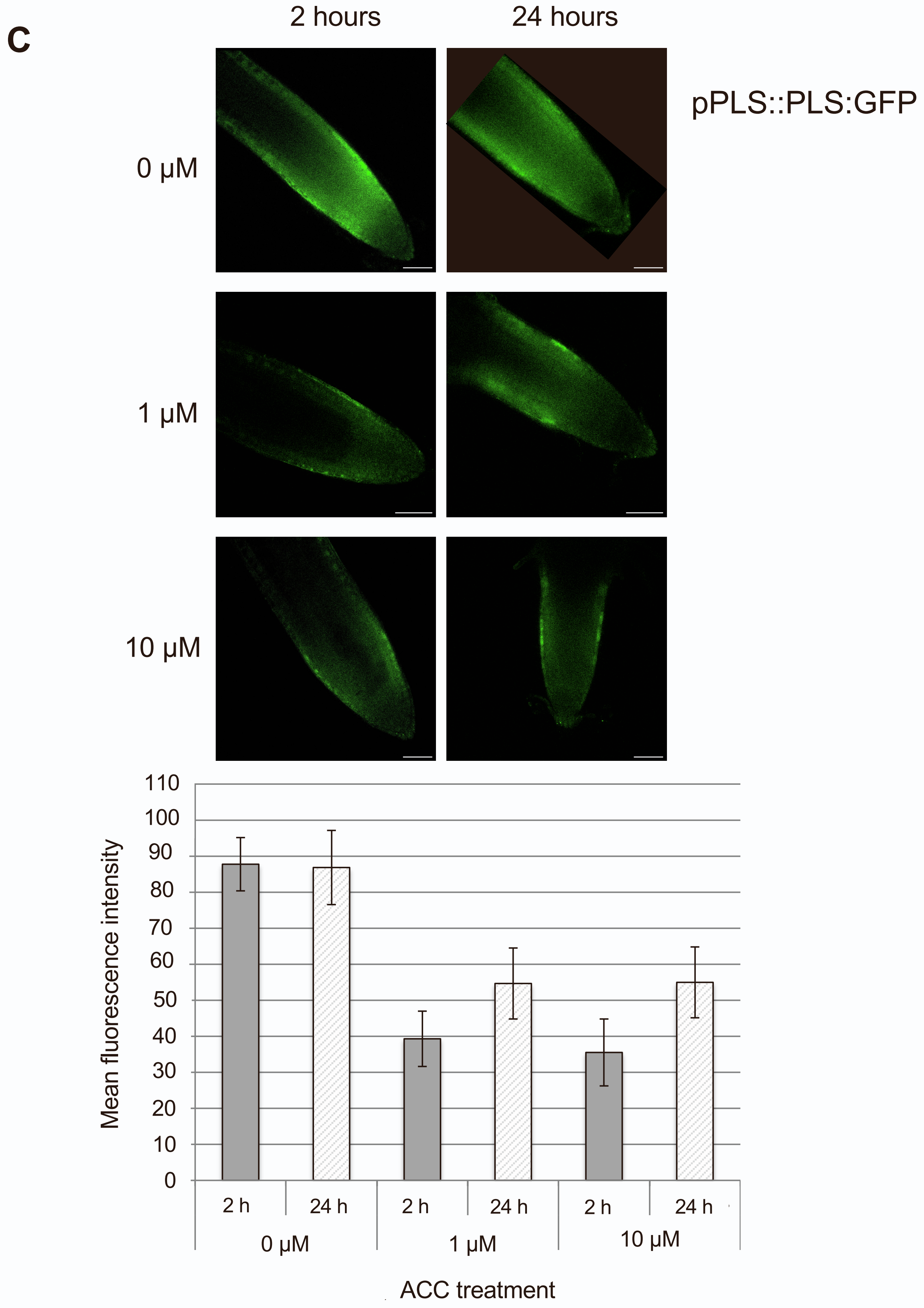

C
2 hours
24 hours
pPLS::PLS:GFP
0 µM
1 µM
10 µM
Mean fluorescence intensity
110
100
90
80
70
60
50
40
30
20
10
0
2 h
24 h
2 h
24 h
2 h
24 h
0 µM
1 µM
10 µM
ACC treatment

Figure S15. The link between auxin and ethylene signalling effects on *PLS* transcription, and downstream effects on ethylene responses.

(**C**) **Expression of the PLS:GFP fusion protein is downregulated in the root tip after ACC treatment.**
Upper panel: Transgenic seedlings were treated with 0, 1 or 10 µM ACC for either 2 or 24 hours and imaged by CLSM at seven d.a.g. Laser settings were maintained at 21% 488 nm 20 mW, 970 V gain. Images are representative of 10 independetly grown roots. Scale bars = 50 µm.
Lower panel: Quantification of PLS-GFP fluorescence after ACC treatment, detected in seedlings expressing the *pPLS::PLS:GFP* construct. Seedling root tips at seven d.a.g were imaged by CLSM after treatment with 0, 1 or 10 µM ACC for either 2 or 24 hours. Fluorescence intensity in each root was measured using ImageJ and the mean intensity for each treatment was calculated. Error bars show ± 1 standard error, n = 17.

Supplementary Tables

Table S1. Differentially expressed genes between *pls* mutant and wild type (see video file).

Table S2. Differentially expressed genes between *PLS* transgenic overexpresser (PLSOx) and wild type (see video file).

Table S3. GO terms upregulated in *pls* mutant vs. wild type (see video file).

Table S4. GO terms downregulated in *pls* mutant vs. wild type (see video file).

Table S5. GO terms upregulated in *PLS* transgenic overexpresser (PLSOx) vs. wild type (see video file).

Table S6. GO terms downregulated in *PLS* transgenic overexpresser (PLSOx) vs. wild type (see video file).

Table S7. 120 GO terms unregulated in pls compared to wild type, and compared to PLSOx.

GO term	*pls* mutant upregulated cf WT GO term	FDR		GO term PLS OX downregulated cf WT	FDR
GO:0050896	response to stimulus	1.60E-37		response to stimulus	0.00013
GO:0006950	response to stress	3.90E-33		response to stress	0.34
GO:0006952	defense response	1.20E-28		defense response	1.0
GO:0044699	single-organism process	1.00E-25		single-organism process	5.5E-14
GO:1901700	response to oxygen-containing compound	1.30E-20		response to oxygen-containing compound	0.19
GO:0010243	response to organonitrogen compound	5.40E-20		response to organonitrogen compound	Not detected
GO:0042221	response to chemical	2.30E-19		response to chemical	0.012
GO:0010200	response to chitin	2.30E-19		response to chitin	Not detected
GO:0009987	cellular process	5.50E-17		cellular process	2.20E-20
GO:1901698	response to nitrogen compound	1.10E-16		response to nitrogen compound	1
GO:0044763	single-organism cellular process	4.00E-16		single-organism cellular process	1.80E-11
GO:0009605	response to external stimulus	5.20E-16		response to external stimulus	1
GO:0051707	response to other organism	2.10E-15		response to other organism	1
GO:0043207	response to external biotic stimulus	2.10E-15		response to external biotic stimulus	1
GO:0009617	response to bacterium	2.50E-15		response to bacterium	1
GO:0009607	response to biotic stimulus	5.10E-15		response to biotic stimulus	1
GO:0006468	protein phosphorylation	1.80E-13		protein phosphorylation	1
GO:0008152	metabolic process	4.00E-13		metabolic process	7.90E-11
GO:0002376	immune system process	1.00E-12		immune system process	1
GO:0044710	single-organism metabolic process	1.20E-12		single-organism metabolic process	1.60E-06
GO:0051704	multi-organism process	1.50E-12		multi-organism process	1
GO:0016310	phosphorylation	1.50E-12		phosphorylation	1
GO:0051716	cellular response to stimulus	1.90E-12		cellular response to stimulus	1
GO:0045087	innate immune response	3.50E-12		innate immune response	1
GO:0010033	response to organic substance	5.30E-12		response to organic substance	0.064
GO:0006955	immune response	8.30E-12		immune response	1
GO:0009628	response to abiotic stimulus	1.00E-11		response to abiotic stimulus	5.00E-14
GO:0009657	plastid organization	6.60E-11		plastid organization	6.00E-07
GO:0098542	defense response to other organism	1.50E-10		defense response to other organism	1
GO:0042742	defense response to bacterium	1.80E-10		defense response to bacterium	1
GO:0001101	response to acid chemical	2.10E-10		response to acid chemical	0.098

GO term	*pls* mutant upregulated cf WT GO term	FDR		GO term PLS OX downregulated cf WT	FDR
GO:0006793	phosphorus metabolic process	3.70E-10		phosphorus metabolic process	1
GO:0009719	response to endogenous stimulus	4.20E-10		response to endogenous stimulus	0.41
GO:0007154	cell communication	4.20E-10		cell communication	1
GO:0006796	phosphate-containing compound metabolic process	4.40E-10		phosphate-containing compound metabolic process	1
GO:0044711	single-organism biosynthetic process	2.00E-09		single-organism biosynthetic process	4.50E-06
GO:0009658	chloroplast organization	4.40E-09		chloroplast organization	4.20E-06
GO:0071704	organic substance metabolic process	1.00E-08		organic substance metabolic process	1.80E-06
GO:0007165	signal transduction	1.10E-08		signal transduction	1
GO:0043412	macromolecule modification	1.20E-08		macromolecule modification	1
GO:0044237	cellular metabolic process	2.00E-08		cellular metabolic process	3.40E-13
GO:0044700	single organism signaling	2.30E-08		single organism signaling	1
GO:0023052	signaling	2.40E-08		signaling	1
GO:0006979	response to oxidative stress	1.30E-07		response to oxidative stress	1
GO:0009611	response to wounding	2.10E-07		response to wounding	1
GO:0006464	cellular protein modification process	6.20E-07		cellular protein modification process	1
GO:0036211	protein modification process	6.20E-07		protein modification process	1
GO:0019748	secondary metabolic process	1.00E-06		secondary metabolic process	1
GO:0008219	cell death	1.70E-06		cell death	Not detected
GO:0009814	defense response, incompatible interaction	3.10E-06		defense response, incompatible interaction	Not detected
GO:0044550	secondary metabolite biosynthetic process	5.00E-06		secondary metabolite biosynthetic process	1
GO:0071456	cellular response to hypoxia	5.00E-06		cellular response to hypoxia	Not detected
GO:0043436	oxoacid metabolic process	6.70E-06		oxoacid metabolic process	0.036
GO:0000302	response to reactive oxygen species	8.00E-06		response to reactive oxygen species	0.73
GO:0052542	defense response by callose deposition	8.00E-06		defense response by callose deposition	Not detected
GO:0036294	cellular response to decreased oxygen levels	8.00E-06		cellular response to decreased oxygen levels	Not detected
GO:0071453	cellular response to oxygen levels	8.00E-06		cellular response to oxygen levels	Not detected
GO:0052482	defense response by cell wall thickening	1.60E-05		defense response by cell wall thickening	Not detected

GO term	*pls* mutant upregulated cf WT GO term	FDR		GO term PLS OX downregulated cf WT	FDR
GO:0052544	defense response by callose deposition in cell wall	1.60E-05		defense response by callose deposition in cell wall	Not detected
GO:0070887	cellular response to chemical stimulus	1.80E-05		cellular response to chemical stimulus	1
GO:0055114	oxidation-reduction process	2.60E-05		oxidation-reduction process	6.20E-05
GO:0010035	response to inorganic substance	2.70E-05		response to inorganic substance	0.00081
GO:0031347	regulation of defense response	2.90E-05		regulation of defense response	1
GO:0006082	organic acid metabolic process	3.10E-05		organic acid metabolic process	0.025
GO:0031425	chloroplast RNA processing	4.00E-05		chloroplast RNA processing	Not detected
GO:0044281	small molecule metabolic process	5.20E-05		small molecule metabolic process	0.0039
GO:0080134	regulation of response to stress	5.20E-05		regulation of response to stress	1
GO:0019752	carboxylic acid metabolic process	7.00E-05		carboxylic acid metabolic process	0.036
GO:0001666	response to hypoxia	0.0001		response to hypoxia	Not detected
GO:0042430	indole-containing compound metabolic process	0.0001		indole-containing compound metabolic process	Not detected
GO:0033554	cellular response to stress	0.00012		cellular response to stress	1
GO:0010193	response to ozone	0.00022		response to ozone	Not detected
GO:0036293	response to decreased oxygen levels	0.00022		response to decreased oxygen levels	Not detected
GO:0070482	response to oxygen levels	0.00024		response to oxygen levels	Not detected
GO:0009266	response to temperature stimulus	0.00025		response to temperature stimulus	1.30E-07
GO:0052545	callose localization	0.00025		callose localization	Not detected
GO:0012501	programmed cell death	0.00029		programmed cell death	Not detected
GO:0052543	callose deposition in cell wall	0.00029		callose deposition in cell wall	Not detected
GO:0009793	embryo development ending in seed dormancy	0.0003		embryo development ending in seed dormancy	1
GO:0009725	response to hormone	0.0003		response to hormone	0.22
GO:0052386	cell wall thickening	0.00035		cell wall thickening	Not detected
GO:0033037	polysaccharide localization	0.0004		polysaccharide localization	Not detected
GO:0009751	response to salicylic acid	0.00055		response to salicylic acid	1
GO:0009723	response to ethylene	0.00062		response to ethylene	1
GO:0044238	primary metabolic process	0.00063		primary metabolic process	2.70E-05
GO:0042391	regulation of membrane potential	0.00063		regulation of membrane potential	Not detected
GO:0009790	embryo development	0.00077		embryo development	1

GO term	*pls* mutant upregulated cf WT GO term	FDR		GO term PLS OX downregulated cf WT	FDR
GO:0065007	biological regulation	0.00099		biological regulation	0.5
GO:0010304	PSII associated light-harvesting complex II catabolic process	0.001		PSII associated light-harvesting complex II catabolic process	Not detected
GO:0006520	cellular amino acid metabolic process	0.0011		cellular amino acid metabolic process	1
GO:0010154	fruit development	0.0012		fruit development	1
GO:0032501	multicellular organismal process	0.0013		multicellular organismal process	0.068
GO:0009817	defense response to fungus, incompatible interaction	0.0013		defense response to fungus, incompatible interaction	Not detected
GO:0071369	cellular response to ethylene stimulus	0.0013		cellular response to ethylene stimulus	1
GO:0048583	regulation of response to stimulus	0.0013		regulation of response to stimulus	1
GO:0006801	superoxide metabolic process	0.0013		superoxide metabolic process	Not detected
GO:0048316	seed development	0.0014		seed development	1
GO:0042793	transcription from plastid promoter	0.0014		transcription from plastid promoter	Not detected
GO:0050789	regulation of biological process	0.0015		regulation of biological process	0.046
GO:0048731	system development	0.002		system development	0.18
GO:0050794	regulation of cellular process	0.0025		regulation of cellular process	0.025
GO:0007275	multicellular organism development	0.0031		multicellular organism development	0.019
GO:0009873	ethylene-activated signaling pathway	0.0032		ethylene-activated signaling pathway	1
GO:0016143	S-glycoside metabolic process	0.0032		S-glycoside metabolic process	Not detected
GO:0042343	indole glucosinolate metabolic process	0.0032		indole glucosinolate metabolic process	Not detected
GO:0009620	response to fungus	0.0033		response to fungus	1
GO:0000160	phosphorelay signal transduction system	0.0034		phosphorelay signal transduction system	1
GO:0043170	macromolecule metabolic process	0.0034		macromolecule metabolic process	0.038
GO:0009626	plant-type hypersensitive response	0.0035		plant-type hypersensitive response	Not detected
GO:0009816	defense response to bacterium, incompatible interaction	0.0037		defense response to bacterium, incompatible interaction	Not detected
GO:0016053	organic acid biosynthetic process	0.0037		organic acid biosynthetic process	0.023
GO:0048544	recognition of pollen	0.0037		recognition of pollen	Not detected

GO term	*pls* mutant upregulated cf WT GO term	FDR		GO term PLS OX downregulated cf WT	FDR
GO:0034050	host programmed cell death induced by symbiont	0.0037		host programmed cell death induced by symbiont	Not detected
GO:0044283	small molecule biosynthetic process	0.004		small molecule biosynthetic process	0.002
GO:0009636	response to toxic substance	0.004		response to toxic substance	1
GO:0071451	cellular response to superoxide	0.0044		cellular response to superoxide	Not detected
GO:0071450	cellular response to oxygen radical	0.0044		cellular response to oxygen radical	Not detected
GO:0098869	cellular oxidant detoxification	0.0044		cellular oxidant detoxification	Not detected
GO:1990748	cellular detoxification	0.0044		cellular detoxification	Not detected
GO:0019430	removal of superoxide radicals	0.0044		removal of superoxide radicals	Not detected

Table S8. Downregulated genes of GO term response to metal ion in pls mutant

GO term/gene	Protein function	FDR	Pvalue
GO:0010038 response to metal ion		8.5E-07	5.70E-05
AT5G24770	acid phosphatase activity dependent on the presence of divalent cations (Mg^{2+}, Co^{2+}, Zn^{2+}, Mn^{2+}) and anti-insect activity		
AT2G30860	GSTF9 glutathione S-transferase, metal responsive oxidative stress		
AT5G14545	MIR398b downregulated by biotic and abiotic stress		
AT1G11840	GLX1 glyoxalase/bleomycin resistance protein/ dioxygenase superfamily protein - link to water stress		
AT5G67300	MYBR1 mediates abiotic stress responses such as salt stress, oxidative stress		
AT3G56240	CCH copper chaperone involved in Cu homeostasis		
AT5G59780	MYB59 induced by cadmium (Cd) and plays a key role in the regulation of cell cycle progression and root elongation, controlling plant growth and stress responses		
AT4G25100	Fe-superoxide dismutase - required for oxidative stress tolerance		

GO term/gene	Protein function	FDR	Pvalue
AT3G13782	NAP1,4 - nucleosome assembly protein - required for resistance to genotoxic stresses such as UV		
AT1G35720	annexin and Ca transporter with peroxidase activity, links ROS and Ca signalling		
AT3G51860	CAX3 - a Ca transporter with role in phosphate homeostasis and metal sequestration		
AT1G07610	cysteine rich metallothionin required for heavy metal tolerance		
AT4G23670	Cu-binding polyketide cyclase/ dehydrase and lipid transport protein involved in abiotic stress		
AT3G16450	mannose-binding lectin involved in root cap salt stress response		
AT4G04460	PASPA3, Saposin-like aspartyl protease involved in PCD		
AT4G13430	methylthioalkyl malate isomerase involved in glucosinolate biosynthesis		
AT3G03780	putative methionine synthase		
AT1G07590	Tetratricopeptide repeat (TPR)-like superfamily protein - link to ABA signalling and osmotic stress		
AT4G37260	MYB73, role in salt stress		

Table S9.
Primers for RT-qPCR

Gene Forward/Reverse	Primer sequence 5'-3'	Tm (°C)
PLS Forward	CAGAGAGAAAGAGAAGAGCACG	58.5
PLS Reverse	TAATTTCAGGCGAAGGTCCAT	57.4
PLS pre-T-DNA Forward	GCAGTGTCTCACTGAAACATG	57.5
PLS pre-T-DNA Reverse	CAATGGATTTTAAAAAGTTTAAA CAATTTTGC	58.3
ERF11 Forward	AGCACCGTGGAATCATCGTT	60.04
ERF11 Reverse	CCATCACCACCGACGAAGAA	60.04
ERF19 Forward	CCACCGGTGAAAGTCAGTCA	59.89
ERF19 Reverse	TTCACGCTGGTACTGTGGAC	59.97
ERF61 Forward	CTCCGTCTATCTCCGCCAAC	59.97
ERF61 Reverse	GAACGATGGCATCCTCGCTA	59.97
TDR1 Forward	CAGAGATTCGAGACCCGTCG	59.97
TDR1 Reverse	AGCGAGATGACCCCTAAGGT	60.03
PP2C Forward	AGCAGGGTGAGGATTTGGTG	59.4
PP2C Reverse	ATTCACCTGGCAAATCCGGT	57.3
ACTIN2 Forward	GGATCGGTGGTTCCATTCTTGC	56
ACTIN2 Reverse	AGAGTTTGTCACACACAAGTGCA	55

Primers for *PLS::PLS* cloning from previously cloned sequence in TOPO2.1 (Casson et al. 2002)

PLS Forward:

GGGGACAAGTTTGTACAAAAAAGCAGGCTTCAAGCTTTAGCCCGTGCGG

PLS Reverse:

GGGGACCACTTTGTACAAGAAAGCTGGGTCATGGATTTTAAAAAGTTTAAACAATTTTGCTACTAATAA ATAAG

Primers for cloning *ETR1* and *PLS* sequences for yeast 2-hybrid and co-immuniprecipitation

ETR1:

Forward primer GAA TCC ATG GAA GTC TGC AAT TGT A (Eco RI on 5’ end)

Reverse primer GTC GAC TTA CAT GCC CTC GTA CA (Sal I on 5’end)

PLS:

Forward primer CTG GAG ATG AAA CCC AGA CTT TGT (Xho I on 5’ end)

Reverse primer GTC GAC ATG GAT TTT AAA AAG TTT (Sal I on 5’ end)
